# Supplementary material for: Examining patterns of multimorbidity, polypharmacy and risk of adverse drug reactions in chronic obstructive pulmonary disease: a cross-sectional UK Biobank study
Source: BMJ Open. 2018 Jan 14;8(1):e018404. doi: 10.1136/bmjopen-2017-018404 (PMC5781016; doi:10.1136/bmjopen-2017-018404)
Supplement: Supplementary file 1 [file bmjopen-2017-018404supp001.pdf]

| Comorbidity category (used in analysis) | Conditions included (as reported in table 2) | Self-reported conditions comprising this condition (UK Biobank variables used to identify self-reported conditions) |
|---|---|---|
| Cardiovascular conditions | Hypertension | Hypertension<br>Essential hypertension |
| | Coronary heart disease | Heart attack/MI<br>Angina |
| | Diabetes | Diabetic nephropathy<br>Diabetic neuropathy/ulcers<br>Diabetes<br>Type 1 diabetes<br>Type 2 diabetes<br>Diabetic eye disease |
| | Stroke/TIA | Stroke<br>TIA<br>Subarachnoid haemorrhage<br>Brain haemorrhage<br>Ischaemic stroke |
| | Atrial fibrillation | Atrial fibrillation |
| | Heart failure | Cardiomyopathy<br>Hypertrophic cardiomyopathy<br>Heart failure/pulmonary oedema |
| | Peripheral vascular disease | Peripheral vascular disease<br>Leg claudication/intermittent claudication |
| Respiratory | COPD | COPD/Chronic obstructive pulmonary disease<br>Emphysema/Chronic bronchitis<br>Emphysema |
| | Asthma | Asthma |
| | PE/DVT | Deep vein thrombosis<br>Pulmonary emolism |
| | Bronchiectasis | Bronchiectasis |
| | Pulmonary fibrosis | Pulmonary fibrosis |
| Cancer | Cancer | “yes”/”no” to “have you ever had cancer?” |
| Gastrointestinal | Dyspepsia | Gastro-oesophageal reflux (GORD)<br>Oesophagitis/Barrett’s oesophagus<br>Gastric stomach ulcers<br>Gastric erosions/gastritis<br>Duodenal ulcer<br>Dyspepsia/indigestion<br>Hiatus hernia<br>Helicobacter pylori |
| | Diverticular disease | Diverticular disease/diverticulitis |

| | | |
|---|---|---|
| | Irritable bowel syndrome | Irritable bowel syndrome |
| | Chronic liver disease | Oesophageal varices<br>Non infective hepatitis<br>Liver failure/cirrhosis<br>Primary biliary cirrhosis |
| | Inflammatory bowel disease | Inflammatory bowel disease<br>Crohn’s disease<br>Ulcerative colitis |
| | Constipation | Constipation |
| | Viral hepatitis | Hepatitis B<br>Hepatitis C<br>Hepatitis D |
| Mental Health | Depression | Depression<br>Postnatal depression |
| | Anxiety | Anxiety/panic attacks<br>Nervous breakdown<br>Post-traumatic stress disorder<br>Obsessive compulsive disorder<br>Stress<br>Insomnia<br>Psychological/psychiatric problem |
| | Schizophrenia | Scizophrenia |
| | Bipolar | Mania<br>Bipolar disorder<br>Manic depression |
| Painful conditions | Connective tissue diseases | Myositis/myopathy<br>Systemic lupus erythematosus/SLE<br>Connective tissue disorder<br>Sjogren’s syndrome.sicca syndrome<br>Dermatopolymyositis<br>Scloeroderma/systemic sclerosis<br>Rheumatoid arthritis<br>Psoriatic arthropathy<br>Dermatomyositis<br>Polymyositis<br>Polymyalgia rheumatica |
| | Other painful conditions | Back pain<br>Joint pain<br>Headaches (not migraine)<br>Sciatica<br>Plantar fasciitis<br>Carpal tunnel syndrome<br>Fibromyalgia<br>Arthritis<br>Shingles<br>Disc problem<br>Prolapsed disc/slipped disc |

| | | |
|---|---|---|
| | | Spine arthritis/spondylitis<br>Ankylosing spondylitis<br>Back problem<br>Osteoarthritis<br>Gout<br>Cervical spondylosis<br>Trigeminal neuralgia<br>Disc degeneration<br>Trapped nerve/compressed nerve |
| Other | Osteoporosis | Osteoporosis |
