# Supplementary material for: Examining patterns of multimorbidity, polypharmacy and risk of adverse drug reactions in chronic obstructive pulmonary disease: a cross-sectional UK Biobank study
Source: BMJ Open. 2018 Jan 14;8(1):e018404. doi: 10.1136/bmjopen-2017-018404 (PMC5781016; doi:10.1136/bmjopen-2017-018404)
Supplement: Supplementary file 2 [file bmjopen-2017-018404supp002.pdf]

Drugs with cumulative risk of Adverse Drug Reactions*	
Adverse Drug Reaction	Contributing drug classes (participants taking 3 or more considered ‘at risk’ for the purposes of analysis)
Falls	H2-receptor blockers<br>Loperamide<br>Prochlorperazine<br>Metoclopramide<br>ACE-inhibitor/Angiotensin receptor blocker<br>Thiazide diuretic<br>Loop diuretic<br>Amiloride/triamterene<br>Spironolactone<br>Beta-blocker<br>Calcium-channel blocker<br>Nitrates or nicorandil<br>Digoxin<br>Oral steroids<br>Opiates<br>Benzodiazepines<br>Sedative antihistamines<br>H1-receptor blockers<br>Antipsychotics<br>Tricyclic antidepressants<br>Sulfonylureas/gliptins/glinides<br>Pioglitazone<br>Urinary antispasmodics<br>Dosulepin<br>Alpha-blockers
Constipation	H2-receptor blockers<br>Laxatives<br>Loperamide<br>Prochlorperazine<br>Thiazide diuretics<br>Loop diuretics<br>Calcium-channel blockers<br>Opiates<br>Sedative antihistamines<br>H1-receptor blockers<br>Antipsychotics<br>Tricyclic antidepressants<br>Urinary antispasmodics<br>Dosulepin
Urinary retention	H2-receptor blockers<br>Loperamide<br>Prochlorperazine<br>Opiates<br>Sedative antihistamines<br>H1-receptor blockers<br>Antipsychotics<br>Tricyclic antidepressants

	Urinary antispasmodics<br>Dosulepin
CNS depression	H2-receptor blockers<br>Loperamide<br>Prochlorperazine<br>Oral steroids<br>Opiates<br>Benzodiazepines<br>Sedative antihistamines<br>H1-receptor blockers<br>Antipsychotics<br>Tricyclic antidepressants<br>Urinary antispasmodics<br>Dosulepin
Bleeding	Aspirin<br>Clopidogrel<br>Other antiplatelets<br>Oral steroids<br>SSRIs and related drugs<br>Non-steroidal anti-inflammatory drugs<br>Warfarin
Renal injury	ACE-inhibitor/angiotensin receptor blockers<br>Thiazide diuretic<br>Loop diuretic<br>Amiloride/triamterene<br>Spironolactone<br>Antibiotics/antifungals<br>Non-steroidal anti-inflammatory drugs
Adapted from *Scottish Government Model of Care Polypharmacy Working Group. Polypharmacy Guidance (2nd edition) March 2015. Scottish Government.*	
