# Supplementary material for: Examining patterns of multimorbidity, polypharmacy and risk of adverse drug reactions in chronic obstructive pulmonary disease: a cross-sectional UK Biobank study
Source: BMJ Open. 2018 Jan 14;8(1):e018404. doi: 10.1136/bmjopen-2017-018404 (PMC5781016; doi:10.1136/bmjopen-2017-018404)
Supplement: Supplementary file 3 [file bmjopen-2017-018404supp003.pdf]

Table S1. Odds ratios (with 95% CI) for the presence of categories of comorbidity				
Comorbidity category	Self-report COPD compared with no COPD N=502,640		GOLD COPD compared with no COPD N=496,943	
	Model 1 N=502,013	Model 2 N=487,718	Model 1 N=496,324	Model 2 N=482,378
	OR (95% CI)	OR (95% CI)	OR (95% CI)	OR (95% CI)
Cardiovascular disease	1.45 (1.39-1.52) ***	1.28 (1.22-1.34) ***	1.11 (1.02-1.20) *	1.08 (0.99-1.18) §
Cancer	1.29 (1.20-1.39) ***	1.22 (1.13-1.31) ***	1.12 (0.99-1.27) §	1.06 (0.92-1.19) §
Gastrointestinal disease	1.76 (1.67-1.86) ***	1.56 (1.48-1.65) ***	1.4 (1.26-1.54) ***	1.24 (1.12-1.38) ***
Mental health	2.02 (1.89-2.15) ***	1.62 (1.51-1.73) ***	1.75 (1.54-1.97) ***	1.40 (1.22-1.58) ***
Painful conditions	1.62 (1.55-1.70) ***	1.41 (1.34-1.48) ***	1.40 (1.28-1.52) ***	1.24 (1.13-1.35) **

§ : p>0.05 * : p<0.05, ** : p<0.01, *** : p<0.001
Model 1: Adjusted for age, sex and socioeconomic status
Model 2: Adjusted for age, sex, socioeconomic status, smoking, alcohol frequency, body mass index and physical activity

Table S2. Odds ratios (with 95% CI) for the presence of multimorbidity or polypharmacy				
Outcome	Self-report COPD compared with no COPD N=502,640		GOLD COPD compared with no COPD N=496,943	
	Model 1 N=502,013	Model 2 N=487,718	Model 1 N=496,324	Model 2 N=482,378
	OR (95% CI)	OR (95% CI)	OR (95% CI)	OR (95% CI)
Multimorbidity (≥4 conditions)	3.49 (3.28-3.70) ***	2.79 (2.61-2.98) ***	2.34 (2.10-2.63) ***	1.99 (1.75-2.25) ***
Polypharmacy (≥5 medications)	3.85 (3.68-4.03) ***	3.30 (3.15-3.46) ***	3.47 (3.20-3.75) ***	3.20 (2.95-3.48) ***
Polypharmacy (≥10 medications	5.72 (5.36-6.10) ***	4.42 (4.11-4.75) ***	4.20 (3.72-4.73) ***	3.56 (3.12-4.05) ***

§ : p>0.05 * : p<0.05, ** : p<0.01, *** : p<0.001
Model 1: Adjusted for age, sex and socioeconomic status
Model 2: Adjusted for age, sex, socioeconomic status, smoking, alcohol frequency, body mass index and physical activity

Table S3. Odds ratios (with 95% CI) for taking 3 of more medications associated with similar ADRs				
ADR	Self-report COPD compared with no COPD N=502,640		GOLD COPD compared with no COPD N=496,943	
	Model 1 N=502,013	Model 2 N=487,718	Model 1 N=496,943	Model 2 N=482,378
	OR (95% CI)	OR (95% CI)	OR (95% CI)	OR (95% CI)
Falls	2.27 (2.13 – 2.42) ***	1.83 (1.71-1.96) ***	1.66 (1.47 – 1.87) ***	1.49 (1.30-1.69) ***
Constipation	2.71 (2.54 – 2.89) ***	2.66 (2.39-2.96) ***	2.18 (1.77 – 2.64) ***	1.82 (1.47-2.24) ***
Urinary retention	3.38 (2.94 – 3.87) ***	2.59 (2.22-3.0) ***	1.98 (1.44 – 2.64) ***	1.64 (1.18-2.21) **
CNS Depression	3.75 (3.31 – 4.25) ***	2.81 (2.45-3.22) ***	2.29 (1.73 – 2.95) ***	1.87 (1.40-2.43) ***
Bleeding	4.60 (3.35 – 6.19) ***	3.39 (2.40-4.66) ***	2.63 (1.25 – 4.80) **	1.76 (0.75-3.48) §
Renal injury	2.22 (1.86 – 2.62) ***	1.84 (1.53-2.19) ***	1.94 (1.41 – 2.58) ***	1.84 (1.33-2.49) ***

§ : p>0.05 * : p<0.05, ** : p<0.01, *** : p<0.001
Model 1: Adjusted for age, sex and socioeconomic status
Model 2: Adjusted for age, sex, socioeconomic status, smoking, alcohol frequency, body mass index and physical activity

Subgroup analyses – comparing COPD with no COPD among participants with specific categories of comorbidity

**Table S4. Odds ratios (with 95% CI) for taking 3 of more medications associated with similar ADRs in participants with cardiovascular disease (CVD)**		
ADR	Self-report COPD plus CVD compared with CVD alone (no COPD)<br>N=156,848	GOLD COPD plus CVD compared with CVD alone (no COPD)<br>N=154,047
	Model 1<br>N=156,667	Model 1<br>N=153,852
	OR (95% CI)	OR (95% CI)
Falls	1.92 (1.79-2.07) ***	1.59 (1.39-1.82) ***
Constipation	2.89 (2.58-3.23) ***	2.06 (1.63-2.57) ***
Urinary retention	2.78 (2.33-3.28) ***	1.92 (1.30-2.72) ***
CNS Depression	3.17 (2.71-3.69) ***	2.17 (1.54-2.97) ***
Bleeding	4.00 (2.85-5.48) ***	2.26 (0.96-4.44) *
Renal injury	1.90 (1.59-2.25) ***	1.82 (1.31-2.45) ***
§ : p>0.05 * : p<0.05, ** : p<0.01, *** : p<0.001<br>Model 1: Adjusted for age, sex and socioeconomic status		

**Table S5. Odds ratios (with 95% CI) for taking 3 of more medications associated with similar ADRs in participants with cancer**		
ADR	Self-report COPD plus cancer compared with cancer alone (no COPD)<br>N=38,623	GOLD COPD plus cancer compared with cancer alone (no COPD)<br>N=37,958
	Model 1<br>N=38,575	Model 1<br>N= 37,912
	OR (95% CI)	OR (95% CI)
Falls	2.35 (1.95-2.81) ***	1.49 (1.00-2.13) *
Constipation	3.55 (2.73-4.56) ***	2.21 (1.22-3.68) **
Urinary retention	3.65 (2.52-5.13) ***	1.99 (0.78-4.14) §
CNS Depression	3.74 (2.66-5.14) ***	2.04 (0.86-4.04) §
Bleeding	4.69 (1.91-9.86) ***	2.20 (0.12-10.23) §
Renal injury	2.0 (1.17-3.20) **	2.26 (0.89-4.71) §
§ : p>0.05 * : p<0.05, ** : p<0.01, *** : p<0.001<br>Model 1: Adjusted for age, sex and socioeconomic status		

**Table S6. Odds ratios (with 95% CI) for taking 3 of more medications associated with similar ADRs in participants with gastrointestinal disease (GI)**		
ADR	Self-report COPD plus GI compared with GI alone (no COPD)<br>N=58372	GOLD COPD plus GI compared with GI alone (no COPD)<br>N=57103
	Model 1<br>N=58,299	Model 1<br>N=57,031
	OR (95% CI)	OR (95% CI)
Falls	2.18 (1.92-2.46) ***	1.46 (1.13-1.87) **
Constipation	2.70 (2.29-3.16) ***	1.58 (1.08-2.24) *
Urinary retention	2.64 (2.12-3.26) ***	1.46 (0.83-2.37) §
CNS Depression	3.02 (2.47-3.66) ***	1.50 (0.88-2.37) §
Bleeding	3.88 (2.27-6.25) ***	3.18 (0.97-7.63) §
Renal injury	1.99 (1.37-2.80) ***	1.22 (0.48-2.51) §
§ : p>0.05 * : p<0.05, ** : p<0.01, *** : p<0.001<br>Model 1: Adjusted for age, sex and socioeconomic status		

**Table S7. Odds ratios (with 95% CI) for taking 3 of more medications associated with similar ADRs in participants with mental health conditions (MH)**		
ADR	Self-report COPD plus MH compared with MH alone (no COPD)<br>N=36,949	GOLD COPD plus MH compared with MH alone (no COPD)<br>N=36126
	Model 1<br>N=36,885	Model 1<br>N=36,065
	OR (95% CI)	OR (95% CI)
Falls	2.21 (1.90-2.56) ***	1.35 (0.99-1.82) §
Constipation	2.33 (1.93-2.81) ***	1.62 (1.08-2.34) *
Urinary retention	2.17 (1.71-2.74) ***	1.42 (0.82-2.29) §
CNS Depression	2.53 (2.04-3.12) ***	1.66 (1.03-2.54) *
Bleeding	2.86 (1.77-4.17) ***	1.94 (0.76-4.05) §
Renal injury	1.86 (1.19-2.79) **	1.27 (0.45-2.80) §
§ : p>0.05 * : p<0.05, ** : p<0.01, *** : p<0.001<br>Model 1: Adjusted for age, sex and socioeconomic status		

**Table S8. Odds ratios (with 95% CI) for taking 3 of more medications associated with similar ADRs in participants with painful conditions**		
ADR	Self-report COPD plus painful conditions compared with painful conditions alone (no COPD)<br>N=83,992	GOLD COPD plus painful conditions compared with painful conditions alone (no COPD)<br>N=82,388
	Model 1<br>N=83,895	Model 1<br>N=82,294
	OR (95% CI)	OR (95% CI)
Falls	1.99 (1.79-2.19) ***	1.45 (1.19-1.75) ***
Constipation	2.54 (2.21-2.91) ***	1.50 (1.10-2.00) **
Urinary retention	2.46 (2.03-2.96) ***	1.11 (0.64-1.75) §
CNS Depression	2.71 (2.28-3.21) ***	1.40 (0.90-2.06) §
Bleeding	3.50 (2.37-5.01) ***	2.20 (0.86-4.54) §
Renal injury	1.66 (1.30-2.09) ***	1.49 (0.93-2.25) §
§ : p>0.05 * : p<0.05, ** : p<0.01, *** : p<0.001<br>Model 1: Adjusted for age, sex and socioeconomic status		
