# Supplementary material for: Examining patterns of multimorbidity, polypharmacy and risk of adverse drug reactions in chronic obstructive pulmonary disease: a cross-sectional UK Biobank study
Source: BMJ Open. 2018 Jan 14;8(1):e018404. doi: 10.1136/bmjopen-2017-018404 (PMC5781016; doi:10.1136/bmjopen-2017-018404)
Supplement: Supplementary file 4 [file bmjopen-2017-018404supp004.pdf]

Appendix 4: Specific medications in UK Biobank participants with and without COPD						
**Medications**	**Control** n=494323 count (%)	**Self-report COPD** n=8317 count (%)	**GOLD COPD**			
			**All** n=2620 count (%)	**Mild** n=399 count (%)	**Moderate** n=1409 count (%)	**Severe** n= 812 count (%)
**Total number of medications**						
≥1	356406 (72.1)	7670 (92.2)	2452 (93.6)	352 (88.2)	1321 (93.8)	779 (95.9)
≥5	87286 (17.7)	4312 (51.8)	1349 (51.5)	171 (42.9)	702 (49.8)	476 (58.6)
≥10	10678 (2.2)	1269 (15.3)	329 (12.6)	31 (7.8)	172 (12.2)	126 (15.5)
**Respiratory**						
Short acting B_2 agon.	22615 (4.6)	3328 (40.0)	1245 (47.5)	123 (30.8)	614 (43.6)	508 (62.6)
LABA	9819 (2.0)	2357 (28.3)	905 (34.5)	93 (23.3)	411 (29.2)	401 (49.3)
LAMA	597 (0.1)	1345 (16.2)	581 (22.2)	33 (8.3)	265 (18.8)	283 (34.9)
ICS	15309 (3.1)	2638 (31.7)	962 (36.7)	98 (24.6)	471 (33.4)	393 (48.4)
LABA+ICS	7259 (1.5)	1842 (22.1)	699 (26.7)	67 (16.8)	313 (22.2)	319 (39.3)
Prednisolone	3127 (0.6)	280 (3.4)	82 (3.1)	12 (3.0)	27 (1.9)	43 (5.3)
Mucolytic	174 (0.04)	187 (2.2)	49 (1.9)	1 (0.3)	10 (0.7)	38 (4.7)
**Cardiovascular**						
Antiplatelet	21817 (4.4)	894 (10.7)	268 (10.2)	31 (7.8)	158 (11.2)	79 (9.7)
ACE-inhibitor	44991 (9.1)	1276 (15.3)	367 (14.0)	33 (8.3)	198 (14.1)	136 (16.7)
ARB	17911 (3.6)	565 (6.8)	159 (6.1)	17 (4.3)	83 (5.9)	59 (7.2)
Calcium CB	14317 (2.9)	627 (7.5)	196 (7.5)	23 (5.8)	106 (7.5)	67 (8.3)
Statin	73439 (14.9)	2278 (27.4)	707 (27.0)	72 (18.0)	395 (28.0)	240 (29.6)
GTN	4425 (0.9)	373 (4.5)	110 (4.2)	8 (2.0)	70 (5.0)	32 (3.9)
ISMN	2814 (0.6)	244 (2.9)	68 (2.6)	5 (1.3)	42 (3.0)	21 (2.6)
Loop diuretic	4836 (1.0)	415 (5.0)	107 (4.1)	10 (2.5)	51 (3.6)	46 (5.7)
Thiazide	21961 (4.4)	637 (7.7)	196 (7.5)	22 (5.5)	108 (7.7)	66 (8.1)
Warfarin	4934 (1.0)	238 (2.9)	67 (2.6)	6 (1.5)	33 (2.3)	28 (3.4)
**Diabetes**						
Insulin	4643 (0.9)	161 (1.9)	35 (1.3)	2 (0.5)	23 (1.6)	3 (0.4)
Metformin	13754 (2.8)	448 (5.4)	102 (3.9)	7 (1.8)	57 (4.0)	38 (4.7)
Sulphonylurea	4901 (1.0)	158 (1.9)	35 (1.3)	2 (0.5)	17 (1.2)	16 (2.0)
Thiazolidindione	2212 (0.4)	60 (0.7)	17 (0.6)	1 (0.3)	10 (0.7)	6 (0.7)
**Gastrointestinal**						
PPI	42012 (8.5)	1989 (23.9)	522 (19.9)	79 (19.8)	286 (20.3)	157 (19.3)
Antacid	2435 (0.5)	146 (1.8)	25 (1.0)	7 (1.8)	10 (0.7)	8 (1.0)
H_2 RA.	7772 (1.6)	325 (3.9)	89 (3.4)	15 (3.8)	53 (3.8)	21 (2.6)
Laxative	5787 (1.8)	317 (3.8)	81 (3.1)	11 (2.8)	40 (2.8)	30 (3.7)
**Pain**						
Paracetamol	82376 (16.7)	2752 (33.1)	790 (30.2)	111 (27.8)	446 (31.6)	233 (28.7)
NSAID	45909 (9.3)	1149 (13.8)	319 (12.2)	50 (12.5)	175 (12.4)	94 (11.6)
Weak opiate	18736 (3.8)	1209 (14.5)	336 (12.8)	48 (12.0)	191 (13.6)	97 (11.9)
Strong opiate	1071 (0.2)	106 (1.8)	32 (1.2)	5 (1.3)	16 (1.1)	11 (1.4)
**Mental health**						
SSRI+related	15394 (3.1)	747 (9.0)	175 (6.7)	31 (7.8)	100 (7.1)	44 (5.4)
Tricyclic	4229 (0.9)	206 (2.5)	49 (1.9)	11 (2.8)	25 (1.8)	13 (1.6)
Antipsychotic	2237 (0.5)	107 (1.3)	30 (1.1)	5 (1.3)	16 (1.1)	9 (1.1)
Benzodiazepine	2316 (0.5)	182 (2.2)	47 (1.8)	6 (1.5)	28 (2.0)	13 (1.6)
**Metabolic**						
Thyroxine	20980 (4.2)	560 (6.7)	150 (5.7)	27 (6.8)	92 (6.5)	31 (3.8)
Bisphosphonate	3655 (0.7)	189 (2.3)	66 (2.5)	15 (3.8)	32 (2.3)	19 (2.3)
