# Supplementary material for: Examining patterns of multimorbidity, polypharmacy and risk of adverse drug reactions in chronic obstructive pulmonary disease: a cross-sectional UK Biobank study
Source: BMJ Open. 2018 Jan 14;8(1):e018404. doi: 10.1136/bmjopen-2017-018404 (PMC5781016; doi:10.1136/bmjopen-2017-018404)
Supplement: Author's manuscript [file bmjopen-2017-018404.draft_revisions.pdf]

BMJ Open

BMJ Open is committed to open peer review. As part of this commitment we make the peer review history of every article we publish publicly available.

When an article is published we post the peer reviewers' comments and the authors' responses online. We also post the versions of the paper that were used during peer review. These are the versions that the peer review comments apply to.

The versions of the paper that follow are the versions that were submitted during the peer review process. They are not the versions of record or the final published versions. They should not be cited or distributed as the published version of this manuscript.

BMJ Open is an open access journal and the full, final, typeset and author-corrected version of record of the manuscript is available on our site with no access controls, subscription charges or pay-per-view fees (http://bmjopen.bmj.com).

If you have any questions on BMJ Open's open peer review process please email editorial.bmjopen@bmj.com

BMJ Open

Examining Patterns of Multimorbidity, Polypharmacy and Risk of Adverse Drug Reactions in Chronic Obstructive Pulmonary Disease: A Cross-Sectional UK Biobank Study

Journal:	*BMJ Open*
Manuscript ID	bmjopen-2017-018404
Article Type:	Research
Date Submitted by the Author:	27-Jun-2017
Complete List of Authors:	Hanlon, Peter; University of Glasgow Institute of Health and Wellbeing<br>Nicholl, Barbara; University of Glasgow, General Practice and Primary Care<br>Jani, Bhautesh; University of Glasgow<br>McQueenie, Ross; University of Glasgow, GPPC, School of Medicine, Dentistry and Nursing, MVLS<br>Lee, Duncan; University of Glasgow<br>Gallacher, Katie I; University of Glasgow,<br>Mair, Frances; University of Glasgow, General Practice and Primary Care
**Primary Subject Heading**:	Respiratory medicine
Secondary Subject Heading:	General practice / Family practice
Keywords:	Multimorbidity, Polypharmacy, Chronic airways disease < THORACIC MEDICINE, Adverse events < THERAPEUTICS

SCHOLARONE™
Manuscripts

Examining Patterns of Multimorbidity, Polypharmacy and Risk of Adverse Drug Reactions in Chronic Obstructive Pulmonary Disease: A Cross-Sectional UK Biobank Study

Corresponding author

Professor Frances Mair

General Practice and Primary Care, Institute of Health and Wellbeing, University of Glasgow, 1 Horelethill Road, Glasgow, G12 9LX, Scotland, United Kingdom

Tel: +44 141 330 8317

E-mail: frances.mair@glasgow.ac.uk

Authors:

Peter Hanlon[1], Barbara I. Nicholl[1], Bhautesh Dinesh Jani[1], Ross McQueenie[1], Duncan Lee[2], Katie I. Gallacher[1], Frances S Mair[1]

1. General Practice and Primary Care, Institute of Health and Wellbeing, University of Glasgow, 1 Horelethill Road, Glasgow, G12 9LX, Scotland, United Kingdom
2. School of Mathematics and Statistics, The mathematics and Statistics Building, University of Glasgow, University Place, Glasgow, G12 8SQ

Word count: 3446

For peer review only

Abstract

Objective: This study aims: (1) to describe the pattern and extent of multimorbidity and polypharmacy in UK Biobank participants with chronic obstructive pulmonary disease (COPD); and (2) to identify which comorbidities are associated with increased risk of adverse drug reactions (ADRs) resulting from polypharmacy.

Design: Cross-sectional analysis of UK Biobank.

Setting: Community cohort.

Participants: UK Biobank participants comparing self-reported COPD (n=8317) with no COPD (n=494,323).

Outcomes: Multimorbidity (≥ four conditions) and polypharmacy (≥ five medications) in participants with COPD versus those without. Risk of ADRs (taking ≥ three medications associated with falls, constipation, urinary retention, central nervous system (CNS) depression, bleeding or renal injury) in relation to the presence of COPD and individual comorbidities.

Results: Multimorbidity was more common in participants with COPD than those without (17% vs. 4%). Polypharmacy was highly prevalent (52% with COPD taking ≥ five medications vs 18% in those without COPD). Adjusting for age, sex and socioeconomic status, those with COPD were significantly more likely than those without to be prescribed ≥3 medications contributing to falls (Odds ratio (OR) 2.27, 95% confidence interval (CI) 2.13 to 2.42), constipation (OR 3.42, 95% CI 3.10 to 3.77), urinary retention (OR 3.38, 95% CI 2.94 to 3.87), CNS depression (OR: 3.75, 95% CI 3.31 to 4.25), bleeding (OR 4.61, 95% CI 3.35 to 6.19) and renal injury (OR 2.22, 95% CI 1.86 to 2.62). Comorbid cardiovascular disease was associated with the greatest risk of taking ≥3 medications associated with falls/renal injury. Comorbid mental health conditions were most strongly associated with medications linked with CNS depression/urinary retention/bleeding.

Conclusions: Multimorbidity is common in COPD and associated with high levels of polypharmacy. Co-prescription of drugs with various ADRs is common. Medications contributing to this risk are largely indicated for the management of associated comorbidities rather than COPD. Future

For peer review only

research should examine the effects on healthcare outcomes of co-prescribing multiple drugs with similar potential ADRs. COPD clinical guidelines should emphasise assessment of comorbidities and risk of ADRs.

Abstract word count: 325

Strengths and Limitations

- This paper assesses multimorbidity, polypharmacy and risk of adverse drug reactions are assessed in UK Biobank participants with self-reported COPD compared with those without COPD.
- Baseline variables from the UK Biobank assessment centre were used to adjust for potential confounders.
- Cumulative risk of common adverse drug reactions was quantified by identifying UK Biobank participants taking three or more medications associated with similar adverse drug reactions.
- Analyses were repeated using a subgroup of participants with spirometry data confirming airflow obstruction.
- Medication and comorbidity data rely on participant self-report, and may thus be susceptible to bias or inaccuracy.

For peer review only - http://bmjopen.bmj.com/site/about/guidelines.xhtml

BACKGROUND

In people with Chronic Obstructive Pulmonary Disease (COPD), multimorbidity (the presence of two or more long-term conditions (LTCs)) is highly prevalent.(1-4) A recent meta-analysis of 29 datasets demonstrated that those with COPD are significantly more likely to be diagnosed with a range of cardiovascular comorbidities than those without COPD (we will use the term comorbidity when referring to specific conditions in addition to COPD, and multimorbidity to refer to the presence of two or more LTCs).(5) Other LTCs with known increased prevalence in COPD include obesity,(6) depression,(7-10) gastro-oesophageal reflux disease,(11-13) osteoporosis,(14-16) and lung cancer.(17, 18) Each of these comorbidities has been associated with poorer health related outcomes in COPD when compared to those with no comorbidity.(19-30) The overall burden of multimorbidity also impacts prognosis in COPD, for example higher number of comorbidities is associated with higher risk of mortality,(31) and higher burden of morbidity assessed using the Charlson index and the COPD-specific comorbidity test (COTE) is associated with higher risk of all-cause and respiratory specific mortality.(32, 33)

Multimorbidity in the general population is associated with polypharmacy (often defined as concomitant use of ≥5 or ≥10 pharmacological agents).(34) Polypharmacy has been associated with increased risk of adverse drug reactions (ADRs)(35-37) and potentially preventable hospital admissions, particularly in the elderly.(38, 39) It has been demonstrated that diagnosis of COPD is associated with increased risk of polypharmacy.(40, 41) This is, in large measure, due to the high burden of extra-pulmonary comorbidities.(42) However, little is known about the risk of ADRs in the context of multimorbidity in COPD.

For peer review only - http://bmjopen.bmj.com/site/about/guidelines.xhtml

Given the well-established burden of multimorbidity and polypharmacy in COPD, it is likely that those with COPD are at increased risk of ADRs resulting from polypharmacy. Previous analyses have not focused on the risk of specific ADRs, or assessed which comorbidities increase this risk, instead reporting overall counts of prescribed medication. Data collected for the UK Biobank cohort offers an opportunity to assess how multimorbidity in COPD relates to polypharmacy and to assess the prevalence of co-prescription of medications with similar ADRs.

This paper aims:

- To describe the pattern and extent of multimorbidity and polypharmacy in UK Biobank participants with COPD.
- To identify which comorbidities in people with COPD are associated with increased risk of ADRs resulting from polypharmacy.

For peer review only

For peer review only - http://bmjopen.bmj.com/site/about/guidelines.xhtml

METHODS

Data collection

Between 2006 and 2010, UK Biobank recruited 502,640 participants aged 37 to 73. Participants underwent baseline assessments at one of 22 assessment centres throughout England, Scotland and Wales. Sociodemographic and lifestyle details were recorded using touchscreen questionnaires. Townsend scores were derived from participant postcodes to provide an area-based measure of socioeconomic deprivation. Self-reported LTCs, prescribed and over-the-counter medications, smoking status (current, previous or never) and frequency of alcohol intake (never / special occasions only, one-three times a month, at least once a week) were recorded from a touchscreen questionnaire and subsequent verbal interview with a study nurse. Physical activity was self-reported and classified into: none (no physical activity in the last four weeks), low (light 'DIY' activity only in the last four weeks), medium (heavy DIY and/or walking for pleasure and/or other exercises in the last four weeks), high (strenuous sports in the last four weeks).

Study centre staff also collected physical measures including height and weight (to calculate body mass index (BMI)) and spirometry. Spirometry was performed using a Vitalograph Pneumotrac 6800. Individual reasons for contraindications to attempting spirometry were not recorded but, according to protocol, these included chest infection in the last month, history of collapsed lung, and heart attack or surgery in the past three months. Full details of the Biobank spirometry protocol are available at https://biobank.ctsu.ox.ac.uk/crystal/docs/Spirometry.pdf. In brief, participants were allowed up to three attempts to provide two reproducible spirometry measurements. Where the reproducibility of the first two was deemed acceptable (<5% variation in both FEV1 and FVC) a third measurement was not performed. All values were recorded along with any error messages generated. As per the American Thoracic Society/European Respiratory Society (ATS/ERS) end-of-test criteria, we interpreted as valid any measurement with no error message or if 'user accepted' was specified.(43) No post-bronchodilator measurements were recorded, which deviates from the

For peer review only - http://bmjopen.bmj.com/site/about/guidelines.xhtml

ATS/ERS guidelines and the Global Initiative for Chronic Obstructive Lung Disease (GOLD) guidelines for COPD.(44, 45)

Participants provided full informed consent to participate in UK Biobank and this study had full ethical approval from the NHS National Research Ethics Service for UK Biobank studies (Ref 16/NW/0274).

Defining COPD

Participants reporting to have been diagnosed with chronic obstructive pulmonary disease, chronic bronchitis, or emphysema at the nurse-led interview were coded as having 'self-reported COPD'.

Due to the potential inaccuracies of using self-reported diagnoses, we identified a subset of those with self-reported COPD who met an adaptation of the Global Initiative for Obstructive Lung Disease (GOLD) spirometry criteria for COPD.(45) This subset, referred to as 'GOLD COPD', was used as a sensitivity analysis for self-reported COPD, and to stratify findings by severity of airflow obstruction. For participants with self-report COPD and valid spirometry measurements, we calculated the ratio of forced expiratory volume in 1 second (FEV1) to forced vital capacity (FVC) using the highest measurement for each participant meeting the American Thoracic Society/European Respiratory Society end-of-test criteria.(43) Those with a FEV1/FVC ratio <0.7 were classed as having an obstructive deficit and thus meeting the GOLD diagnostic criteria for COPD. We used the Hankinson equation,(46) based on recorded age, sex and height, to calculate predicted FEV1 values for each participant. Those with GOLD COPD were classified on the basis of their best available FEV1 measurement as having mild (>80% predicted FEV1), moderate (50-80% predicted FEV1), or severe (<50% predicted FEV1) airflow obstruction in line with the GOLD COPD guidelines.(45)

For peer review only - http://bmjopen.bmj.com/site/about/guidelines.xhtml

Defining comorbidities and medications

All morbidities were defined by self-report. For the purposes of examining the number of comorbidities reported, a count (1,2,3,4 or ≥5) was taken from a list of 42 morbidities originally established for a large epidemiological study in Scotland, through systematic review, the Quality and Outcomes Framework, NHS Scotland and an expert panel (47), and subsequently amended for UK Biobank (48). Morbidities were categorised for the purposes of this analysis into cardiovascular disease, gastrointestinal disease, mental health conditions, cancer, and painful conditions. Full details of conditions comprising each category can be found in appendix 1.

Medication data were collected by self-report. Medications were coded by mechanism of action according to the British National Formulary (BNF) (e.g. Angiotensin-converting enzyme inhibitors, beta-blockers, calcium channel blockers, etc.). For some situations where more than one medication with a similar mechanism of action may be commonly co-prescribed (e.g. aspirin and clopidogrel, both antiplatelets) these were coded separately. A complete list of the medications coded within each class can be found in appendix 2.

We defined those at risk of specific ADRs as anyone on 3 or more medications with similar potential ADRs, based on information provided in the *Scottish Government Model of Care Polypharmacy Working Group: Polypharmacy Guidance*.(49) This guideline cross-tabulates commonly prescribed medications with common ADRs to help identify those at cumulative risk of ADRs. This document groups common medications by similar potential ADRs. While this list is not all-inclusive, and the cut-off value of three or more medications is arbitrary, this does allow an estimation of the cumulative risk of specific ADRs. We identified six potential ADRs (falls/fractures, constipation, urinary retention, CNS depression, bleeding and renal injury) for which the proportion of participants taking three or more associated medications could be assessed.

For peer review only - http://bmjopen.bmj.com/site/about/guidelines.xhtml

Statistical analysis

Study hypothesis was made an analyses planned prior to inspection of the data.

Baseline variables

Comparisons were made between participants with self-reported COPD and the rest of the cohort (who did not report COPD). Age, sex, smoking status, deprivation (Townsend score), BMI, physical activity and frequency of alcohol intake were compared using χ^2 test for categorical variables, χ^2 test for trend for ordinal variables, and Mann-Whitney-U test for continuous variables. Total number of morbidities, prevalence of specific morbidities, number of self-reported prescribed medications, and proportion of participants taking each class of medication (Appendix 2), were also compared between those with self-reported COPD and the rest of the cohort. All comparisons were repeated comparing participants with GOLD COPD only with those without COPD, stratifying by severity of airflow obstruction.

Multimorbidity and polypharmacy

Logistic regression analyses were used to compare participants with self-reported COPD and those without COPD. Odds ratios (OR) and 95% confidence intervals (95% CI) were calculated for:

- the presence of cardiovascular disease, cancer, gastrointestinal disease, mental health conditions and painful conditions
- the presence of four or more morbidities (excluding COPD)
- the use of five or more, and 10 or more, medications (two separate models)

Models were initially adjusted for age, sex and socioeconomic deprivation (model 1), then adjusted for the addition of smoking status, alcohol frequency, BMI and physical activity (model 2). These analyses were repeated comparing those with GOLD COPD only to those without COPD.

Risk of ADRs

For peer review only - http://bmjopen.bmj.com/site/about/guidelines.xhtml

For each potential ADR (falls/fractures, constipation, urinary retention, CNS depression, bleeding and renal injury) participants taking three or more medications associated with that ADR were identified. The following comparisons were then made:

- Unadjusted percentages at risk of each ADR were calculated for participants without COPD, with self-reported COPD, and with self-reported COPD plus each category of comorbidity (cardiovascular disease, cancer, gastrointestinal disease, mental health conditions and painful conditions) to give an impression of the ADR risk in COPD, and identify comorbidities that may increase this risk.
- ORs of being at risk of each ADR were calculated comparing those with self-reported COPD to those without COPD adjusting for age, sex and socioeconomic deprivation (model 1) and for age, sex, socioeconomic deprivation, smoking status, alcohol frequency, BMI and physical activity (model 2).
- ORs of being at risk of each ADR were calculated comparing those with and without self-reported COPD in each comorbidity category to (i.e. participants with cardiovascular disease alone compared to participants with cardiovascular disease plus COPD, etc.). This was intended to identify whether specific patterns of multimorbidity in COPD are associated with increased ADR risk. Adjustment for a wide range of potential confounders was not appropriate in these models due to the smaller number of participants in each model.

Each analysis was repeated comparing GOLD COPD only to those without COPD. Less than 3% of participants (with or without COPD) had missing data for potential confounding variables (table 1). Those with missing data were excluded from adjusted analyses. Spirometry data were missing for 3591 participants with self-report COPD (43%), hence the use of the GOLD COPD subset as a sensitivity analysis.

All analyses were performed using R statistical software (version 3.3.1).

For peer review only - http://bmjopen.bmj.com/site/about/guidelines.xhtml

RESULTS

At the time of recruitment, 8317 participants reported having COPD (1.7%) and are referred to here as the self-report COPD group. Of those who self-reported COPD, 4726 (57%) had valid spirometry measurements. Spirometry was contraindicated or not available in 2507 of those with self-reported COPD. Spirometry measurements did not meet the ATS/ESR end-of-test criteria in 1084 participants.(43) Of those with valid spirometry, 2620 (55%) met the GOLD criteria for airflow obstruction (399 (15%) mild, 1409 (54%) moderate, 812 (31%) severe, see Figure 1) and are referred to here as GOLD COPD.

Baseline variables

Table 1 describes and compares the characteristics of those with and without COPD in UK Biobank. Participants with COPD (both self-report and GOLD) were significantly older, more socioeconomically deprived, and less physically active. A higher proportion of those with COPD were male, obese and had a history of smoking.

For peer review only

For peer review only - http://bmjopen.bmj.com/site/about/guidelines.xhtml

Table 1. Baseline variables								
**Characteristic**	**No COPD n=494323**		**COPD (self-report) n=8317**			**GOLD GOPD n=2620**		
	Count	%	Count	%	p-value	Count	%	p-value
**Sex**								
Male	224906	45.5	4268	51.3		1426	54.4	
Female	269417	54.5	4049	48.7	<0.001	1194	45.6	<0.001
**Age**	Median: 58		Median: 62			Median:		
	IQR: 50-63		IQR: 57-66		<0.001	63		<0.001
						IQR: 59-66		
**Ethnicity**								
White	464770	94.5	8052	97.3		2620	100	
Other	26821	5.4	219	2.6	<0.001	0	0	<0.001
Missing	2732		46			0	0	
**Socioeconomic**								
**deprivation**								
**quintile**	99672	20.2	1015	12.2		309	11.8	
1 (least deprived)	98977	20.0	1142	13.7		362	13.8	
2	99013	20.1	1399	16.8		440	16.8	
3	98660	20.0	1735	20.9		580	22.2	
4	98385	19.7	3015	36.3	<0.001	926	35.4	<0.001
5 (most deprived)	616		11			3		
Missing								
**Smoking status**								
Current	50817	10.3	2172	26.3		833	32.2	
Previous	169015	34.4	4083	49.5		1398	54.0	
Never	271602	55.3	1999	24.2	<0.001	360	13.9	<0.001
Missing	2889		63			29		
**Alcohol**								
**frequency**	100070	20.3	1720	20.7		618	23.6	
Daily	114058	23.1	1404	16.9		475	18.2	
3-4 times/week	127459	25.9	1863	22.5		561	21.5	
1-2 times/week	54979	11.2	894	10.8		289	11.1	
1-3 times/month	56707	11.5	1322	15.9		387	14.8	
Occasional	39569	8.0	1092	13.2	<0.001	284	10.9	<0.001
Never	1481		22			6		
Missing								
**BMI**								
<18.5	2478	0.5	148	1.8		56	2.2	
18.5-24.9	155282	31.8	2185	26.8		829	31.9	
25.0-29.9	211102	43.2	3165	38.9		1049	40.4	
>30	119813	24.5	2647	32.5	<0.001	665	25.6	<0.001
Missing	5648		172			21		
**Physical activity**								
High	49827	10.6	250	3.1		70	2.7	
Medium	387766	79.6	5838	72.0		1902	73.3	
Low	18354	3.8	589	7.3		203	7.8	
None	31425	6.4	1433	17.7	<0.001	421	16.2	<0.001
Missing	6951		207			24		
**FEV1**								
**(% predicted)**								
>80	272109	78.0	1853	39.2		399	15.2	
50-79	71727	20.6	2022	42.8		1409	53.8	
<50	4841	1.4	851	18.0	<0.001	812	31.0	<0.001
Missing	145646		3591			1061		

For peer review only - http://bmjopen.bmj.com/site/about/guidelines.xhtml

Multimorbidity and polypharmacy

Prevalence of each category of comorbidity was higher in those with COPD than without (table 2). After controlling for age, sex and socioeconomic status, those with self-reported COPD were significantly more likely than those without to have each category of comorbidity examined: cardiovascular disease (OR 1.45; 95% confidence interval (CI) 1.39 to 1.52) , cancer (1.29; 1.2 to 1.39), gastrointestinal disease (1.76; 1.67 to 1.86) , mental health conditions (2.02; 1.89 to 2.15) , and painful conditions (1.54; 1.46 to 1.62). Results for GOLD COPD also suggested higher likelihood of each comorbidity compared to those without COPD, although the ORs were lower and results for cancer not statistically significant (appendix 3). Results were similar after adjusting for additional confounders (smoking status, alcohol frequency, BMI and physical activity) with the exception of cardiovascular comorbidity in GOLD COPD, which was no longer significantly associated (1.08; 0.99 to 1.18) (appendix 3).

For peer review only

For peer review only - http://bmjopen.bmj.com/site/about/guidelines.xhtml

Table 2: Comorbities in those with and without COPD						
	**Control**	**Self-report COPD**	**GOLD COPD**			
			**All**	**Mild**	**Moderate**	**Severe**
	n=494323<br>count (%)	n=8317<br>count (%)	n=2620<br>count (%)	n=399<br>count (%)	n=1409<br>count (%)	n= 812<br>count (%)
**Total comorbid-ities (excluding COPD)**						
≥4	19959 (4.0)	1389 (16.7)**	331 (12.6)**	46 (11.5)	191 (13.5)	94 (11.6)
**Total number of medications**						
≥1	356406 (72.1)	7670 (92.2)**	2452 (93.6)**	352 (88.2)	1321 (93.8)	779 (95.9)
≥5	87286 (17.7)	4312 (51.8)**	1349 (51.5)**	171 (42.9)	702 (49.8)	476 (58.6)
≥10	10678 (2.2)	1269 (15.3)**	329 (12.6)**	31 (7.8)	172 (12.2)	126 (15.5)
**Prevalence of comorbidities**						
**Cardiovascular**	**152891 (30.9)**	**3957 (47.6)****	**1156 (44.1)****	**142 (35.6)**	**611 (43.4)**	**403 (49.6)**
Hypertension	130119 (26.3)	3206 (38.5)**	916 (35.0)**	112 (28.1)	483 (34.3)	321 (39.5)
CHD	21560 (4.4)	1171 (14.1)**	315 (12.0)**	31 (7.6)	185 (13.1)	99 (12.2)
Diabetes	24737 (5.0)	766 (9.1)**	189 (7.2)**	16 (4.0)	109 (7.7)	64 (7.9)
Stroke/TIA	8459 (1.7)	395 (4.7)**	98 (3.7)**	11 (2.8)	51 (3.6)	36 (4.4)
AF	3552 (0.7)	99 (1.2)**	34 (1.3)**	3 (0.8)	16 (1.1)	15 (1.8)
Heart failure	768 (0.2)	35 (0.4)**	6 (0.2)	0	1 (0.1)	5 (0.6)
**Respiratory**						
Asthma	55245 (11.2)	3048 (36.6)**	984 (37.6)**	142 (35)	523 (37.1)	319 (39.3)
PE/DVT	12316 (2.5)	554 (6.7)**	139 (5.3)**	29 (7.3)	71 (5.0)	39 (4.8)
Bronchiectasis	968 (0.2)	167 (2.0)**	39 (1.5)**	7 (1.8)	17 (1.2)	15 (1.8)
Pulmonary fib.	504 (0.1)	67 (0.8)**	18 (0.7)**	3 (0.8)	12 (0.9)	3 (0.4)
**Cancer**	**37686 (7.6)**	**937 (11.3)**	**272 (10.4)**	**47 (11.8)**	**146 (10.4)**	**79 (9.7)**
Lung	405 (0.1)	52 (0.6)**	15 (0.6)**	0	7 (0.5)	8 (1.0)
Breast	11311 (2.3)	210 (2.5)*	57 (2.2)	12 (3.0)	30 (2.1)	15 (1.8)
Prostate	3588 (0.7)	105 (1.3)**	30 (1.1)*	5 (1.3)	12 (0.9)	13 (1.6)
GI	2925 (0.6)	96 (1.2)**	34 (1.3)**	6 (1.5)	19 (1.3)	9 (1.1)
Haem	6170 (1.2)	124 (1.5)*	34 (1.3)	5 (1.3)	17 (1.2)	12 (1.5)
**Gastrointestinal**	**55635 (11.5)**	**1737 (20.9)****	**468 (17.9)****	**76 (19.0)**	**254 (18.0)**	**138 (17.0)**
Dyspepsia	37819 (7.7)	1257 (15.1)**	348 (13.3)**	53 (13.3)	189 (13.4)	106 (13.1)
Diverticular dis	5181 (1.0)	224 (2.7)**	54 (2.1)**	6 (1.5)	32 (2.3)	16 (2.0)
IBS	11203 (2.3)	291 (3.5)**	64 (2.4)**	17 (4.3)	35 (2.5)	12 (1.5)
CLD	935 (0.2)	36 (0.4)**	10 (0.4)*	2 (0.5)	10 (0.7)	3 (0.4)
**Mental Health**	**35822 (7.2)**	**1127 (13.6)****	**304 (11.6)****	**54 (13.5)**	**162 (11.5)**	**88 (10.8)**
Depression	27578 (5.6)	901 (10.8)**	233 (8.9)**	42 (10.5)	128 (9.1)	63 (7.8)
Anxiety	8781 (1.8)	245 (2.9)**	69 (2.6)**	13 (3.3)	36 (2.6)	20 (2.5)
Schizophrenia/ bipolar	1918 (0.4)	79 (0.9)**	27 (1.0)**	3 (0.7)	15 (1.1)	9 (1.1)
**Other**						
Painful condition	81733 (16.5)	2259 (27.2)**	655 (25.0)**	115 (28.8)	367 (26.0)	173 (21.3)
Osteoporosis	7700 (1.6)	342 (4.1)**	128 (4.9)**	21 (5.3)	67 (4.8)	40 (4.9)
Connective tissue disease	10642 (2.2)	391 (4.7)**	112 (4.3)**	19 (4.8)	72 (5.1)	21 (2.6)
Compared with control (χ^2): * : p<0.05, ** : p<0.001						

For peer review only - http://bmjopen.bmj.com/site/about/guidelines.xhtml

Morbidity counts (excluding COPD) and counts of prescribed medication are shown in table 2 comparing those with COPD, stratified by severity of airflow obstruction, with those without. Those with COPD had higher numbers of LTCs and more prescribed medications than those without. There was a trend towards more prescribed medications in those with greater severity of airway obstruction. After controlling for age, sex and socioeconomic status, those with self- report COPD were more likely to report ≥4 comorbidities (3.49; 3.28 to 3.71), ≥5 medications (3.85; 3.68 to 4.03), and ≥10 medications (5.72; 5.36 to 6.10) than those without COPD. Results were similar for GOLD COPD and remained statistically significant after adjusting for smoking status, alcohol frequency, BMI and physical activity (appendix 3).

ADR Risk

Counts and percentages of participants taking specific medications are shown in appendix 4. Participants with COPD (self-report and GOLD) were more likely that those without COPD to be prescribed drugs across a range of disease areas, reflecting the range of comorbidities present among those with COPD. The percentages of participants within each category (no COPD, COPD, and COPD with specific comorbidities) taking three or more medications associated with a similar ADR is shown in Figure 2. For each category of ADR a higher proportion of participants with COPD reported taking three or more associated medications than those without COPD. This increased further with comorbidities, with COPD plus cardiovascular comorbidity associated with the highest percentage taking multiple medications with a risk of falls or renal injury, and those with COPD plus mental health conditions showing the highest percentages taking multiple medications with a risk of constipation, CNS depression or bleeding.

After adjusting for age, sex and socioeconomic deprivation, those with self-report COPD remained more likely to be taking three or more medications in each category than those without COPD. These

For peer review only - http://bmjopen.bmj.com/site/about/guidelines.xhtml

findings remained statistically significant after adjusting for smoking status, alcohol frequency, BMI and physical activity (Table 3). Findings were similar for GOLD COPD however, after adjusting for additional potentially confounding variables, results for bleeding risk were not statistically significant in this sensitivity analysis (Table 3).

Table 3. Odds ratios (with 95% CI) for taking 3 of more medications associated with similar ADRs

ADR	Self-report COPD compared with no COPD N=502,640		GOLD COPD compared with no COPD N=496,943	
	Model 1 N=502,013	Model 2 N=487,718	Model 1 N=496,943	Model 2 N=482,378
	OR (95% CI)	OR (95% CI)	OR (95% CI)	OR (95% CI)
Falls	2.27 (2.13 – 2.42) ***	1.83 (1.71-1.96) ***	1.66 (1.47 – 1.87) ***	1.49 (1.30-1.69) ***
Constipation	2.71 (2.54 – 2.89) ***	2.66 (2.39-2.96) ***	2.18 (1.77 – 2.64) ***	1.82 (1.47-2.24) ***
Urinary retention	3.38 (2.94 – 3.87) ***	2.59 (2.22-3.0) ***	1.98 (1.44 – 2.64) ***	1.64 (1.18-2.21) **
CNS depression	3.75 (3.31 – 4.25) ***	2.81 (2.45-3.22) ***	2.29 (1.73 – 2.95) ***	1.87 (1.40-2.43) ***
Bleeding	4.60 (3.35 – 6.19) ***	3.39 (2.40-4.66) ***	2.63 (1.25 – 4.80) **	1.76 (0.75-3.48) §
Renal injury	2.22 (1.86 – 2.62) ***	1.84 (1.53-2.19) ***	1.94 (1.41 – 2.58) ***	1.84 (1.33-2.49) ***

§ : p>0.05 * : p<0.05, ** : p<0.01, *** : p<0.001
Model 1: Adjusted for age, sex and socioeconomic status
Model 2: Adjusted for age, sex, socioeconomic status, smoking, alcohol frequency, body mass index and physical activity

Finally, each category of ADR risk was assessed in a subgroup analysis for each category of comorbidity (cardiovascular, GI, cancer, mental health and painful conditions) comparing those with and without COPD (e.g. participants with cardiovascular disease plus COPD compared with participants with cardiovascular disease alone, etc.). These models were adjusted for age, sex and socioeconomic status only. Within each category of comorbidity, those with self-reported COPD were more likely to be at risk of each ADR than those without COPD (appendix 3). Not all results were statistically significant when using GOLD COPD (Appendix 3).

For peer review only - http://bmjopen.bmj.com/site/about/guidelines.xhtml

DISCUSSION

Summary of main findings

Multimorbidity and polypharmacy in COPD were common among UK Biobank participants. The presence of one or more comorbidity was highly prevalent in those with COPD (85%). More than half reported polypharmacy (five or more medications), and 15% reported 10 or more medications. The prevalence of cardiovascular disease, as well as the degree of polypharmacy, was higher among those with more severe airflow obstruction.

For the first time, our data demonstrates that those with COPD were more likely than those without to be prescribed multiple medications (≥ three) with similar ADRs. Those with COPD plus cardiovascular comorbidity were most likely to be taking multiple medications with a risk of falls and of renal injury, while those with COPD plus comorbid mental health conditions were most likely to be taking medications causing constipation, CNS depression and bleeding. Within each category of comorbidity, those with COPD were more likely to be taking multiple medications with similar ADRs than those without. These associations between patterns of multimorbidity and specific ADR risks have not been described or quantified previously.

Strengths and limitations

Strengths of this study include the large sample size with representation from different areas of the UK. The range of data collected at UK Biobank assessment centres meant it was possible to compare a range of sociodemographic characteristics as well as spirometry data, the latter being unusual for a large community based cohort. It is recognised, however, that UK Biobank participants show some evidence of 'healthy volunteer bias', differing from the UK average on a number of socioeconomic, lifestyle and health-related measures. Specifically they are less socioeconomically deprived, less likely to smoke, to be obese, and have fewer self-reported health conditions.(50) All LTC diagnoses

For peer review only

For peer review only - http://bmjopen.bmj.com/site/about/guidelines.xhtml

as well as medication data were self-reported, with no alternative means of verification. We attempted to minimise this limitation by identifying a subset of those with COPD meeting the GOLD diagnostic criteria and repeating the analyses with this subset. Importantly, spirometry values were also pre-bronchodilator, which is in contravention to guidelines for diagnosing COPD. Additionally, information was not available about the strength of indication for medications and individual susceptibility to risk, which is a limitation when considering the risk of ADRs.

The use of the Scottish Government Polypharmacy Guideline allowed analysis of potential ADR risk by specific common ADRs. The intended purpose of this guideline, however, was not to identify potential risk from a population sample, but rather to identify potential causes of symptoms or complications. The analysis in this study, therefore, serves only as an approximation of potential risk, not an absolute marker of inappropriate polypharmacy. The cross-sectional nature of this analysis also precludes an analysis of actual harm as a result of polypharmacy. Despite these limitations, however, the co-prescription of multiple medications with similar ADRs strongly implies greater potential for harm. The association of such prescribing patterns with COPD, across a range of potential ADRs, is clear from our findings. This analysis is, to the author's knowledge, the first to attempt to quantify this risk for specific ADRs in this way.

Context and implications

The increased prevalence of individual comorbid conditions such as coronary heart disease, hypertension, diabetes, dyspepsia, osteoporosis, cancer, depression and anxiety in those with COPD is similar to the findings from other population based studies of comorbidities in COPD.(5, 11, 51-53) Our finding that cardiovascular disease prevalence increased with increasing severity of COPD is in keeping with the body of literature on cardiovascular comorbidities and COPD, in which high prevalence has been observed in (usually older) cohorts with severe airflow limitation.(5, 21)

For peer review only - http://bmjopen.bmj.com/site/about/guidelines.xhtml

For peer review only

Greater polypharmacy with greater severity of COPD has also been observed previously in older COPD populations,(41, 54) although such analyses have been smaller (n=1859 and 398, respectively) and have not assessed the specific patterns of prescribing in COPD. To the best of our knowledge, no previous studies have assessed the risk of ADRs as a result of polypharmacy in COPD. A recent population-based analysis of prescribing data from 310,000 adults in Scotland showed that over 15 years from 1995 to 2010 the proportion of people with polypharmacy and with potentially serious drug-to-drug interactions increased dramatically.(35) The number of prescribed medications was also associated with increased risk of interactions. Our analysis differs in approach from this analysis, by seeking to identify patterns of prescribing increasing risk of specific adverse events, rather than counting total potential interactions. The strength of our approach lies in highlighting specific patterns of comorbidity in which specific ADRs are more likely. Our findings can therefore be applied to clinical practice, highlighting the importance of recognising comorbidity in COPD and being alert to specific ADRs when prescribing medication.

Our findings indicate that in those with COPD the potential for ADRs as a result of combinations of medications is high, and this appears to be the result of a high prevalence of extra-pulmonary comorbidities. Clinical guidelines for COPD should place greater emphasis on the need for assessment of associated comorbidities and the risk of associated ADRs. While our analysis shows potential areas where ADR risk exists in COPD (e.g. falls with comorbid cardiovascular disease, CNS depression, constipation with comorbid mental health conditions), future research is merited to assess what actual harm could be attributed to such prescribing patterns.

Conclusion

Among UK Biobank participants with COPD there was considerable multimorbidity and polypharmacy. Those with COPD were highly likely to be concurrently prescribed multiple

For peer review only - http://bmjopen.bmj.com/site/about/guidelines.xhtml

medications with similar potential adverse effects. Medications contributing to this risk were largely indicated for the management of the associated comorbidities rather than COPD. Future research should examine the effects on healthcare outcomes of co-prescribing of multiple drugs with similar potential of ADRs. Clinical guidelines for COPD should emphasise the need for assessment of comorbidities and the risk of associated ADRs.

Ethics approval and consent to participate

Participants provided full informed consent to participate in UK Biobank and this study was covered by the generic ethical approval for UK Biobank studies from the NHS National Research Ethics Service (Ref 16/NW/0274).

Availability of data and materials

UK Biobank data is available via *www.ukbiobank.ac.uk*. Syntax for the generation of derived variables and for the analysis used for this study will be submitted to UK Biobank for record.

Competing interests

The authors declare that they have no competing interests

Funding

This study was funded by a CSO Catalyst Grant 173804. BDJ was funded by a NHS Research for Scotland career research fellowship.

For peer review only - http://bmjopen.bmj.com/site/about/guidelines.xhtml

Author contributions

All authors (PH, BN, BJ, RM, DL, KG and FM) were involved in the conceptualisation and design of the project and interpretation of results. PH carried out the analysis with support from BJ, RM and BN. DL provided statistical support. All authors had access to the data. PH wrote the first draft of the paper and all authors commented on subsequent drafts. All authors approved the final draft for publication. FM is guarantor.

Acknowledgements

This research has been conducted using the UK Biobank Resource, approved project number 14151.

References

1. Anecchino C, Rossi E, Fanizza C, De Rosa M, Tognoni G, Romero M. Prevalence of chronic obstructive pulmonary disease and pattern of comorbidities in a general population. International journal of chronic obstructive pulmonary disease. 2007;2(4):567-74.
2. Fumagalli G, Fabiani F, Forte S, Napolitano M, Marinelli P, Palange P, et al. INDACO project: a pilot study on incidence of comorbidities in COPD patients referred to pneumology units. Multidisciplinary Respiratory Medicine. 2013;8.
3. Putcha N, Puhan MA, Hansel NN, Drummond MB, Boyd CM. Impact of co-morbidities on self-rated health in self-reported COPD: An analysis of NHANES 2001-2008. Copd-Journal of Chronic Obstructive Pulmonary Disease. 2013;10(3):324-32.
4. Garcia-Olmos L, Alberquilla A, Ayala V, Garcia-Sagredo P, Morales L, Carmona M, et al. Comorbidity in patients with chronic obstructive pulmonary disease in family practice: a cross sectional study. BMC family practice. 2013;14.
5. Chen W, Thomas J, Sadatsafavi M, FitzGerald JM. Risk of cardiovascular comorbidity in patients with chronic obstructive pulmonary disease: a systematic review and meta-analysis. Lancet Respir Med. 2015;3(8):631-9.
6. Rodriguez DA, Garcia-Aymerich J, Valera JL, Sauleda J, Togores B, Galdiz JB, et al. Determinants of exercise capacity in obese and non-obese COPD patients. Respiratory Medicine. 2014;108(5):745-51.
7. Al-shair K, Dockry R, Mallia-Milanes B, Kolsum U, Singh D, Vestbo J. Depression and its relationship with poor exercise capacity, BODE index and muscle wasting in COPD. Respiratory Medicine. 2009;103(10):1572-9.

For peer review only

8. Di Marco F, Verga M, Reggente M, Casanova FM, Santus P, Blasi F, et al. Anxiety and depression in COPD patients: The roles of gender and disease severity. Respiratory Medicine. 2006;100(10):1767-74.

9. Qian J, Simoni-Wastila L, Rattinger GB, Lehmann S, Langenberg P, Zuckerman IH, et al. Associations of depression diagnosis and antidepressant treatment with mortality among young and disabled Medicare beneficiaries with COPD. General Hospital Psychiatry. 2013;35(6):612-8.

10. Atlantis E, Fahey P, Cochrane B, Smith S. Bidirectional Associations Between Clinically Relevant Depression or Anxiety and COPD A Systematic Review and Meta-analysis. Chest. 2013;144(3):766-77.

11. Bor S, Kitapcioglu G, Solak ZA, Ertilav M, Erdinc M. Prevalence of gastroesophageal reflux disease in patients with asthma and chronic obstructive pulmonary disease. Journal of Gastroenterology and Hepatology. 2010;25(2):309-13.

12. Garcia Rodriguez LA, Ruigomez A, Martin-Merino E, Johansson S, Wallander M-A. Relationship Between Gastroesophageal Reflux Disease and COPD in UK Primary Care. Chest. 2008;134(6):1223-30.

13. Kim J, Lee JH, Kim Y, Kim K, Oh Y-M, Yoo KH, et al. Association between chronic obstructive pulmonary disease and gastroesophageal reflux disease: a national cross-sectional cohort study. Bmc Pulmonary Medicine. 2013;13.

14. Ferguson GT, Calverley PMA, Anderson JA, Jenkins CR, Jones PW, Willits LR, et al. Prevalence and Progression of Osteoporosis in Patients With COPD Results From the Towards a Revolution in COPD Health Study. Chest. 2009;136(6):1456-65.

15. Jorgensen NR, Schwarz P, Holme I, Henriksen BM, Petersen LJ, Backer V. The prevalence of osteoporosis in patients with chronic obstructive pulmonary disease - A cross sectional study. Respiratory Medicine. 2007;101(1):177-85.

16. Ogura-Tomomatsu H, Asano K, Tomomatsu K, Miyata J, Ohmori N, Kodama M, et al. Predictors of Osteoporosis and Vertebral Fractures in Patients Presenting with Moderate-to-Severe Chronic Obstructive Lung Disease. Copd-Journal of Chronic Obstructive Pulmonary Disease. 2012;9(4):332-7.

17. Young RP, Hopkins RJ, Christmas T, Black PN, Metcalf P, Gamble GD. COPD prevalence is increased in lung cancer, independent of age, sex and smoking history. European Respiratory Journal. 2009;34(2):380-6.

18. Wilson DO, Weissfeld JL, Balkan A, Schragin JG, Fuhrman CR, Fisher SN, et al. Association of radiographic emphysema and airflow obstruction with lung cancer. American Journal of Respiratory and Critical Care Medicine. 2008;178(7):738-44.

19. Garcia-Rio F, Soriano JB, Miravitlles M, Munoz L, Duran-Tauleria E, Sanchez G, et al. Impact of Obesity on the Clinical Profile of a Population-Based Sample with Chronic Obstructive Pulmonary Disease. Plos One. 2014;9(8).

20. Cecere LM, Littman AJ, Slatore CG, Udris EM, Bryson CL, Boyko EJ, et al. Obesity and COPD: Associated Symptoms, Health-related Quality of Life, and Medication Use. Copd-Journal of Chronic Obstructive Pulmonary Disease. 2011;8(4):275-84.

21. Mannino DM, Thorn D, Swensen A, Holguin F. Prevalence and outcomes of diabetes, hypertension and cardiovascular disease in COPD. European Respiratory Journal. 2008;32(4):962-9.

22. Curkendall SM, DeLuise C, Jones JK, Lanes S, Stang MR, Goehring E, et al. Cardiovascular disease in patients with chronic obstructive pulmonary disease, Saskatchewan Canada cardiovascular disease in COPD patients. Annals of Epidemiology. 2006;16(1):63-70.

23. McGarvey LP, John M, Anderson JA, Zvarich M, Wise RA. Ascertainment of cause-specific mortality in COPD: operations of the TORCH Clinical Endpoint Committee. Thorax. 2007;62(5):411-5.

24. Zielinski J, MacNee W, Wedzicha J, Ambrosino N, Braghiroli A, Dolensky J, et al. Causes of death in patients with COPD and chronic respiratory failure. Monaldi archives for chest disease = Archivio Monaldi per le malattie del torace. 1997;52(1):43-7.

For peer review only - http://bmjopen.bmj.com/site/about/guidelines.xhtml

25. Mannino DM, Doherty DE, Buist AS. Global Initiative on Obstructive Lung Disease (GOLD) classification of lung disease and mortality: findings from the Atherosclerosis Risk in Communities (ARIC) study. Respiratory Medicine. 2006;100(1):115-22.
26. Campo G, Guastaroba P, Marzocchi A, Santarelli A, Varani E, Vignali L, et al. Impact of COPD on Long-term Outcome After ST-Segment Elevation Myocardial Infarction Receiving Primary Percutaneous Coronary Intervention. Chest. 2013;144(3):750-7.
27. Konecny T, Somers K, Orban M, Koskino Y, Lennon RJ, Scanlon PD, et al. Interactions Between COPD and Outcomes After Percutaneous Coronary Intervention. Chest. 2010;138(3):621-7.
28. Sidney S, Sorel M, Quesenberry CP, DeLuise C, Lanes S, Eisner MD. COPD and incident cardiovascular disease hospitalizations and mortality: Kaiser permanente medical care program. Chest. 2005;128(4):2068-75.
29. Sakae TM, Menezes Pizzichini MM, Zimermann Teixeira PJ, da Silva RM, Trevisol DJ, Pizzichini E. Exacerbations of COPD and symptoms of gastroesophageal reflux: a systematic review and meta-analysis. Jornal Brasileiro De Pneumologia. 2013;39(3):259-71.
30. Hattiholi J, Gaude GS. Prevalence and correlates of osteoporosis in chronic obstructive pulmonary disease patients in India. Lung India : official organ of Indian Chest Society. 2014;31(3):221-7.
31. Miller J, Edwards LD, Agusti A, Bakke P, Calverley PMA, Celli B, et al. Comorbidity, systemic inflammation and outcomes in the ECLIPSE cohort. Respiratory Medicine. 2013;107(9):1376-84.
32. Divo M, Cote C, de Torres JP, Casanova C, Marin JM, Pinto-Plata V, et al. Comorbidities and Risk of Mortality in Patients with Chronic Obstructive Pulmonary Disease. American Journal of Respiratory and Critical Care Medicine. 2012;186(2):155-61.
33. Budweiser S, Harlacher M, Pfeifer M. Co-morbidities and hyperinflation are independent risk factors of all-cause mortality in very severe COPD. COPD. 2014;11(4):388-400.
34. Corsonello A, Pedone C, Corica F. Polypharmacy in elderly patients at discharge from the acute care hospital. Therapeutics and Clinical Risk Management. 2007;2007(3):1.
35. Guthrie B, Makubate B, Hernandez-Santiago V, Dreischulte T. The rising tide of polypharmacy and drug-drug interactions: population database analysis 1995-2010. BMC Medicine. 2015;13:74.
36. Bourgeois FT, Shannon MW, Valim C, Mandl KD. Adverse drug events in the outpatient setting: an 11-year national analysis. Pharmacoepidemiol Drug Saf. 2010;19(9):901-10.
37. Johnell K, Klarin I. The relationship between number of drugs and potential drug-drug interactions in the elderly: a study of over 600,000 elderly patients from the Swedish Prescribed Drug Register. Drug Saf. 2007;30(10):911-8.
38. Pirmohamed M, James S, Meakin S, Green C, Scott AK, Walley TJ, et al. Adverse drug reactions as cause of admission to hospital: prospective analysis of 18 820 patients. BMJ. 2004;329(7456):15-9.
39. Howard RL, Avery AJ, Slavenburg S, Royal S, Pipe G, Lucassen P, et al. Which drugs cause preventable admissions to hospital? A systematic review. British Journal of Clinical Pharmacology. 2007;63(2):136-47.
40. Nobili A, Marengoni A, Tettamanti M, Salerno F, Pasina L, Franchi C, et al. Association between clusters of diseases and polypharmacy in hospitalized elderly patients: results from the REPOSI study. European Journal of Internal Medicine. 2011;22(6):597-602.
41. Franssen FM, Spruit MA, Wouters EF. Determinants of polypharmacy and compliance with GOLD guidelines in patients with chronic obstructive pulmonary disease. Int J Chron Obstruct Pulmon Dis. 2011;6:493-501.
42. Patel A. Extrapulmonary Polypharmacy and Cardiovascular Medications in COPD. Thorax. 2009;64(Suppl IV):A5-A74.
43. Miller MR, Hankinson J, Brusasco V, Burgos F, Casaburi R, Coates A, et al. Standardisation of spirometry. European Respiratory Journal. 2005;26(2):319-38.

For peer review only - http://bmjopen.bmj.com/site/about/guidelines.xhtml

44. Qaseem A, Wilt TJ, Weinberger SE, Hanania NA, Criner G, van der Molen T, et al. Diagnosis and management of stable chronic obstructive pulmonary disease: a clinical practice guideline update from the American College of Physicians, American College of Chest Physicians, American Thoracic Society, and European Respiratory Society. Annals of internal medicine. 2011;155(3):179-91.

45. Global Strategy for the Diagnosis, Management and Prevention of COPD, Global Initiative for Chronic Obstructive Lung Disease (GOLD) 2017. [Available from: http://goldcopd.org]

46. Hankinson JL, Odencrantz JR, Fedan KB. Spirometric reference values from a sample of the general U.S. population. American Journal of Respiratory & Critical Care Medicine. 1999;159(1):179-87.

47. Barnett K, Mercer SW, Norbury M, Watt G, Wyke S, Guthrie B. Epidemiology of Multimorbidity and Implications for Health Care, Research, and Medical Education: a Cross-Sectional Study. Lancet. 2012;380(9836):37-43.

48. Nicholl BI, Mackay D, Cullen B, Martin DJ, Ul-Haq Z, Mair FS, et al. Chronic multisite pain in major depression and bipolar disorder: cross-sectional study of 149,611 participants in UK Biobank. BMC Psychiatry. 2014;14:350.

49. Scottish Government Model of Care Polypharmacy Working Group. Polypharmacy Guidance (2nd edition) March 2015. Scottish Government. [Available from: http://www.sign.ac.uk/pdf/polypharmacy_guidance.pdf]

50. Fry A, Littlejohns TJ, Sudlow C, Doherty N, Allen NE. The representativeness of the UK Biobank cohort on a range of sociodemographic, physical, lifestyle and health-related characteristics. . Journal of epidemiology and community health. 2016;70(Suppl 1):A26-A.

51. Putcha N, Han MK, Martinez CH, Foreman MG, Anzueto AR, Casaburi R, et al. Comorbidities of COPD have a major impact on clinical outcomes, particularly in African Americans. Chronic obstructive pulmonary diseases (Miami, Fla). 2014;1(1):105-14.

52. Bhattacharyya P, Paul R, Ghosh M, Dey R, Dey R, Barooah N, et al. Prevalence of osteoporosis and osteopenia in advanced chronic obstructive pulmonary disease patients. Lung India : official organ of Indian Chest Society. 2011;28(3):184-6.

53. de Torres JP, Bastarrika G, Wisnivesky JP, Alcaide AB, Campo A, Seijo LM, et al. Assessing the relationship between lung cancer risk and emphysema detected on low-dose CT of the chest. Chest. 2007;132(6):1932-8.

54. Diez-Manglano J, Barquero-Romero J, Mena PA, Recio-Iglesias J, Cabrera-Aguilar J, Lopez-Garcia F, et al. Polypharmacy in patients hospitalised for acute exacerbation of COPD. European Respiratory Journal. 2014;44(3):791-4.

For peer review only

For peer review only - http://bmjopen.bmj.com/site/about/guidelines.xhtml

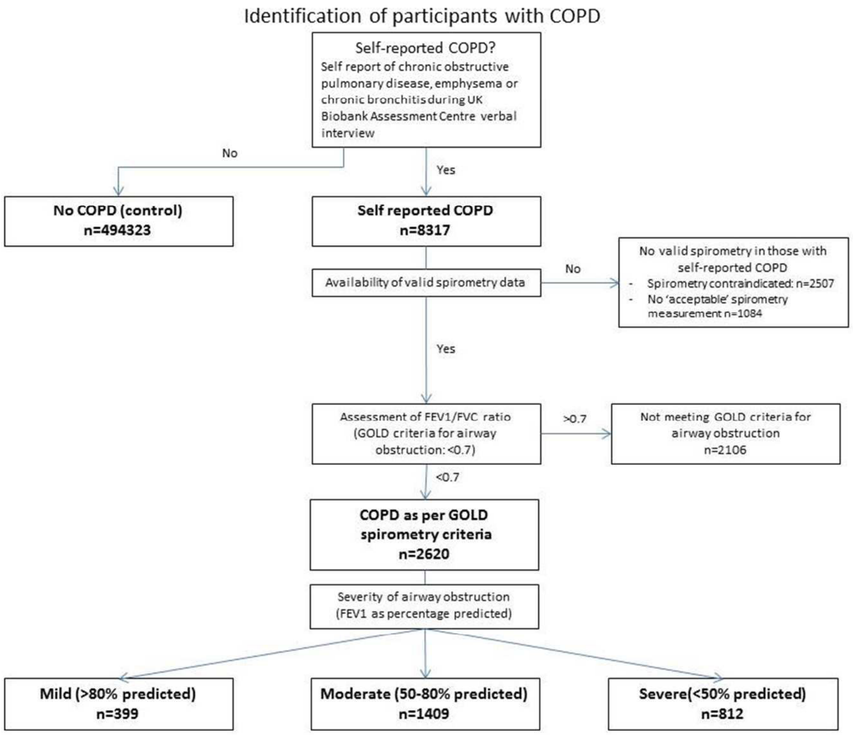

Identification of participants with self-report COPD and GOLD COPD

254x190mm (96 x 96 DPI)

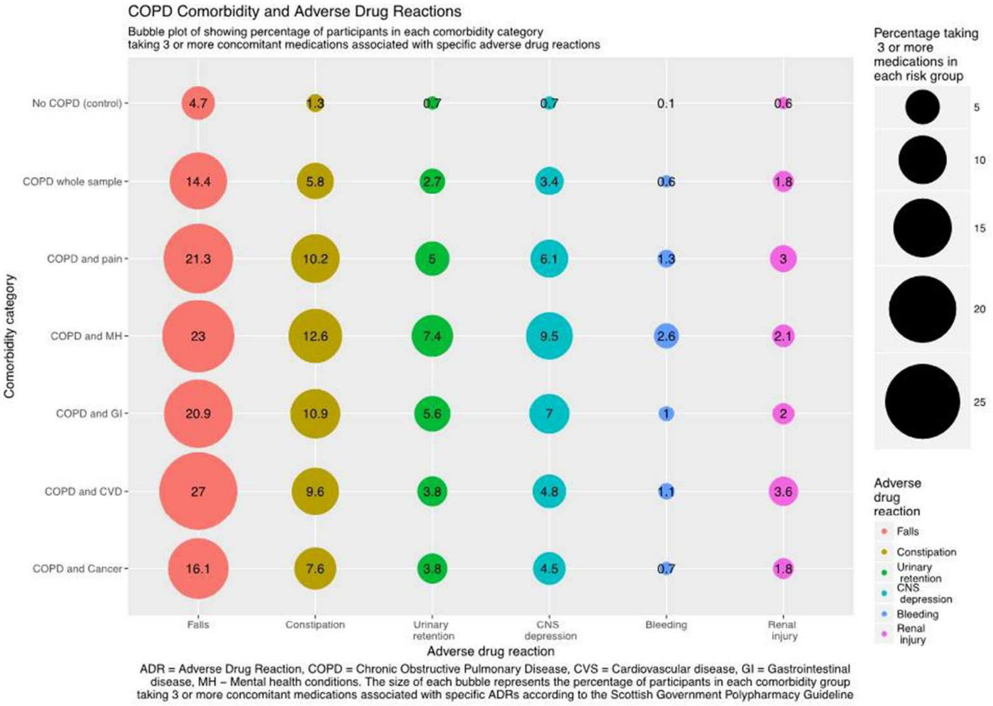

Bubble plot showing percentage or participants in each comorbidity category taking 3 or more concomitant medications associated with specific adverse drug reactions.

254x190mm (96 x 96 DPI)

w only

**Comorbidity category (used in analysis)**	**Conditions included (as reported in table 2)**	**Self-reported conditions comprising this condition (UK Biobank variables used to identify self-reported conditions)**
Cardiovascular conditions	Hypertension	Hypertension<br>Essential hypertension
	Coronary heart disease	Heart attack/MI<br>Angina
	Diabetes	Diabetic nephropathy<br>Diabetic neuropathy/ulcers<br>Diabetes<br>Type 1 diabetes<br>Type 2 diabetes<br>Diabetic eye disease
	Stroke/TIA	Stroke<br>TIA<br>Subarachnoid haemorrhage<br>Brain haemorrhage<br>Ischaemic stroke
	Atrial fibrillation	Atrial fibrillation
	Heart failure	Cardiomyopathy<br>Hypertrophic cardiomyopathy<br>Heart failure/pulmonary oedema
	Peripheral vascular disease	Peripheral vascular disease<br>Leg claudication/intermittent claudication
Respiratory	COPD	COPD/Chronic obstructive pulmonary disease<br>Emphysema/Chronic bronchitis<br>Emphysema
	Asthma	Asthma
	PE/DVT	Deep vein thrombosis<br>Pulmonary emolism
	Bronchiectasis	Bronchiectasis
	Pulmonary fibrosis	Pulmonary fibrosis
Cancer	Cancer	"yes"/"no" to "have you ever had cancer?"
Gastrointestinal	Dyspepsia	Gastro-oesophageal reflux (GORD)<br>Oesophagitis/Barrett's oesophagus<br>Gastric stomach ulcers<br>Gastric erosions/gastritis<br>Duodenal ulcer<br>Dyspepsia/indigestion<br>Hiatus hernia<br>Helicobacter pylori
	Diverticular disease	Diverticular disease/diverticulitis

For peer review only

	Irritable bowel syndrome	Irritable bowel syndrome
	Chronic liver disease	Oesophageal varices<br>Non infective hepatitis<br>Liver failure/cirrhosis<br>Primary biliary cirrhosis
	Inflammatory bowel disease	Inflammatory bowel disease<br>Crohn's disease<br>Ulcerative colitis
	Constipation	Constipation
	Viral hepatitis	Hepatitis B<br>Hepatitis C<br>Hepatitis D
Mental Health	Depression	Depression<br>Postnatal depression
	Anxiety	Anxiety/panic attacks<br>Nervous breakdown<br>Post-traumatic stress disorder<br>Obsessive compulsive disorder<br>Stress<br>Insomnia<br>Psychological/psychiatric problem
	Schizophrenia	Scizophrenia
	Bipolar	Mania<br>Bipolar disorder<br>Manic depression
Painful conditions	Painful conditions	Back pain<br>Joint pain<br>Headaches (not migraine)<br>Sciatica<br>Plantar fasciitis<br>Carpal tunnel syndrome<br>Fibromyalgia<br>Arthritis<br>Shingles<br>Disc problem<br>Prolapsed disc/slipped disc<br>Spine arthritis/spondylitis<br>Ankylosing spondylitis<br>Back problem<br>Osteoarthritis<br>Gout<br>Cervical spondylosis<br>Trigeminal neuralgia<br>Disc degeneration<br>Trapped nerve/compressed nerve
Other	Osteoporosis	Osteoporosis
	Connective tissue disease	Myositis/myopathy<br>Systemic lupus erythematosus/SLE

For peer review only

		Connective tissue disorder<br>Sjogren's syndrome.sicca syndrome<br>Dermatopolymyositis<br>Scloeroderma/systemic sclerosis<br>Rheumatoid arthritis<br>Psoriatic arthropathy<br>Dermatomyositis<br>Polymyositis<br>Polymyalgia rheumatica

For peer review only

Drugs with cumulative risk of Adverse Drug Reactions*	
Adverse Drug Reaction	Contributing drug classes (participants taking 3 or more considered 'at risk' for the purposes of analysis)
Falls	H2-receptor blockers<br>Loperamide<br>Prochlorperazine<br>Metoclopramide<br>ACE-inhibitor/Angiotensin receptor blocker<br>Thiazide diuretic<br>Loop diuretic<br>Amiloride/triamterene<br>Spironolactone<br>Beta-blocker<br>Calcium-channel blocker<br>Nitrates or nicorandil<br>Digoxin<br>Oral steroids<br>Opiates<br>Benzodiazepines<br>Sedative antihistamines<br>H1-receptor blockers<br>Antipsychotics<br>Tricyclic antidepressants<br>Sulfonylureas/gliptins/glinides<br>Pioglitazone<br>Urinary antispasmodics<br>Dosulepin<br>Alpha-blockers
Constipation	H2-receptor blockers<br>Laxatives<br>Loperamide<br>Prochlorperazine<br>Thiazide diuretics<br>Loop diuretics<br>Calcium-channel blockers<br>Opiates<br>Sedative antihistamines<br>H1-receptor blockers<br>Antipsychotics<br>Tricyclic antidepressants<br>Urinary antispasmodics<br>Dosulepin
Urinary retention	H2-receptor blockers<br>Loperamide<br>Prochlorperazine<br>Opiates<br>Sedative antihistamines<br>H1-receptor blockers<br>Antipsychotics<br>Tricyclic antidepressants

For peer review only

	Urinary antispasmodics<br>Dosulepin
CNS depression	H2-receptor blockers<br>Loperamide<br>Prochlorperazine<br>Oral steroids<br>Opiates<br>Benzodiazepines<br>Sedative antihistamines<br>H1-receptor blockers<br>Antipsychotics<br>Tricyclic antidepressants<br>Urinary antispasmodics<br>Dosulepin
Bleeding	Aspirin<br>Clopidogrel<br>Other antiplatelets<br>Oral steroids<br>SSRIs and related drugs<br>Non-steroidal anti-inflammatory drugs<br>Warfarin
Renal injury	ACE-inhibitor/angiotensin receptor blockers<br>Thiazide diuretic<br>Loop diuretic<br>Amiloride/triamterene<br>Spironolactone<br>Antibiotics/antifungals<br>Non-steroidal anti-inflammatory drugs
Adapted from *Scottish Government Model of Care Polypharmacy Working Group. Polypharmacy Guidance (2nd edition) March 2015. Scottish Government.*	

For peer review only

**Table S1. Odds ratios (with 95% CI) for the presence of categories of comorbidity**				
Comorbidity category	Self-report COPD compared with no COPD N=502,640		GOLD COPD compared with no COPD N=496,943	
	Model 1 N=502,013	Model 2 N=487,718	Model 1 N=496,324	Model 2 N=482,378
	OR (95% CI)	OR (95% CI)	OR (95% CI)	OR (95% CI)
Cardiovascular disease	1.45 (1.39-1.52) ***	1.28 (1.22-1.34) ***	1.11 (1.02-1.20) *	1.08 (0.99-1.18) §
Cancer	1.29 (1.20-1.39) ***	1.22 (1.13-1.31) ***	1.12 (0.99-1.27) §	1.06 (0.92-1.19) §
Gastrointestinal disease	1.76 (1.67-1.86) ***	1.56 (1.48-1.65) ***	1.4 (1.26-1.54) ***	1.24 (1.12-1.38) ***
Mental health	2.02 (1.89-2.15) ***	1.62 (1.51-1.73) ***	1.75 (1.54-1.97) ***	1.40 (1.22-1.58) ***
Painful conditions	1.54 (1.46-1.62) ***	1.35 (1.28-1.42) ***	1.31 (1.19-1.43) ***	1.16 (1.06-1.28) **

§ : p>0.05 * : p<0.05, ** : p<0.01, *** : p<0.001
Model 1: Adjusted for age, sex and socioeconomic status
Model 2: Adjusted for age, sex, socioeconomic status, smoking, alcohol frequency, body mass index and physical activity

**Table S2. Odds ratios (with 95% CI) for the presence of multimorbidity or polypharmacy**				
Outcome	Self-report COPD compared with no COPD N=502,640		GOLD COPD compared with no COPD N=496,943	
	Model 1 N=502,013	Model 2 N=487,718	Model 1 N=496,324	Model 2 N=482,378
	OR (95% CI)	OR (95% CI)	OR (95% CI)	OR (95% CI)
Multimorbidity (≥4 conditions)	3.49 (3.28-3.70) ***	2.79 (2.61-2.98) ***	2.34 (2.10-2.63) ***	1.99 (1.75-2.25) ***
Polypharmacy (≥5 medications)	3.85 (3.68-4.03) ***	3.30 (3.15-3.46) ***	3.47 (3.20-3.75) ***	3.20 (2.95-3.48) ***
Polypharmacy (≥10 medications	5.72 (5.36-6.10) ***	4.42 (4.11-4.75) ***	4.20 (3.72-4.73) ***	3.56 (3.12-4.05) ***

§ : p>0.05 * : p<0.05, ** : p<0.01, *** : p<0.001
Model 1: Adjusted for age, sex and socioeconomic status
Model 2: Adjusted for age, sex, socioeconomic status, smoking, alcohol frequency, body mass index and physical activity

**Table S3. Odds ratios (with 95% CI) for taking 3 of more medications associated with similar ADRs**				
ADR	Self-report COPD compared with no COPD N=502,640		GOLD COPD compared with no COPD N=496,943	
	Model 1 N=502,013	Model 2 N=487,718	Model 1 N=496,943	Model 2 N=482,378
	OR (95% CI)	OR (95% CI)	OR (95% CI)	OR (95% CI)
Falls	2.27 (2.13 – 2.42) ***	1.83 (1.71-1.96) ***	1.66 (1.47 – 1.87) ***	1.49 (1.30-1.69) ***
Constipation	2.71 (2.54 – 2.89) ***	2.66 (2.39-2.96) ***	2.18 (1.77 – 2.64) ***	1.82 (1.47-2.24) ***
Urinary retention	3.38 (2.94 – 3.87) ***	2.59 (2.22-3.0) ***	1.98 (1.44 – 2.64) ***	1.64 (1.18-2.21) **
CNS Depression	3.75 (3.31 – 4.25) ***	2.81 (2.45-3.22) ***	2.29 (1.73 – 2.95) ***	1.87 (1.40-2.43) ***
Bleeding	4.60 (3.35 – 6.19) ***	3.39 (2.40-4.66) ***	2.63 (1.25 – 4.80) **	1.76 (0.75-3.48) §
Renal injury	2.22 (1.86 – 2.62) ***	1.84 (1.53-2.19) ***	1.94 (1.41 – 2.58) ***	1.84 (1.33-2.49) ***

§ : p>0.05 * : p<0.05, ** : p<0.01, *** : p<0.001
Model 1: Adjusted for age, sex and socioeconomic status
Model 2: Adjusted for age, sex, socioeconomic status, smoking, alcohol frequency, body mass index and physical activity

For peer review only

Subgroup analyses – comparing COPD with no COPD among participants with specific categories of comorbidity

Table S4. Odds ratios (with 95% CI) for taking 3 of more medications associated with similar ADRs in participants with cardiovascular disease (CVD)

ADR	Self-report COPD plus CVD compared with CVD alone (no COPD) N=156,848	GOLD COPD plus CVD compared with CVD alone (no COPD) N=154,047
	Model 1 N=156,667	Model 1 N=153,852
	OR (95% CI)	OR (95% CI)
Falls	1.92 (1.79-2.07) ***	1.59 (1.39-1.82) ***
Constipation	2.89 (2.58-3.23) ***	2.06 (1.63-2.57) ***
Urinary retention	2.78 (2.33-3.28) ***	1.92 (1.30-2.72) ***
CNS Depression	3.17 (2.71-3.69) ***	2.17 (1.54-2.97) ***
Bleeding	4.00 (2.85-5.48) ***	2.26 (0.96-4.44) *
Renal injury	1.90 (1.59-2.25) ***	1.82 (1.31-2.45) ***

§ : p>0.05 * : p<0.05, ** : p<0.01, *** : p<0.001
Model 1: Adjusted for age, sex and socioeconomic status

Table S5. Odds ratios (with 95% CI) for taking 3 of more medications associated with similar ADRs in participants with cancer

ADR	Self-report COPD plus cancer compared with cancer alone (no COPD) N=38,623	GOLD COPD plus cancer compared with cancer alone (no COPD) N=37,958
	Model 1 N=38,575	Model 1 N= 37,912
	OR (95% CI)	OR (95% CI)
Falls	2.35 (1.95-2.81) ***	1.49 (1.00-2.13) *
Constipation	3.55 (2.73-4.56) ***	2.21 (1.22-3.68) **
Urinary retention	3.65 (2.52-5.13) ***	1.99 (0.78-4.14) §
CNS Depression	3.74 (2.66-5.14) ***	2.04 (0.86-4.04) §
Bleeding	4.69 (1.91-9.86) ***	2.20 (0.12-10.23) §
Renal injury	2.0 (1.17-3.20) **	2.26 (0.89-4.71) §

§ : p>0.05 * : p<0.05, ** : p<0.01, *** : p<0.001
Model 1: Adjusted for age, sex and socioeconomic status

Table S6. Odds ratios (with 95% CI) for taking 3 of more medications associated with similar ADRs in participants with gastrointestinal disease (GI)

ADR	Self-report COPD plus GI compared with GI alone (no COPD) N=58372	GOLD COPD plus GI compared with GI alone (no COPD) N=57103
	Model 1 N=58,299	Model 1 N=57,031
	OR (95% CI)	OR (95% CI)
Falls	2.18 (1.92-2.46) ***	1.46 (1.13-1.87) **
Constipation	2.70 (2.29-3.16) ***	1.58 (1.08-2.24) *
Urinary retention	2.64 (2.12-3.26) ***	1.46 (0.83-2.37) §
CNS Depression	3.02 (2.47-3.66) ***	1.50 (0.88-2.37) §
Bleeding	3.88 (2.27-6.25) ***	3.18 (0.97-7.63) §
Renal injury	1.99 (1.37-2.80) ***	1.22 (0.48-2.51) §

§ : p>0.05 * : p<0.05, ** : p<0.01, *** : p<0.001
Model 1: Adjusted for age, sex and socioeconomic status

For peer review only

**Table S7. Odds ratios (with 95% CI) for taking 3 of more medications associated with similar ADRs in participants with mental health conditions (MH)**		
ADR	Self-report COPD plus MH compared with MH alone (no COPD)<br>N=36,949	GOLD COPD plus MH compared with MH alone (no COPD)<br>N=36126
	Model 1<br>N=36,885	Model 1<br>N=36,065
	OR (95% CI)	OR (95% CI)
Falls	2.21 (1.90-2.56) ***	1.35 (0.99-1.82) §
Constipation	2.33 (1.93-2.81) ***	1.62 (1.08-2.34) *
Urinary retention	2.17 (1.71-2.74) ***	1.42 (0.82-2.29) §
CNS Depression	2.53 (2.04-3.12) ***	1.66 (1.03-2.54) *
Bleeding	2.86 (1.77-4.17) ***	1.94 (0.76-4.05) §
Renal injury	1.86 (1.19-2.79) **	1.27 (0.45-2.80) §
§ : p>0.05 * : p<0.05, ** : p<0.01, *** : p<0.001<br>Model 1: Adjusted for age, sex and socioeconomic status		

**Table S8. Odds ratios (with 95% CI) for taking 3 of more medications associated with similar ADRs in participants with painful conditions**		
ADR	Self-report COPD plus painful conditions compared with painful conditions alone (no COPD)<br>N=83,992	GOLD COPD plus painful conditions compared with painful conditions alone (no COPD)<br>N=82,388
	Model 1<br>N=83,895	Model 1<br>N=82,294
	OR (95% CI)	OR (95% CI)
Falls	1.99 (1.79-2.21) ***	1.52 (1.23-1.85) ***
Constipation	2.57 (2.22-2.96) ***	1.63 (1.19-2.20) **
Urinary retention	2.47 (2.01-3.00) ***	1.19 (0.69-1.89) §
CNS Depression	2.77 (2.30-3.31) ***	1.48 (0.93-2.22) §
Bleeding	4.23 (2.80-6.14) ***	2.36 (0.84-5.20) §
Renal injury	1.67 (1.29-2.13) ***	1.46 (0.87-2.27) §
§ : p>0.05 * : p<0.05, ** : p<0.01, *** : p<0.001<br>Model 1: Adjusted for age, sex and socioeconomic status		

For peer review only

STROBE 2007 (v4) Statement—Checklist of items that should be included in reports of *cross-sectional studies*

Section/Topic	Item #	Recommendation	Reported on page #
**Title and abstract**	1	(*a*) Indicate the study's design with a commonly used term in the title or the abstract	2
		(*b*) Provide in the abstract an informative and balanced summary of what was done and what was found	2,3
**Introduction**			
Background/rationale	2	Explain the scientific background and rationale for the investigation being reported	4
Objectives	3	State specific objectives, including any prespecified hypotheses	5
**Methods**			
Study design	4	Present key elements of study design early in the paper	5,6,9
Setting	5	Describe the setting, locations, and relevant dates, including periods of recruitment, exposure, follow-up, and data collection	6,7
Participants	6	(*a*) Give the eligibility criteria, and the sources and methods of selection of participants	7
Variables	7	Clearly define all outcomes, exposures, predictors, potential confounders, and effect modifiers. Give diagnostic criteria, if applicable	6-8
Data sources/ measurement	8*	For each variable of interest, give sources of data and details of methods of assessment (measurement). Describe comparability of assessment methods if there is more than one group	6-8
Bias	9	Describe any efforts to address potential sources of bias	*14,15*
Study size	10	Explain how the study size was arrived at	7
Quantitative variables	11	Explain how quantitative variables were handled in the analyses. If applicable, describe which groupings were chosen and why	6,7,8,9
Statistical methods	12	(*a*) Describe all statistical methods, including those used to control for confounding	8-10
		(*b*) Describe any methods used to examine subgroups and interactions	9,10
		(*c*) Explain how missing data were addressed	10
		(*d*) If applicable, describe analytical methods taking account of sampling strategy	n/a
		(*e*) Describe any sensitivity analyses	9,10
**Results**			

For peer review only

Participants	13*	(a) Report numbers of individuals at each stage of study—eg numbers potentially eligible, examined for eligibility, confirmed eligible, included in the study, completing follow-up, and analysed	11, figure 1
		(b) Give reasons for non-participation at each stage	11, Figure 1
		(c) Consider use of a flow diagram	Figure 1
Descriptive data	14*	(a) Give characteristics of study participants (eg demographic, clinical, social) and information on exposures and potential confounders	Table 1
		(b) Indicate number of participants with missing data for each variable of interest	Table 1
Outcome data	15*	Report numbers of outcome events or summary measures	11-13, figure 2, appendix 3
Main results	16	(*a*) Give unadjusted estimates and, if applicable, confounder-adjusted estimates and their precision (eg, 95% confidence interval). Make clear which confounders were adjusted for and why they were included	11-13, figure 2, appendix 3
		(*b*) Report category boundaries when continuous variables were categorized	12,13
		(*c*) If relevant, consider translating estimates of relative risk into absolute risk for a meaningful time period	n/a
Other analyses	17	Report other analyses done—eg analyses of subgroups and interactions, and sensitivity analyses	12,13, Appendix 3,
**Discussion**			
Key results	18	Summarise key results with reference to study objectives	14
Limitations	19	Discuss limitations of the study, taking into account sources of potential bias or imprecision. Discuss both direction and magnitude of any potential bias	14,15
Interpretation	20	Give a cautious overall interpretation of results considering objectives, limitations, multiplicity of analyses, results from similar studies, and other relevant evidence	15,16
Generalisability	21	Discuss the generalisability (external validity) of the study results	15,16,17
**Other information**			
Funding	22	Give the source of funding and the role of the funders for the present study and, if applicable, for the original study on which the present article is based	17,18

*Give information separately for cases and controls in case-control studies and, if applicable, for exposed and unexposed groups in cohort and cross-sectional studies.

Note: An Explanation and Elaboration article discusses each checklist item and gives methodological background and published examples of transparent reporting. The STROBE checklist is best used in conjunction with this article (freely available on the Web sites of PLoS Medicine at http://www.plosmedicine.org/, Annals of Internal Medicine at http://www.annals.org/, and Epidemiology at http://www.epidem.com/). Information on the STROBE Initiative is available at www.strobe-statement.org.

For peer review only

BMJ Open

Examining Patterns of Multimorbidity, Polypharmacy and Risk of Adverse Drug Reactions in Chronic Obstructive Pulmonary Disease: A Cross-Sectional UK Biobank Study

Journal:	*BMJ Open*
Manuscript ID	bmjopen-2017-018404.R1
Article Type:	Research
Date Submitted by the Author:	10-Oct-2017
Complete List of Authors:	Hanlon, Peter; University of Glasgow Institute of Health and Wellbeing<br>Nicholl, Barbara; University of Glasgow, General Practice and Primary Care<br>Jani, Bhautesh; University of Glasgow<br>McQueenie, Ross; University of Glasgow, GPPC, School of Medicine, Dentistry and Nursing, MVLS<br>Lee, Duncan; University of Glasgow<br>Gallacher, Katie I; University of Glasgow,<br>Mair, Frances; University of Glasgow, General Practice and Primary Care
**Primary Subject Heading**:	Respiratory medicine
Secondary Subject Heading:	General practice / Family practice
Keywords:	Multimorbidity, Polypharmacy, Chronic airways disease < THORACIC MEDICINE, Adverse events < THERAPEUTICS

SCHOLARONE™
Manuscripts

For peer review only

Examining Patterns of Multimorbidity, Polypharmacy and Risk of Adverse Drug Reactions in Chronic Obstructive Pulmonary Disease: A Cross-Sectional UK Biobank Study

Corresponding author

Professor Frances S Mair

General Practice and Primary Care, Institute of Health and Wellbeing, University of Glasgow, 1 Horelethill Road, Glasgow, G12 9LX, Scotland, United Kingdom

Tel: +44 141 330 8317

E-mail: frances.mair@glasgow.ac.uk

Authors:

Peter Hanlon[1], Barbara I. Nicholl[1], Bhautesh Dinesh Jani[1], Ross McQueenie[1], Duncan Lee[2], Katie I. Gallacher[1], Frances S Mair[1]

1. General Practice and Primary Care, Institute of Health and Wellbeing, University of Glasgow, 1 Horselethill Road, Glasgow, G12 9LX, Scotland, United Kingdom
2. School of Mathematics and Statistics, The mathematics and Statistics Building, University of Glasgow, University Place, Glasgow, G12 8SQ

Word count: 3446

For peer review only

For peer review only - http://bmjopen.bmj.com/site/about/guidelines.xhtml

Abstract

Objective: This study aims: (1) to describe the pattern and extent of multimorbidity and polypharmacy in UK Biobank participants with chronic obstructive pulmonary disease (COPD); and (2) to identify which comorbidities are associated with increased risk of adverse drug reactions (ADRs) resulting from polypharmacy.

Design: Cross-sectional.

Setting: Community cohort.

Participants: UK Biobank participants comparing self-reported COPD (n=8317) with no COPD (n=494,323).

Outcomes: Multimorbidity (≥ four conditions) and polypharmacy (≥ five medications) in participants with COPD versus those without. Risk of ADRs (taking ≥ three medications associated with falls, constipation, urinary retention, central nervous system (CNS) depression, bleeding or renal injury) in relation to the presence of COPD and individual comorbidities.

Results: Multimorbidity was more common in participants with COPD than those without (17% vs. 4%). Polypharmacy was highly prevalent (52% with COPD taking ≥ five medications vs 18% in those without COPD). Adjusting for age, sex and socioeconomic status, those with COPD were significantly more likely than those without to be prescribed ≥3 medications contributing to falls (Odds ratio (OR) 2.27, 95% confidence interval (CI) 2.13 to 2.42), constipation (OR 3.42, 95% CI 3.10 to 3.77), urinary retention (OR 3.38, 95% CI 2.94 to 3.87), CNS depression (OR: 3.75, 95% CI 3.31 to 4.25), bleeding (OR 4.61, 95% CI 3.35 to 6.19) and renal injury (OR 2.22, 95% CI 1.86 to 2.62). Concomitant cardiovascular disease was associated with the greatest risk of taking ≥3 medications associated with falls/renal injury. Concomitant mental health conditions were most strongly associated with medications linked with CNS depression/urinary retention/bleeding.

Conclusions: Multimorbidity is common in COPD and associated with high levels of polypharmacy. Co-prescription of drugs with various ADRs is common. Future research should examine the effects

For peer review only - http://bmjopen.bmj.com/site/about/guidelines.xhtml

on healthcare outcomes of co-prescribing multiple drugs with similar potential ADRs. Clinical guidelines should emphasise assessment of multimorbidity and ADR risk.

Abstract word count: 300

Strengths and Limitations

- This paper assesses multimorbidity, polypharmacy and risk of adverse drug reactions are assessed in UK Biobank participants with self-reported COPD compared with those without COPD.
- Baseline variables from the UK Biobank assessment centre were used to adjust for potential confounders.
- Cumulative risk of common adverse drug reactions was quantified by identifying UK Biobank participants taking three or more medications associated with similar adverse drug reactions.
- Analyses were repeated using a subgroup of participants with spirometry data confirming airflow obstruction.
- Medication and comorbidity data rely on participant self-report, and may thus be susceptible to bias or inaccuracy.

For peer review only

BACKGROUND

In people with Chronic Obstructive Pulmonary Disease (COPD), multimorbidity (the presence of two or more long-term conditions (LTCs)) is highly prevalent.(1-4) A recent meta-analysis of 29 datasets demonstrated that those with COPD are significantly more likely to be diagnosed with a range of cardiovascular comorbidities than those without COPD (we will use the term comorbidity when referring to specific conditions in addition to COPD, and multimorbidity to refer to the presence of two or more LTCs).(5) Other LTCs with known increased prevalence in COPD include obesity,(6) depression,(7-10) gastro-oesophageal reflux disease,(11-13) osteoporosis,(14-16) and lung cancer.(17, 18) Each of these conditions has been associated with poorer health related outcomes in COPD when compared to those with no comorbidity.(19-30) The overall burden of multimorbidity also impacts prognosis in COPD, for example higher number of comorbidities is associated with higher risk of mortality,(31) and higher burden of morbidity assessed using the Charlson index and the COPD-specific comorbidity test (COTE) is associated with higher risk of all-cause and respiratory specific mortality.(32, 33) The importance of considering the impact of multimorbidity in the management of long-term conditions is increasingly recognised, however an immature evidence base means that disease specific guidelines often lack specific recommendations with respect to multimorbidity.(34) The prevalence and prognostic significance of multimorbidity in COPD make it a potentially useful exemplar condition in which to consider the specific implications of different patterns of multimorbidity. Polypharmacy is one such implication.

Multimorbidity in the general population is associated with polypharmacy (often defined as concomitant use of ≥5 or ≥10 pharmacological agents).(35) Polypharmacy has been associated with increased risk of adverse drug reactions (ADRs)(36-38) and potentially preventable hospital admissions, particularly in the elderly.(39, 40) It has been demonstrated that diagnosis of COPD is associated with increased risk of polypharmacy.(41, 42) This is, in large measure, due to the high

For peer review only - http://bmjopen.bmj.com/site/about/guidelines.xhtml

burden of extra-pulmonary comorbidities.(43) However, little is known about the risk of ADRs in the context of multimorbidity in COPD.

Given the well-established burden of multimorbidity and polypharmacy in COPD, it is likely that those with COPD are at increased risk of ADRs resulting from polypharmacy. Previous analyses have not focused on the risk of specific ADRs, or assessed which LTCs increase this risk, instead reporting overall counts of prescribed medication. Data collected for the UK Biobank cohort offers an opportunity to assess how multimorbidity in COPD relates to polypharmacy and to assess the prevalence of co-prescription of medications with similar ADRs.

This paper aims:

- To describe the pattern and extent of multimorbidity and polypharmacy in UK Biobank participants with COPD.
- To identify which LTCs in people with COPD are associated with increased risk of ADRs resulting from polypharmacy.

For peer review only - http://bmjopen.bmj.com/site/about/guidelines.xhtml

METHODS

Data collection

The UK Biobank is a large, population cohort that recruited voluntary participants from throughout the United Kingdom. Between 2006 and 2010, UK Biobank recruited 502,640 participants aged 37 to 73. Participants underwent baseline assessments at one of 22 assessment centres throughout England, Scotland and Wales. Sociodemographic and lifestyle details were recorded using touchscreen questionnaires. Townsend scores were derived from participant postcodes to provide an area-based measure of socioeconomic deprivation. Self-reported LTCs, prescribed and over-the-counter medications, smoking status (current, previous or never) and frequency of alcohol intake (never / special occasions only, one-three times a month, at least once a week) were recorded from a touchscreen questionnaire and subsequent verbal interview with a study nurse. Physical activity was self-reported based on a questionnaire administered in the UK Biobank assessment centre http://biobank.ctsu.ox.ac.uk/crystal/field.cgi?id=6164. We classified the responses into: none (no physical activity in the last four weeks), low (light 'DIY' activity only in the last four weeks), medium (heavy DIY and/or walking for pleasure and/or other exercises in the last four weeks), and high (strenuous sports in the last four weeks).

Study centre staff also collected physical measures including height and weight (to calculate body mass index (BMI)) and spirometry. Spirometry was performed using a Vitalograph Pneumotrac 6800. Individual reasons for contraindications to attempting spirometry were not recorded but, according to protocol, these included chest infection in the last month, history of collapsed lung, and heart attack or surgery in the past three months. Full details of the Biobank spirometry protocol are available at https://biobank.ctsu.ox.ac.uk/crystal/docs/Spirometry.pdf. In brief, participants were allowed up to three attempts to provide two reproducible spirometry measurements. Where the reproducibility of the first two was deemed acceptable (<5% variation in both FEV1 and FVC) a third measurement was not performed. All values were recorded along with any error messages

For peer review only - http://bmjopen.bmj.com/site/about/guidelines.xhtml

generated. As per the American Thoracic Society/European Respiratory Society (ATS/ERS) end-of-test criteria, we interpreted as valid any measurement with no error message or if 'user accepted' was specified.(44) No post-bronchodilator measurements were recorded, which deviates from the ATS/ERS guidelines and the Global Initiative for Chronic Obstructive Lung Disease (GOLD) guidelines for COPD.(45, 46)

Participants provided full informed consent to participate in UK Biobank and this study had full ethical approval from the NHS National Research Ethics Service for UK Biobank studies (Ref 16/NW/0274); this study is part of UK Biobank approved project number 14151.

Defining COPD

Participants reporting to have been diagnosed with chronic obstructive pulmonary disease, chronic bronchitis, or emphysema at the nurse-led interview were coded as having 'self-reported COPD'.

Due to the potential inaccuracies of using self-reported diagnoses, we identified a subset of those with self-reported COPD who met an adaptation of the Global Initiative for Obstructive Lung Disease (GOLD) spirometry criteria for COPD.(46) This subset, referred to as 'GOLD COPD', was used as a sensitivity analysis for self-reported COPD, and to stratify findings by severity of airflow obstruction. For participants with self-report COPD and valid spirometry measurements, we calculated the ratio of forced expiratory volume in 1 second (FEV1) to forced vital capacity (FVC) using the highest measurement for each participant meeting the American Thoracic Society/European Respiratory Society end-of-test criteria.(44) Those with a FEV1/FVC ratio <0.7 were classed as having an obstructive deficit and thus meeting the GOLD diagnostic criteria for COPD. We used the Hankinson equation,(47) based on recorded age, sex and height, to calculate predicted FEV1 values for each participant. Those with GOLD COPD were classified on the basis of their best available FEV1

For peer review only - http://bmjopen.bmj.com/site/about/guidelines.xhtml

measurement as having mild (>80% predicted FEV1), moderate (50-80% predicted FEV1), or severe (<50% predicted FEV1) airflow obstruction in line with the GOLD COPD guidelines.(46)

Defining long term conditions and medications

All LTCs were defined by self-report. The list of included LTCs was taken from a list of 42 morbidities originally established for a large multimorbidity epidemiological study in Scotland, through systematic review, the Quality and Outcomes Framework, NHS Scotland and an expert panel (48), and subsequently amended for UK Biobank (49). The inclusion of 'other painful conditions' comprised LTCs in which pain is a predominant feature (particularly as this is likely to influence medication use). It should be noted that such a list is not exhaustive, but intended to cover common conditions frequently requiring prescription of analgesics (e.g. osteoarthritis, back pain, headaches etc.). Morbidities were categorised for the purposes of this analysis into cardiovascular disease, gastrointestinal disease, mental health conditions, cancer, and painful conditions/inflammatory arthropathies (comprising the list of 'other painful conditions' mentioned above, plus connective tissue diseases). Full details of conditions comprising each category can be found in appendix 1.

Medication data were collected by self-report. Medications were coded by mechanism of action according to the British National Formulary (BNF) (e.g. Angiotensin-converting enzyme inhibitors, beta-blockers, calcium channel blockers, etc.). For some situations where more than one medication with a similar mechanism of action may be commonly co-prescribed (e.g. aspirin and clopidogrel, both antiplatelets) these were coded separately. A complete list of the medications coded within each class can be found in appendix 2.

We defined those at risk of specific ADRs as anyone on 3 or more medications with similar potential ADRs, based on information provided in the *Scottish Government Model of Care Polypharmacy Working Group: Polypharmacy Guidance*.(50) This guideline cross-tabulates commonly prescribed

For peer review only - http://bmjopen.bmj.com/site/about/guidelines.xhtml

medications with common ADRs to help identify those at cumulative risk of ADRs. This document groups common medications by similar potential ADRs. While this list is not all-inclusive, and the cut-off value of three or more medications is arbitrary, this does allow an estimation of the cumulative risk of specific ADRs. We identified six potential ADRs (falls/fractures, constipation, urinary retention, CNS depression, bleeding and renal injury) for which the proportion of participants taking three or more associated medications could be assessed. It should be noted that several of these event (e.g. falls/fractures, CNS depression) are often multifactorial, and medication may be a contributing factor rather than a definitive cause. As the guideline acknowledges, however, these are clinical events of which the risk is increased by taking multiple associated medications.

Statistical analysis

Study hypothesis was made an analyses planned prior to inspection of the data.

Baseline variables

Comparisons were made between participants with self-reported COPD and the rest of the cohort (who did not report COPD). Age, sex, smoking status, deprivation (Townsend score), BMI, physical activity and frequency of alcohol intake were compared using χ^2 test for categorical variables, χ^2 test for trend for ordinal variables, and Mann-Whitney-U test for continuous variables. Total number of morbidities, prevalence of specific morbidities, number of self-reported prescribed medications, and proportion of participants taking each class of medication (Appendix 2), were also compared between those with self-reported COPD and the rest of the cohort. All comparisons were repeated comparing participants with GOLD COPD only with those without COPD, stratifying by severity of airflow obstruction.

Multimorbidity and polypharmacy

For peer review only - http://bmjopen.bmj.com/site/about/guidelines.xhtml

Logistic regression analyses were used to compare participants with self-reported COPD and those without COPD. Odds ratios (OR) and 95% confidence intervals (95% CI) were calculated for:

- the presence of cardiovascular disease, cancer, gastrointestinal disease, mental health conditions and painful conditions/inflammatory arthropathies
- the presence of four or more morbidities (excluding COPD)
- the use of five or more, and 10 or more, medications (two separate models)

Models were initially adjusted for age, sex and socioeconomic deprivation (model 1), then adjusted for the addition of smoking status, alcohol frequency, BMI and physical activity (model 2). These analyses were repeated comparing those with GOLD COPD only to those without COPD.

Risk of ADRs

For each potential ADR (falls/fractures, constipation, urinary retention, CNS depression, bleeding and renal injury) participants taking three or more medications associated with that ADR were identified. The following comparisons were then made:

- Unadjusted percentages at risk of each ADR were calculated for participants without COPD, with self-reported COPD, and with self-reported COPD plus each category of LTC (cardiovascular disease, cancer, gastrointestinal disease, mental health conditions and painful conditions/inflammatory arthropathies) to give an impression of the ADR risk in COPD, and identify LTCs in those with COPD that may increase this risk.
- ORs of being at risk of each ADR were calculated comparing those with self-reported COPD to those without COPD adjusting for age, sex and socioeconomic deprivation (model 1) and for age, sex, socioeconomic deprivation, smoking status, alcohol frequency, BMI and physical activity (model 2).
- ORs of being at risk of each ADR were calculated comparing those with and without self-reported COPD in each LTC category to (i.e. participants with cardiovascular disease alone

For peer review only - http://bmjopen.bmj.com/site/about/guidelines.xhtml

compared to participants with cardiovascular disease plus COPD, etc.). This was intended to identify whether specific patterns of multimorbidity in COPD are associated with increased ADR risk. Adjustment for a wide range of potential confounders was not appropriate in these models due to the smaller number of participants in each model.

Each analysis was repeated comparing GOLD COPD only to those without COPD. Less than 3% of participants (with or without COPD) had missing data for potential confounding variables (table 1). Those with missing data were excluded from adjusted analyses. Spirometry data were missing for 3591 participants with self-report COPD (43%), hence the use of the GOLD COPD subset as a sensitivity analysis.

All analyses were performed using R statistical software (version 3.3.1).

For peer review only

For peer review only - http://bmjopen.bmj.com/site/about/guidelines.xhtml

RESULTS

At the time of recruitment, 8317 out of 502,619 participants reported having COPD (1.7%) and are referred to here as the self-report COPD group. Of those who self-reported COPD, 4726 (57%) had valid spirometry measurements. Spirometry was contraindicated or not available in 2507 of those with self-reported COPD. Spirometry measurements did not meet the ATS/ESR end-of-test criteria in 1084 participants.(44) Of those with valid spirometry, 2620 (55%) met the GOLD criteria for airflow obstruction (399 (15%) mild, 1409 (54%) moderate, 812 (31%) severe, see Figure 1) and are referred to here as GOLD COPD.

Baseline variables

Table 1 describes and compares the characteristics of those with and without COPD in UK Biobank. Participants with COPD (both self-report and GOLD) were significantly older, more socioeconomically deprived, and less physically active. A higher proportion of those with COPD were male, obese and had a history of smoking.

For peer review only - http://bmjopen.bmj.com/site/about/guidelines.xhtml

Table 1. Baseline variables								
**Characteristic**	**No COPD n=494323**		**COPD (self-report) n=8317**			**GOLD GOPD n=2620**		
	Count	%	Count	%	p-value	Count	%	p-value
**Sex**								
Male	224906	45.5	4268	51.3		1426	54.4	
Female	269417	54.5	4049	48.7	<0.001	1194	45.6	<0.001
**Age**	Median: 58		Median: 62			Median:		
	IQR: 50-63		IQR: 57-66		<0.001	63		<0.001
						IQR: 59-66		
**Ethnicity**								
White	464770	94.5	8052	97.3		2620	100	
Other	26821	5.4	219	2.6	<0.001	0	0	<0.001
Missing	2732		46			0	0	
**Socioeconomic deprivation quintile**								
1 (least deprived)	99672	20.2	1015	12.2		309	11.8	
2	98977	20.0	1142	13.7		362	13.8	
3	99013	20.1	1399	16.8		440	16.8	
4	98660	20.0	1735	20.9		580	22.2	
5 (most deprived)	98385	19.7	3015	36.3	<0.001	926	35.4	<0.001
Missing	616		11			3		
**Smoking status**								
Current	50817	10.3	2172	26.3		833	32.2	
Previous	169015	34.4	4083	49.5		1398	54.0	
Never	271602	55.3	1999	24.2	<0.001	360	13.9	<0.001
Missing	2889		63			29		
**Alcohol frequency**								
Daily	100070	20.3	1720	20.7		618	23.6	
3-4 times/week	114058	23.1	1404	16.9		475	18.2	
1-2 times/week	127459	25.9	1863	22.5		561	21.5	
1-3 times/month	54979	11.2	894	10.8		289	11.1	
Occasional	56707	11.5	1322	15.9		387	14.8	<0.001
Never	39569	8.0	1092	13.2	<0.001	284	10.9	
Missing	1481		22			6		
**BMI**								
<18.5	2478	0.5	148	1.8		56	2.2	
18.5-24.9	155282	31.8	2185	26.8		829	31.9	
25.0-29.9	211102	43.2	3165	38.9		1049	40.4	
>30	119813	24.5	2647	32.5	<0.001	665	25.6	<0.001
Missing	5648		172			21		
**Physical activity**								
High	49827	10.6	250	3.1		70	2.7	
Medium	387766	79.6	5838	72.0		1902	73.3	
Low	18354	3.8	589	7.3		203	7.8	
None	31425	6.4	1433	17.7	<0.001	421	16.2	<0.001
Missing	6951		207			24		
**FEV1 (% predicted)**								
>80	272109	78.0	1853	39.2		399	15.2	
50-79	71727	20.6	2022	42.8		1409	53.8	
<50	4841	1.4	851	18.0	<0.001	812	31.0	<0.001
Missing	145646		3591			1061		

For peer review only - http://bmjopen.bmj.com/site/about/guidelines.xhtml

Multimorbidity and polypharmacy

Prevalence of each category of comorbidity was higher in those with COPD than without (table 2). After controlling for age, sex and socioeconomic status, those with self-reported COPD were significantly more likely than those without to have each category of LTC examined: cardiovascular disease (OR 1.45; 95% confidence interval (CI) 1.39 to 1.52) , cancer (1.29; 1.2 to 1.39), gastrointestinal disease (1.76; 1.67 to 1.86) , mental health conditions (2.02; 1.89 to 2.15) , and painful conditions (1.54; 1.46 to 1.62). Results for GOLD COPD also suggested higher likelihood of each LTC compared to those without COPD, although the ORs were lower and results for cancer not statistically significant (appendix 3). Results were similar after adjusting for additional confounders (smoking status, alcohol frequency, BMI and physical activity) with the exception of cardiovascular disease in GOLD COPD, which was no longer significantly associated (1.08; 0.99 to 1.18) (appendix 3).

For peer review only - http://bmjopen.bmj.com/site/about/guidelines.xhtml

Table 2: Long Term Conditions in those with and without COPD

	Control	Self-report COPD	GOLD COPD			
			**All**	**Mild**	**Moderate**	**Severe**
	n=494323<br>count (%)	n=8317<br>count (%)	n=2620<br>count (%)	n=399<br>count (%)	n=1409<br>count (%)	n= 812<br>count (%)
**Total comorbid-ities (excluding COPD)**						
≥4	19959 (4.0)	1389 (16.7)**	331 (12.6)**	46 (11.5)	191 (13.5)	94 (11.6)
**Total number of medications**						
≥1	356406 (72.1)	7670 (92.2)**	2452 (93.6)**	352 (88.2)	1321 (93.8)	779 (95.9)
≥5	87286 (17.7)	4312 (51.8)**	1349 (51.5)**	171 (42.9)	702 (49.8)	476 (58.6)
≥10	10678 (2.2)	1269 (15.3)**	329 (12.6)**	31 (7.8)	172 (12.2)	126 (15.5)
**Prevalence of comorbidities**						
**Cardiovascular**	**152891 (30.9)**	**3957 (47.6)****	**1156 (44.1)****	**142 (35.6)**	**611 (43.4)**	**403 (49.6)**
Hypertension	130119 (26.3)	3206 (38.5)**	916 (35.0)**	112 (28.1)	483 (34.3)	321 (39.5)
CHD	21560 (4.4)	1171 (14.1)**	315 (12.0)**	31 (7.6)	185 (13.1)	99 (12.2)
Diabetes	24737 (5.0)	766 (9.1)**	189 (7.2)**	16 (4.0)	109 (7.7)	64 (7.9)
Stroke/TIA	8459 (1.7)	395 (4.7)**	98 (3.7)**	11 (2.8)	51 (3.6)	36 (4.4)
AF	3552 (0.7)	99 (1.2)**	34 (1.3)**	3 (0.8)	16 (1.1)	15 (1.8)
Heart failure	768 (0.2)	35 (0.4)**	6 (0.2)	0	1 (0.1)	5 (0.6)
**Respiratory**						
Asthma	55245 (11.2)	3048 (36.6)**	984 (37.6)**	142 (35)	523 (37.1)	319 (39.3)
PE/DVT	12316 (2.5)	554 (6.7)**	139 (5.3)**	29 (7.3)	71 (5.0)	39 (4.8)
Bronchiectasis	968 (0.2)	167 (2.0)**	39 (1.5)**	7 (1.8)	17 (1.2)	15 (1.8)
Pulmonary fib.	504 (0.1)	67 (0.8)**	18 (0.7)**	3 (0.8)	12 (0.9)	3 (0.4)
**Cancer**	**37686 (7.6)**	**937 (11.3)**	**272 (10.4)**	**47 (11.8)**	**146 (10.4)**	**79 (9.7)**
Lung	405 (0.1)	52 (0.6)**	15 (0.6)**	0	7 (0.5)	8 (1.0)
Breast	11311 (2.3)	210 (2.5)*	57 (2.2)	12 (3.0)	30 (2.1)	15 (1.8)
Prostate	3588 (0.7)	105 (1.3)**	30 (1.1)*	5 (1.3)	12 (0.9)	13 (1.6)
GI	2925 (0.6)	96 (1.2)**	34 (1.3)**	6 (1.5)	19 (1.3)	9 (1.1)
Haem	6170 (1.2)	124 (1.5)*	34 (1.3)	5 (1.3)	17 (1.2)	12 (1.5)
**Gastrointestinal**	**55635 (11.5)**	**1737 (20.9)****	**468 (17.9)****	**76 (19.0)**	**254 (18.0)**	**138 (17.0)**
Dyspepsia	37819 (7.7)	1257 (15.1)**	348 (13.3)**	53 (13.3)	189 (13.4)	106 (13.1)
Diverticular dis	5181 (1.0)	224 (2.7)**	54 (2.1)**	6 (1.5)	32 (2.3)	16 (2.0)
IBS	11203 (2.3)	291 (3.5)**	64 (2.4)**	17 (4.3)	35 (2.5)	12 (1.5)
CLD	935 (0.2)	36 (0.4)**	10 (0.4)*	2 (0.5)	10 (0.7)	3 (0.4)
**Mental Health**	**35822 (7.2)**	**1127 (13.6)****	**304 (11.6)****	**54 (13.5)**	**162 (11.5)**	**88 (10.8)**
Depression	27578 (5.6)	901 (10.8)**	233 (8.9)**	42 (10.5)	128 (9.1)	63 (7.8)
Anxiety	8781 (1.8)	245 (2.9)**	69 (2.6)**	13 (3.3)	36 (2.6)	20 (2.5)
Schizophrenia/ bipolar	1918 (0.4)	79 (0.9)**	27 (1.0)**	3 (0.7)	15 (1.1)	9 (1.1)
**Other**						
Other painful	81733 (16.5)	2259 (27.2)**	655 (25.0)**	115 (28.8)	367 (26.0)	173 (21.3)
Osteoporosis	7700 (1.6)	342 (4.1)**	128 (4.9)**	21 (5.3)	67 (4.8)	40 (4.9)
Connective tissue disease	10642 (2.2)	391 (4.7)**	112 (4.3)**	19 (4.8)	72 (5.1)	21 (2.6)

Compared with control (χ^2): * : p<0.05, ** : p<0.001

For peer review only - http://bmjopen.bmj.com/site/about/guidelines.xhtml

Morbidity counts (excluding COPD) and counts of prescribed medication are shown in table 2 comparing those with COPD, stratified by severity of airflow obstruction, with those without. Those with COPD had higher numbers of LTCs and more prescribed medications than those without. There was a trend towards more prescribed medications in those with greater severity of airway obstruction. After controlling for age, sex and socioeconomic status, those with self- report COPD were more likely to report ≥4 LTCs (3.49; 3.28 to 3.71), ≥5 medications (3.85; 3.68 to 4.03), and ≥10 medications (5.72; 5.36 to 6.10) than those without COPD. Results were similar for GOLD COPD and remained statistically significant after adjusting for smoking status, alcohol frequency, BMI and physical activity (appendix 3).

ADR Risk

Counts and percentages of participants taking specific medications are shown in appendix 4. Participants with COPD (self-report and GOLD) were more likely that those without COPD to be prescribed drugs across a range of disease areas, reflecting the range of LTCs present among those with COPD. The percentages of participants within each category (no COPD, COPD, and COPD with specific LTCs) taking three or more medications associated with a similar ADR is shown in Figure 2. For each category of ADR a higher proportion of participants with COPD reported taking three or more associated medications than those without COPD. This increased further with multimorbidity. Participants with COPD plus cardiovascular disease had the highest percentage taking three or more medications with a risk of falls or renal injury. Participants with COPD plus mental health conditions had the highest percentages taking three or more medications with a risk of constipation, CNS depression or bleeding.

After adjusting for age, sex and socioeconomic deprivation, those with self-report COPD remained more likely to be taking three or more medications in each category than those without COPD. These

For peer review only - http://bmjopen.bmj.com/site/about/guidelines.xhtml

findings remained statistically significant after adjusting for smoking status, alcohol frequency, BMI and physical activity (Table 3). Findings were similar for GOLD COPD however, after adjusting for additional potentially confounding variables, results for bleeding risk were not statistically significant in this sensitivity analysis (Table 3).

Table 3. Odds ratios (with 95% CI) for taking 3 of more medications associated with similar ADRs

ADR	Self-report COPD compared with no COPD N=502,640		GOLD COPD compared with no COPD N=496,943	
	Model 1 N=502,013	Model 2 N=487,718	Model 1 N=496,943	Model 2 N=482,378
	OR (95% CI)	OR (95% CI)	OR (95% CI)	OR (95% CI)
Falls	2.27 (2.13 – 2.42) ***	1.83 (1.71-1.96) ***	1.66 (1.47 – 1.87) ***	1.49 (1.30-1.69) ***
Constipation	2.71 (2.54 – 2.89) ***	2.66 (2.39-2.96) ***	2.18 (1.77 – 2.64) ***	1.82 (1.47-2.24) ***
Urinary retention	3.38 (2.94 – 3.87) ***	2.59 (2.22-3.0) ***	1.98 (1.44 – 2.64) ***	1.64 (1.18-2.21) **
CNS depression	3.75 (3.31 – 4.25) ***	2.81 (2.45-3.22) ***	2.29 (1.73 – 2.95) ***	1.87 (1.40-2.43) ***
Bleeding	4.60 (3.35 – 6.19) ***	3.39 (2.40-4.66) ***	2.63 (1.25 – 4.80) **	1.76 (0.75-3.48) §
Renal injury	2.22 (1.86 – 2.62) ***	1.84 (1.53-2.19) ***	1.94 (1.41 – 2.58) ***	1.84 (1.33-2.49) ***

§ : p>0.05 * : p<0.05, ** : p<0.01, *** : p<0.001
Model 1: Adjusted for age, sex and socioeconomic status
Model 2: Adjusted for age, sex, socioeconomic status, smoking, alcohol frequency, body mass index and physical activity

For peer review only

Finally, each category of ADR risk was assessed in a subgroup analysis for each category of LTC (cardiovascular, GI, cancer, mental health and painful conditions/inflammatory arthropathies) comparing those with and without COPD (e.g. participants with cardiovascular disease plus COPD compared with participants with cardiovascular disease alone, etc.). These models were adjusted for age, sex and socioeconomic status only. Within each category of LTC, those with self-reported COPD were more likely to be at risk of each ADR than those without COPD (appendix 3). Not all results were statistically significant when using GOLD COPD (Appendix 3).

For peer review only - http://bmjopen.bmj.com/site/about/guidelines.xhtml

DISCUSSION

Summary of main findings

Multimorbidity and polypharmacy in COPD were common among UK Biobank participants. The presence of multimorbidity was highly prevalent in those with COPD (85%). More than half reported polypharmacy (five or more medications), and 15% reported 10 or more medications. The prevalence of cardiovascular disease, as well as the degree of polypharmacy, was higher among those with more severe airflow obstruction.

For the first time, our data demonstrates that those with COPD were more likely than those without to be prescribed multiple medications (≥ three) with similar ADRs. Those with COPD plus cardiovascular disease were most likely to be taking multiple medications associated with increased risk of falls or renal injury, while those with COPD plus mental health conditions were most likely to be taking medications predisposing to constipation, CNS depression and bleeding.(50) Within each category of LTC, those with COPD were more likely to be taking multiple medications with similar ADRs than those without. These associations between patterns of multimorbidity and specific ADR risks have not been described or quantified previously.

For peer review only

Strengths and limitations

Strengths of this study include the large sample size with representation from different areas of the UK. The range of data collected at UK Biobank assessment centres meant it was possible to compare a range of sociodemographic characteristics as well as spirometry data, the latter being unusual for a large community based cohort. It is recognised, however, that UK Biobank participants show some evidence of 'healthy volunteer bias', differing from the UK average on a number of socioeconomic, lifestyle and health-related measures. Specifically they are less socioeconomically deprived, less likely to smoke, to be obese, and have fewer self-reported health conditions.(51) All LTC diagnoses

For peer review only - http://bmjopen.bmj.com/site/about/guidelines.xhtml

as well as medication data were self-reported, with no alternative means of verification. We attempted to minimise this limitation by identifying a subset of those with COPD meeting the GOLD diagnostic criteria and repeating the analyses with this subset. Importantly, spirometry values were also pre-bronchodilator, which is in contravention to guidelines for diagnosing COPD. Additionally, information was not available about the strength of indication for medications and individual susceptibility to risk, which is a limitation when considering the risk of ADRs.

The use of the Scottish Government Polypharmacy Guideline allowed analysis of potential ADR risk by specific common ADRs. The intended purpose of this guideline, however, was not to identify potential risk from a population sample, but rather to identify potential causes of symptoms or complications. The analysis in this study, therefore, serves only as an approximation of potential risk, not an absolute marker of inappropriate polypharmacy. The cross-sectional nature of this analysis also precludes an analysis of actual harm as a result of polypharmacy. Many of the potential ADRs, such as falls and fractures and renal injury, and frequently multifactorial events and may not be directly attributable to medication use. Despite these limitations, however, the co-prescription of multiple medications with similar ADRs strongly implies greater potential for harm. The association of such prescribing patterns with COPD, across a range of potential ADRs, is clear from our findings. This analysis is, to the author's knowledge, the first to attempt to quantify this risk for specific ADRs in this way.

For peer review only

Context and implications

The increased prevalence of individual LTCs such as coronary heart disease, hypertension, diabetes, dyspepsia, osteoporosis, cancer, depression and anxiety in those with COPD is similar to the findings from other population based studies of multimorbidity in COPD.(5, 11, 52-54) Our finding that cardiovascular disease prevalence increased with increasing severity of COPD is in keeping with the

For peer review only - http://bmjopen.bmj.com/site/about/guidelines.xhtml

body of literature on cardiovascular disease and COPD, in which high prevalence has been observed in (usually older) cohorts with severe airflow limitation.(5, 21) Greater polypharmacy with greater severity of COPD has also been observed previously in older COPD populations,(42, 55) although such analyses have been smaller (n=1859 and 398, respectively) and have not assessed the specific patterns of prescribing in COPD. To the best of our knowledge, no previous studies have assessed the risk of ADRs as a result of polypharmacy in COPD. A recent population-based analysis of prescribing data from 310,000 adults in Scotland showed that over 15 years from 1995 to 2010 the proportion of people with polypharmacy and with potentially serious drug-to-drug interactions increased dramatically.(36) The number of prescribed medications was also associated with increased risk of interactions. Our analysis differs in approach from this analysis, by seeking to identify patterns of prescribing increasing risk of specific adverse events, rather than counting total potential interactions. The strength of our approach lies in highlighting specific patterns of multimorbidity in which specific ADRs are more likely. Our findings can therefore be applied to clinical practice, highlighting the importance of recognising multimorbidity in COPD and being alert to specific ADRs when prescribing medication.

Our findings indicate that in those with COPD the potential for ADRs as a result of combinations of medications is high, and this appears to be the result of a high prevalence of extra-pulmonary LTCs. Clinical guidelines for COPD should place greater emphasis on the need for assessment of associated multimorbidity and the risk of associated ADRs. While our analysis shows potential areas where ADR risk exists in COPD (e.g. falls in those with concomitant cardiovascular disease, CNS depression, constipation with concomitant mental health conditions), future research is merited to assess what actual harm could be attributed to such prescribing patterns.

Conclusion

For peer review only

For peer review only - http://bmjopen.bmj.com/site/about/guidelines.xhtml

Among UK Biobank participants with COPD there was considerable multimorbidity and polypharmacy. Those with COPD were highly likely to be concurrently prescribed multiple medications with similar potential adverse effects. Medications contributing to this risk were largely indicated for the management of the associated morbidities rather than COPD. Future research should examine the effects on healthcare outcomes of co-prescribing of multiple drugs with similar potential of ADRs. Clinical guidelines for COPD should emphasise the need for assessment of multimorbidity and the risk of associated ADRs.

Figure Legends

Figure 1: Flow diagram of identification of participants with 'self-report COPD' and 'GOLD COPD'.

Figure 2: Bubble plot showing percentage of participants in each comorbidity category taking 3 or more concomitant medications associated with specific adverse drug reactions.

Ethics approval and consent to participate

Participants provided full informed consent to participate in UK Biobank and this study was covered by the generic ethical approval for UK Biobank studies from the NHS National Research Ethics Service (Ref 16/NW/0274).

Availability of data and materials

UK Biobank data is available via *www.ukbiobank.ac.uk*. Syntax for the generation of derived variables and for the analysis used for this study will be submitted to UK Biobank for record.

For peer review only - http://bmjopen.bmj.com/site/about/guidelines.xhtml

Competing interests

The authors declare that they have no competing interests

Funding

This study was supported by a CSO Catalyst Grant CGA/16/39. BDJ was funded by a NHS Research for Scotland career research fellowship.

Author contributions

All authors (PH, BN, BJ, RM, DL, KG and FM) were involved in the conceptualisation and design of the project and interpretation of results. PH carried out the analysis with support from BJ, RM and BN. DL provided statistical support. All authors had access to the data. PH wrote the first draft of the paper and all authors commented on subsequent drafts. All authors approved the final draft for publication. FM is guarantor.

Acknowledgements

This research has been conducted using the UK Biobank Resource, approved project number 14151.

References

For peer review only

For peer review only - http://bmjopen.bmj.com/site/about/guidelines.xhtml

1. Anecchino C, Rossi E, Fanizza C, De Rosa M, Tognoni G, Romero M. Prevalence of chronic obstructive pulmonary disease and pattern of comorbidities in a general population. International journal of chronic obstructive pulmonary disease. 2007;2(4):567-74.
2. Fumagalli G, Fabiani F, Forte S, Napolitano M, Marinelli P, Palange P, et al. INDACO project: a pilot study on incidence of comorbidities in COPD patients referred to pneumology units. Multidisciplinary Respiratory Medicine. 2013;8.
3. Putcha N, Puhan MA, Hansel NN, Drummond MB, Boyd CM. Impact of co-morbidities on self-rated health in self-reported COPD: An analysis of NHANES 2001-2008. Copd-Journal of Chronic Obstructive Pulmonary Disease. 2013;10(3):324-32.
4. Garcia-Olmos L, Alberquilla A, Ayala V, Garcia-Sagredo P, Morales L, Carmona M, et al. Comorbidity in patients with chronic obstructive pulmonary disease in family practice: a cross sectional study. BMC family practice. 2013;14.
5. Chen W, Thomas J, Sadatsafavi M, FitzGerald JM. Risk of cardiovascular comorbidity in patients with chronic obstructive pulmonary disease: a systematic review and meta-analysis. Lancet Respir Med. 2015;3(8):631-9.
6. Rodriguez DA, Garcia-Aymerich J, Valera JL, Sauleda J, Togores B, Galdiz JB, et al. Determinants of exercise capacity in obese and non-obese COPD patients. Respiratory Medicine. 2014;108(5):745-51.
7. Al-shair K, Dockry R, Mallia-Milanes B, Kolsum U, Singh D, Vestbo J. Depression and its relationship with poor exercise capacity, BODE index and muscle wasting in COPD. Respiratory Medicine. 2009;103(10):1572-9.
8. Di Marco F, Verga M, Reggente M, Casanova FM, Santus P, Blasi F, et al. Anxiety and depression in COPD patients: The roles of gender and disease severity. Respiratory Medicine. 2006;100(10):1767-74.
9. Qian J, Simoni-Wastila L, Rattinger GB, Lehmann S, Langenberg P, Zuckerman IH, et al. Associations of depression diagnosis and antidepressant treatment with mortality among young and disabled Medicare beneficiaries with COPD. General Hospital Psychiatry. 2013;35(6):612-8.
10. Atlantis E, Fahey P, Cochrane B, Smith S. Bidirectional Associations Between Clinically Relevant Depression or Anxiety and COPD A Systematic Review and Meta-analysis. Chest. 2013;144(3):766-77.
11. Bor S, Kitapcioglu G, Solak ZA, Ertilav M, Erdinc M. Prevalence of gastroesophageal reflux disease in patients with asthma and chronic obstructive pulmonary disease. Journal of Gastroenterology and Hepatology. 2010;25(2):309-13.
12. Garcia Rodriguez LA, Ruigomez A, Martin-Merino E, Johansson S, Wallander M-A. Relationship Between Gastroesophageal Reflux Disease and COPD in UK Primary Care. Chest. 2008;134(6):1223-30.
13. Kim J, Lee JH, Kim Y, Kim K, Oh Y-M, Yoo KH, et al. Association between chronic obstructive pulmonary disease and gastroesophageal reflux disease: a national cross-sectional cohort study. Bmc Pulmonary Medicine. 2013;13.
14. Ferguson GT, Calverley PMA, Anderson JA, Jenkins CR, Jones PW, Willits LR, et al. Prevalence and Progression of Osteoporosis in Patients With COPD Results From the Towards a Revolution in COPD Health Study. Chest. 2009;136(6):1456-65.
15. Jorgensen NR, Schwarz P, Holme I, Henriksen BM, Petersen LJ, Backer V. The prevalence of osteoporosis in patients with chronic obstructive pulmonary disease - A cross sectional study. Respiratory Medicine. 2007;101(1):177-85.
16. Ogura-Tomomatsu H, Asano K, Tomomatsu K, Miyata J, Ohmori N, Kodama M, et al. Predictors of Osteoporosis and Vertebral Fractures in Patients Presenting with Moderate-to-Severe Chronic Obstructive Lung Disease. Copd-Journal of Chronic Obstructive Pulmonary Disease. 2012;9(4):332-7.

For peer review only - http://bmjopen.bmj.com/site/about/guidelines.xhtml

17. Young RP, Hopkins RJ, Christmas T, Black PN, Metcalf P, Gamble GD. COPD prevalence is increased in lung cancer, independent of age, sex and smoking history. European Respiratory Journal. 2009;34(2):380-6.
18. Wilson DO, Weissfeld JL, Balkan A, Schragin JG, Fuhrman CR, Fisher SN, et al. Association of radiographic emphysema and airflow obstruction with lung cancer. American Journal of Respiratory and Critical Care Medicine. 2008;178(7):738-44.
19. Garcia-Rio F, Soriano JB, Miravitlles M, Munoz L, Duran-Tauleria E, Sanchez G, et al. Impact of Obesity on the Clinical Profile of a Population-Based Sample with Chronic Obstructive Pulmonary Disease. Plos One. 2014;9(8).
20. Cecere LM, Littman AJ, Slatore CG, Udris EM, Bryson CL, Boyko EJ, et al. Obesity and COPD: Associated Symptoms, Health-related Quality of Life, and Medication Use. Copd-Journal of Chronic Obstructive Pulmonary Disease. 2011;8(4):275-84.
21. Mannino DM, Thorn D, Swensen A, Holguin F. Prevalence and outcomes of diabetes, hypertension and cardiovascular disease in COPD. European Respiratory Journal. 2008;32(4):962-9.
22. Curkendall SM, DeLuise C, Jones JK, Lanes S, Stang MR, Goehring E, et al. Cardiovascular disease in patients with chronic obstructive pulmonary disease, Saskatchewan Canada cardiovascular disease in COPD patients. Annals of Epidemiology. 2006;16(1):63-70.
23. McGarvey LP, John M, Anderson JA, Zvarich M, Wise RA. Ascertainment of cause-specific mortality in COPD: operations of the TORCH Clinical Endpoint Committee. Thorax. 2007;62(5):411-5.
24. Zielinski J, MacNee W, Wedzicha J, Ambrosino N, Braghiroli A, Dolensky J, et al. Causes of death in patients with COPD and chronic respiratory failure. Monaldi archives for chest disease = Archivio Monaldi per le malattie del torace. 1997;52(1):43-7.
25. Mannino DM, Doherty DE, Buist AS. Global Initiative on Obstructive Lung Disease (GOLD) classification of lung disease and mortality: findings from the Atherosclerosis Risk in Communities (ARIC) study. Respiratory Medicine. 2006;100(1):115-22.
26. Campo G, Guastaroba P, Marzocchi A, Santarelli A, Varani E, Vignali L, et al. Impact of COPD on Long-term Outcome After ST-Segment Elevation Myocardial Infarction Receiving Primary Percutaneous Coronary Intervention. Chest. 2013;144(3):750-7.
27. Konecny T, Somers K, Orban M, Koskino Y, Lennon RJ, Scanlon PD, et al. Interactions Between COPD and Outcomes After Percutaneous Coronary Intervention. Chest. 2010;138(3):621-7.
28. Sidney S, Sorel M, Quesenberry CP, DeLuise C, Lanes S, Eisner MD. COPD and incident cardiovascular disease hospitalizations and mortality: Kaiser permanente medical care program. Chest. 2005;128(4):2068-75.
29. Sakae TM, Menezes Pizzichini MM, Zimermann Teixeira PJ, da Silva RM, Trevisol DJ, Pizzichini E. Exacerbations of COPD and symptoms of gastroesophageal reflux: a systematic review and meta-analysis. Jornal Brasileiro De Pneumologia. 2013;39(3):259-71.
30. Hattiholi J, Gaude GS. Prevalence and correlates of osteoporosis in chronic obstructive pulmonary disease patients in India. Lung India : official organ of Indian Chest Society. 2014;31(3):221-7.
31. Miller J, Edwards LD, Agusti A, Bakke P, Calverley PMA, Celli B, et al. Comorbidity, systemic inflammation and outcomes in the ECLIPSE cohort. Respiratory Medicine. 2013;107(9):1376-84.
32. Divo M, Cote C, de Torres JP, Casanova C, Marin JM, Pinto-Plata V, et al. Comorbidities and Risk of Mortality in Patients with Chronic Obstructive Pulmonary Disease. American Journal of Respiratory and Critical Care Medicine. 2012;186(2):155-61.
33. Budweiser S, Harlacher M, Pfeifer M. Co-morbidities and hyperinflation are independent risk factors of all-cause mortality in very severe COPD. COPD. 2014;11(4):388-400.
34. (NICE) NIfHaCE. Multimorbidity: clinical assessment and management (NICE Guideline 56). 2016.
35. Corsonello A, Pedone C, Corica F. Polypharmacy in elderly patients at discharge from the acute care hospital. Therapeutics and Clinical Risk Management. 2007;2007(3):1.

For peer review only - http://bmjopen.bmj.com/site/about/guidelines.xhtml

36. Guthrie B, Makubate B, Hernandez-Santiago V, Dreischulte T. The rising tide of polypharmacy and drug-drug interactions: population database analysis 1995-2010. BMC Medicine. 2015;13:74.

37. Bourgeois FT, Shannon MW, Valim C, Mandl KD. Adverse drug events in the outpatient setting: an 11-year national analysis. Pharmacoepidemiol Drug Saf. 2010;19(9):901-10.

38. Johnell K, Klarin I. The relationship between number of drugs and potential drug-drug interactions in the elderly: a study of over 600,000 elderly patients from the Swedish Prescribed Drug Register. Drug Saf. 2007;30(10):911-8.

39. Pirmohamed M, James S, Meakin S, Green C, Scott AK, Walley TJ, et al. Adverse drug reactions as cause of admission to hospital: prospective analysis of 18 820 patients. BMJ. 2004;329(7456):15-9.

40. Howard RL, Avery AJ, Slavenburg S, Royal S, Pipe G, Lucassen P, et al. Which drugs cause preventable admissions to hospital? A systematic review. British Journal of Clinical Pharmacology. 2007;63(2):136-47.

41. Nobili A, Marengoni A, Tettamanti M, Salerno F, Pasina L, Franchi C, et al. Association between clusters of diseases and polypharmacy in hospitalized elderly patients: results from the REPOSI study. European Journal of Internal Medicine. 2011;22(6):597-602.

42. Franssen FM, Spruit MA, Wouters EF. Determinants of polypharmacy and compliance with GOLD guidelines in patients with chronic obstructive pulmonary disease. Int J Chron Obstruct Pulmon Dis. 2011;6:493-501.

43. Patel A. Extrapulmonary Polypharmacy and Cardiovascular Medications in COPD. Thorax. 2009;64(Suppl IV):A5-A74.

44. Miller MR, Hankinson J, Brusasco V, Burgos F, Casaburi R, Coates A, et al. Standardisation of spirometry. European Respiratory Journal. 2005;26(2):319-38.

45. Qaseem A, Wilt TJ, Weinberger SE, Hanania NA, Criner G, van der Molen T, et al. Diagnosis and management of stable chronic obstructive pulmonary disease: a clinical practice guideline update from the American College of Physicians, American College of Chest Physicians, American Thoracic Society, and European Respiratory Society. Annals of internal medicine. 2011;155(3):179-91.

46. (GOLD) GIfCOLD. From the Global Strategy for the Diagnosis, Management and Prevention of COPD, Global Initiative for Chronic Obstructive Lung Disease (GOLD) 2017 2017 [Available from: Available from: http://goldcopd.org.

47. Hankinson JL, Odencrantz JR, Fedan KB. Spirometric reference values from a sample of the general U.S. population. American Journal of Respiratory & Critical Care Medicine. 1999;159(1):179-87.

48. Barnett K, Mercer SW, Norbury M, Watt G, Wyke S, Guthrie B. Epidemiology of Multimorbidity and Implications for Health Care, Research, and Medical Education: a Cross-Sectional Study. Lancet. 2012;380(9836):37-43.

49. Nicholl BI, Mackay D, Cullen B, Martin DJ, Ul-Haq Z, Mair FS, et al. Chronic multisite pain in major depression and bipolar disorder: cross-sectional study of 149,611 participants in UK Biobank

1. BMC Psychiatry. 2014;14:350.

50. Group SGMoCPW. Polypharmacy Guidance (2nd edition). In: Government S, editor. 2015.

51. Fry A, Littlejohns TJ, Sudlow C, Doherty N, Allen NE. The representativeness of the UK Biobank cohort on a range of sociodemographic, physical, lifestyle and health-related characteristics. . Journal of epidemiology and community health. 2016;70(Suppl 1):A26-A.

52. Putcha N, Han MK, Martinez CH, Foreman MG, Anzueto AR, Casaburi R, et al. Comorbidities of COPD have a major impact on clinical outcomes, particularly in African Americans. Chronic obstructive pulmonary diseases (Miami, Fla). 2014;1(1):105-14.

53. Bhattacharyya P, Paul R, Ghosh M, Dey R, Dey R, Barooah N, et al. Prevalence of osteoporosis and osteopenia in advanced chronic obstructive pulmonary disease patients. Lung India : official organ of Indian Chest Society. 2011;28(3):184-6.

For peer review only - http://bmjopen.bmj.com/site/about/guidelines.xhtml

54. de Torres JP, Bastarrika G, Wisnivesky JP, Alcaide AB, Campo A, Seijo LM, et al. Assessing the relationship between lung cancer risk and emphysema detected on low-dose CT of the chest. Chest. 2007;132(6):1932-8.

55. Diez-Manglano J, Barquero-Romero J, Mena PA, Recio-Iglesias J, Cabrera-Aguilar J, Lopez-Garcia F, et al. Polypharmacy in patients hospitalised for acute exacerbation of COPD. European Respiratory Journal. 2014;44(3):791-4.

For peer review only

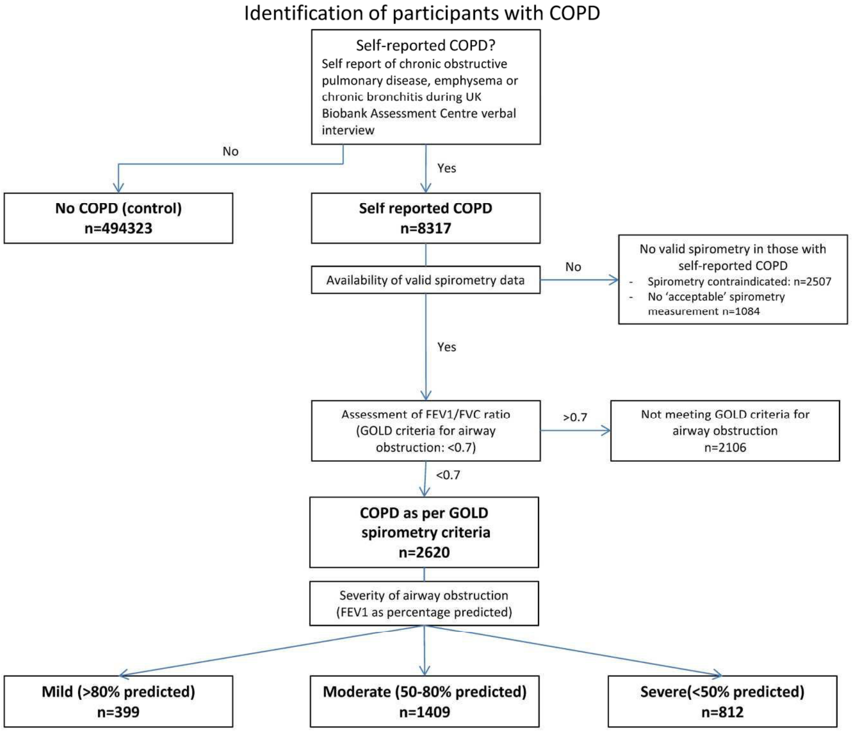

Flow diagram of identification of participants with 'self-report COPD' and 'GOLD COPD'.

190x142mm (300 x 300 DPI)

For peer review only

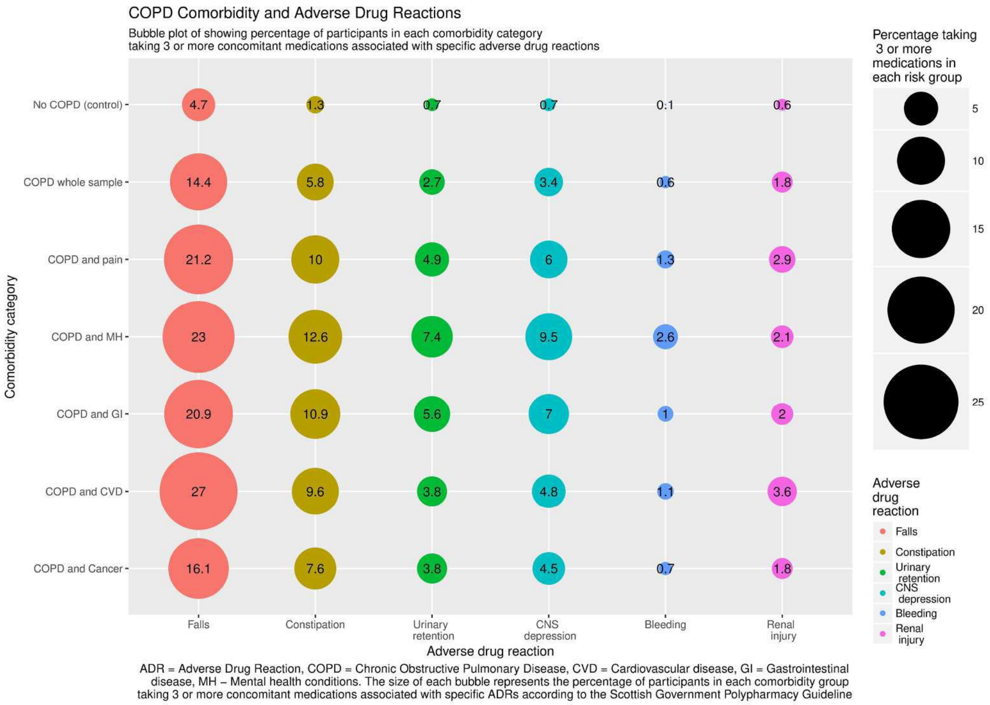

Bubble plot showing percentage of participants in each comorbidity category taking 3 or more concomitant medications associated with specific adverse drug reactions.

209x148mm (300 x 300 DPI)

For peer review only - http://bmjopen.bmj.com/site/about/guidelines.xhtml

Comorbidity category (used in analysis)	Conditions included (as reported in table 2)	Self-reported conditions comprising this condition (UK Biobank variables used to identify self-reported conditions)
Cardiovascular conditions	Hypertension	Hypertension<br>Essential hypertension
	Coronary heart disease	Heart attack/MI<br>Angina
	Diabetes	Diabetic nephropathy<br>Diabetic neuropathy/ulcers<br>Diabetes<br>Type 1 diabetes<br>Type 2 diabetes<br>Diabetic eye disease
	Stroke/TIA	Stroke<br>TIA<br>Subarachnoid haemorrhage<br>Brain haemorrhage<br>Ischaemic stroke
	Atrial fibrillation	Atrial fibrillation
	Heart failure	Cardiomyopathy<br>Hypertrophic cardiomyopathy<br>Heart failure/pulmonary oedema
	Peripheral vascular disease	Peripheral vascular disease<br>Leg claudication/intermittent claudication
Respiratory	COPD	COPD/Chronic obstructive pulmonary disease<br>Emphysema/Chronic bronchitis<br>Emphysema
	Asthma	Asthma
	PE/DVT	Deep vein thrombosis<br>Pulmonary emolism
	Bronchiectasis	Bronchiectasis
	Pulmonary fibrosis	Pulmonary fibrosis
Cancer	Cancer	“yes”/”no” to “have you ever had cancer?”
Gastrointestinal	Dyspepsia	Gastro-oesophageal reflux (GORD)<br>Oesophagitis/Barrett’s oesophagus<br>Gastric stomach ulcers<br>Gastric erosions/gastritis<br>Duodenal ulcer<br>Dyspepsia/indigestion<br>Hiatus hernia<br>Helicobacter pylori
	Diverticular disease	Diverticular disease/diverticulitis

For peer review only

	Irritable bowel syndrome	Irritable bowel syndrome
	Chronic liver disease	Oesophageal varices<br>Non infective hepatitis<br>Liver failure/cirrhosis<br>Primary biliary cirrhosis
	Inflammatory bowel disease	Inflammatory bowel disease<br>Crohn's disease<br>Ulcerative colitis
	Constipation	Constipation
	Viral hepatitis	Hepatitis B<br>Hepatitis C<br>Hepatitis D
Mental Health	Depression	Depression<br>Postnatal depression
	Anxiety	Anxiety/panic attacks<br>Nervous breakdown<br>Post-traumatic stress disorder<br>Obsessive compulsive disorder<br>Stress<br>Insomnia<br>Psychological/psychiatric problem
	Schizophrenia	Scizophrenia
	Bipolar	Mania<br>Bipolar disorder<br>Manic depression
Painful conditions	Connective tissue diseases	Myositis/myopathy<br>Systemic lupus erythematosus/SLE<br>Connective tissue disorder<br>Sjogren's syndrome.sicca syndrome<br>Dermatopolymyositis<br>Scloeroderma/systemic sclerosis<br>Rheumatoid arthritis<br>Psoriatic arthropathy<br>Dermatomyositis<br>Polymyositis<br>Polymyalgia rheumatica
	Other painful conditions	Back pain<br>Joint pain<br>Headaches (not migraine)<br>Sciatica<br>Plantar fasciitis<br>Carpal tunnel syndrome<br>Fibromyalgia<br>Arthritis<br>Shingles<br>Disc problem<br>Prolapsed disc/slipped disc

For peer review only

		Spine arthritis/spondylitis<br>Ankylosing spondylitis<br>Back problem<br>Osteoarthritis<br>Gout<br>Cervical spondylosis<br>Trigeminal neuralgia<br>Disc degeneration<br>Trapped nerve/compressed nerve
Other	Osteoporosis	Osteoporosis

For peer review only

Drugs with cumulative risk of Adverse Drug Reactions*	
Adverse Drug Reaction	Contributing drug classes (participants taking 3 or more considered ‘at risk’ for the purposes of analysis)
Falls	H2-receptor blockers<br>Loperamide<br>Prochlorperazine<br>Metoclopramide<br>ACE-inhibitor/Angiotensin receptor blocker<br>Thiazide diuretic<br>Loop diuretic<br>Amiloride/triamterene<br>Spironolactone<br>Beta-blocker<br>Calcium-channel blocker<br>Nitrates or nicorandil<br>Digoxin<br>Oral steroids<br>Opiates<br>Benzodiazepines<br>Sedative antihistamines<br>H1-receptor blockers<br>Antipsychotics<br>Tricyclic antidepressants<br>Sulfonylureas/gliptins/glinides<br>Pioglitazone<br>Urinary antispasmodics<br>Dosulepin<br>Alpha-blockers
Constipation	H2-receptor blockers<br>Laxatives<br>Loperamide<br>Prochlorperazine<br>Thiazide diuretics<br>Loop diuretics<br>Calcium-channel blockers<br>Opiates<br>Sedative antihistamines<br>H1-receptor blockers<br>Antipsychotics<br>Tricyclic antidepressants<br>Urinary antispasmodics<br>Dosulepin
Urinary retention	H2-receptor blockers<br>Loperamide<br>Prochlorperazine<br>Opiates<br>Sedative antihistamines<br>H1-receptor blockers<br>Antipsychotics<br>Tricyclic antidepressants

For peer review only

	Urinary antispasmodics<br>Dosulepin
CNS depression	H2-receptor blockers<br>Loperamide<br>Prochlorperazine<br>Oral steroids<br>Opiates<br>Benzodiazepines<br>Sedative antihistamines<br>H1-receptor blockers<br>Antipsychotics<br>Tricyclic antidepressants<br>Urinary antispasmodics<br>Dosulepin
Bleeding	Aspirin<br>Clopidogrel<br>Other antiplatelets<br>Oral steroids<br>SSRIs and related drugs<br>Non-steroidal anti-inflammatory drugs<br>Warfarin
Renal injury	ACE-inhibitor/angiotensin receptor blockers<br>Thiazide diuretic<br>Loop diuretic<br>Amiloride/triamterene<br>Spironolactone<br>Antibiotics/antifungals<br>Non-steroidal anti-inflammatory drugs
Adapted from *Scottish Government Model of Care Polypharmacy Working Group. Polypharmacy Guidance (2nd edition) March 2015. Scottish Government.*	

For peer review only

**Table S1. Odds ratios (with 95% CI) for the presence of categories of comorbidity**				
Comorbidity category	Self-report COPD compared with no COPD N=502,640		GOLD COPD compared with no COPD N=496,943	
	Model 1 N=502,013	Model 2 N=487,718	Model 1 N=496,324	Model 2 N=482,378
	OR (95% CI)	OR (95% CI)	OR (95% CI)	OR (95% CI)
Cardiovascular disease	1.45 (1.39-1.52) ***	1.28 (1.22-1.34) ***	1.11 (1.02-1.20) *	1.08 (0.99-1.18) §
Cancer	1.29 (1.20-1.39) ***	1.22 (1.13-1.31) ***	1.12 (0.99-1.27) §	1.06 (0.92-1.19) §
Gastrointestinal disease	1.76 (1.67-1.86) ***	1.56 (1.48-1.65) ***	1.4 (1.26-1.54) ***	1.24 (1.12-1.38) ***
Mental health	2.02 (1.89-2.15) ***	1.62 (1.51-1.73) ***	1.75 (1.54-1.97) ***	1.40 (1.22-1.58) ***
Painful conditions	1.62 (1.55-1.70) ***	1.41 (1.34-1.48) ***	1.40 (1.28-1.52) ***	1.24 (1.13-1.35) **

§ : p>0.05 * : p<0.05, ** : p<0.01, *** : p<0.001
Model 1: Adjusted for age, sex and socioeconomic status
Model 2: Adjusted for age, sex, socioeconomic status, smoking, alcohol frequency, body mass index and physical activity

**Table S2. Odds ratios (with 95% CI) for the presence of multimorbidity or polypharmacy**				
Outcome	Self-report COPD compared with no COPD N=502,640		GOLD COPD compared with no COPD N=496,943	
	Model 1 N=502,013	Model 2 N=487,718	Model 1 N=496,324	Model 2 N=482,378
	OR (95% CI)	OR (95% CI)	OR (95% CI)	OR (95% CI)
Multimorbidity (≥4 conditions)	3.49 (3.28-3.70) ***	2.79 (2.61-2.98) ***	2.34 (2.10-2.63) ***	1.99 (1.75-2.25) ***
Polypharmacy (≥5 medications)	3.85 (3.68-4.03) ***	3.30 (3.15-3.46) ***	3.47 (3.20-3.75) ***	3.20 (2.95-3.48) ***
Polypharmacy (≥10 medications	5.72 (5.36-6.10) ***	4.42 (4.11-4.75) ***	4.20 (3.72-4.73) ***	3.56 (3.12-4.05) ***

§ : p>0.05 * : p<0.05, ** : p<0.01, *** : p<0.001
Model 1: Adjusted for age, sex and socioeconomic status
Model 2: Adjusted for age, sex, socioeconomic status, smoking, alcohol frequency, body mass index and physical activity

**Table S3. Odds ratios (with 95% CI) for taking 3 of more medications associated with similar ADRs**				
ADR	Self-report COPD compared with no COPD N=502,640		GOLD COPD compared with no COPD N=496,943	
	Model 1 N=502,013	Model 2 N=487,718	Model 1 N=496,943	Model 2 N=482,378
	OR (95% CI)	OR (95% CI)	OR (95% CI)	OR (95% CI)
Falls	2.27 (2.13 – 2.42) ***	1.83 (1.71-1.96) ***	1.66 (1.47 – 1.87) ***	1.49 (1.30-1.69) ***
Constipation	2.71 (2.54 – 2.89) ***	2.66 (2.39-2.96) ***	2.18 (1.77 – 2.64) ***	1.82 (1.47-2.24) ***
Urinary retention	3.38 (2.94 – 3.87) ***	2.59 (2.22-3.0) ***	1.98 (1.44 – 2.64) ***	1.64 (1.18-2.21) **
CNS Depression	3.75 (3.31 – 4.25) ***	2.81 (2.45-3.22) ***	2.29 (1.73 – 2.95) ***	1.87 (1.40-2.43) ***
Bleeding	4.60 (3.35 – 6.19) ***	3.39 (2.40-4.66) ***	2.63 (1.25 – 4.80) **	1.76 (0.75-3.48) §
Renal injury	2.22 (1.86 – 2.62) ***	1.84 (1.53-2.19) ***	1.94 (1.41 – 2.58) ***	1.84 (1.33-2.49) ***

§ : p>0.05 * : p<0.05, ** : p<0.01, *** : p<0.001
Model 1: Adjusted for age, sex and socioeconomic status
Model 2: Adjusted for age, sex, socioeconomic status, smoking, alcohol frequency, body mass index and physical activity

For peer review only

Subgroup analyses – comparing COPD with no COPD among participants with specific categories of comorbidity

Table S4. Odds ratios (with 95% CI) for taking 3 of more medications associated with similar ADRs in participants with cardiovascular disease (CVD)

ADR	Self-report COPD plus CVD compared with CVD alone (no COPD) N=156,848	GOLD COPD plus CVD compared with CVD alone (no COPD) N=154,047
	Model 1 N=156,667	Model 1 N=153,852
	OR (95% CI)	OR (95% CI)
Falls	1.92 (1.79-2.07) ***	1.59 (1.39-1.82) ***
Constipation	2.89 (2.58-3.23) ***	2.06 (1.63-2.57) ***
Urinary retention	2.78 (2.33-3.28) ***	1.92 (1.30-2.72) ***
CNS Depression	3.17 (2.71-3.69) ***	2.17 (1.54-2.97) ***
Bleeding	4.00 (2.85-5.48) ***	2.26 (0.96-4.44) *
Renal injury	1.90 (1.59-2.25) ***	1.82 (1.31-2.45) ***

§ : p>0.05 * : p<0.05, ** : p<0.01, *** : p<0.001
Model 1: Adjusted for age, sex and socioeconomic status

Table S5. Odds ratios (with 95% CI) for taking 3 of more medications associated with similar ADRs in participants with cancer

ADR	Self-report COPD plus cancer compared with cancer alone (no COPD) N=38,623	GOLD COPD plus cancer compared with cancer alone (no COPD) N=37,958
	Model 1 N=38,575	Model 1 N= 37,912
	OR (95% CI)	OR (95% CI)
Falls	2.35 (1.95-2.81) ***	1.49 (1.00-2.13) *
Constipation	3.55 (2.73-4.56) ***	2.21 (1.22-3.68) **
Urinary retention	3.65 (2.52-5.13) ***	1.99 (0.78-4.14) §
CNS Depression	3.74 (2.66-5.14) ***	2.04 (0.86-4.04) §
Bleeding	4.69 (1.91-9.86) ***	2.20 (0.12-10.23) §
Renal injury	2.0 (1.17-3.20) **	2.26 (0.89-4.71) §

§ : p>0.05 * : p<0.05, ** : p<0.01, *** : p<0.001
Model 1: Adjusted for age, sex and socioeconomic status

Table S6. Odds ratios (with 95% CI) for taking 3 of more medications associated with similar ADRs in participants with gastrointestinal disease (GI)

ADR	Self-report COPD plus GI compared with GI alone (no COPD) N=58372	GOLD COPD plus GI compared with GI alone (no COPD) N=57103
	Model 1 N=58,299	Model 1 N=57,031
	OR (95% CI)	OR (95% CI)
Falls	2.18 (1.92-2.46) ***	1.46 (1.13-1.87) **
Constipation	2.70 (2.29-3.16) ***	1.58 (1.08-2.24) *
Urinary retention	2.64 (2.12-3.26) ***	1.46 (0.83-2.37) §
CNS Depression	3.02 (2.47-3.66) ***	1.50 (0.88-2.37) §
Bleeding	3.88 (2.27-6.25) ***	3.18 (0.97-7.63) §
Renal injury	1.99 (1.37-2.80) ***	1.22 (0.48-2.51) §

§ : p>0.05 * : p<0.05, ** : p<0.01, *** : p<0.001
Model 1: Adjusted for age, sex and socioeconomic status

For peer review only

**Table S7. Odds ratios (with 95% CI) for taking 3 of more medications associated with similar ADRs in participants with mental health conditions (MH)**		
ADR	Self-report COPD plus MH compared with MH alone (no COPD)<br>N=36,949	GOLD COPD plus MH compared with MH alone (no COPD)<br>N=36126
	Model 1<br>N=36,885	Model 1<br>N=36,065
	OR (95% CI)	OR (95% CI)
Falls	2.21 (1.90-2.56) ***	1.35 (0.99-1.82) §
Constipation	2.33 (1.93-2.81) ***	1.62 (1.08-2.34) *
Urinary retention	2.17 (1.71-2.74) ***	1.42 (0.82-2.29) §
CNS Depression	2.53 (2.04-3.12) ***	1.66 (1.03-2.54) *
Bleeding	2.86 (1.77-4.17) ***	1.94 (0.76-4.05) §
Renal injury	1.86 (1.19-2.79) **	1.27 (0.45-2.80) §
§ : p>0.05 * : p<0.05, ** : p<0.01, *** : p<0.001<br>Model 1: Adjusted for age, sex and socioeconomic status		

**Table S8. Odds ratios (with 95% CI) for taking 3 of more medications associated with similar ADRs in participants with painful conditions**		
ADR	Self-report COPD plus painful conditions compared with painful conditions alone (no COPD)<br>N=83,992	GOLD COPD plus painful conditions compared with painful conditions alone (no COPD)<br>N=82,388
	Model 1<br>N=83,895	Model 1<br>N=82,294
	OR (95% CI)	OR (95% CI)
Falls	1.99 (1.79-2.19) ***	1.45 (1.19-1.75) ***
Constipation	2.54 (2.21-2.91) ***	1.50 (1.10-2.00) **
Urinary retention	2.46 (2.03-2.96) ***	1.11 (0.64-1.75) §
CNS Depression	2.71 (2.28-3.21) ***	1.40 (0.90-2.06) §
Bleeding	3.50 (2.37-5.01) ***	2.20 (0.86-4.54) §
Renal injury	1.66 (1.30-2.09) ***	1.49 (0.93-2.25) §
§ : p>0.05 * : p<0.05, ** : p<0.01, *** : p<0.001<br>Model 1: Adjusted for age, sex and socioeconomic status		

For peer review only

Appendix 4: Specific medications in UK Biobank participants with and without COPD						
**Medications**	**Control** n=494323 count (%)	**Self-report COPD** n=8317 count (%)	**GOLD COPD**			
			**All** n=2620 count (%)	**Mild** n=399 count (%)	**Moderate** n=1409 count (%)	**Severe** n= 812 count (%)
**Total number of medications**						
≥1	356406 (72.1)	7670 (92.2)	2452 (93.6)	352 (88.2)	1321 (93.8)	779 (95.9)
≥5	87286 (17.7)	4312 (51.8)	1349 (51.5)	171 (42.9)	702 (49.8)	476 (58.6)
≥10	10678 (2.2)	1269 (15.3)	329 (12.6)	31 (7.8)	172 (12.2)	126 (15.5)
**Respiratory**						
Short acting B_2 agon.	22615 (4.6)	3328 (40.0)	1245 (47.5)	123 (30.8)	614 (43.6)	508 (62.6)
LABA	9819 (2.0)	2357 (28.3)	905 (34.5)	93 (23.3)	411 (29.2)	401 (49.3)
LAMA	597 (0.1)	1345 (16.2)	581 (22.2)	33 (8.3)	265 (18.8)	283 (34.9)
ICS	15309 (3.1)	2638 (31.7)	962 (36.7)	98 (24.6)	471 (33.4)	393 (48.4)
LABA+ICS	7259 (1.5)	1842 (22.1)	699 (26.7)	67 (16.8)	313 (22.2)	319 (39.3)
Prednisolone	3127 (0.6)	280 (3.4)	82 (3.1)	12 (3.0)	27 (1.9)	43 (5.3)
Mucolytic	174 (0.04)	187 (2.2)	49 (1.9)	1 (0.3)	10 (0.7)	38 (4.7)
**Cardiovascular**						
Antiplatelet	21817 (4.4)	894 (10.7)	268 (10.2)	31 (7.8)	158 (11.2)	79 (9.7)
ACE-inhibitor	44991 (9.1)	1276 (15.3)	367 (14.0)	33 (8.3)	198 (14.1)	136 (16.7)
ARB	17911 (3.6)	565 (6.8)	159 (6.1)	17 (4.3)	83 (5.9)	59 (7.2)
Calcium CB	14317 (2.9)	627 (7.5)	196 (7.5)	23 (5.8)	106 (7.5)	67 (8.3)
Statin	73439 (14.9)	2278 (27.4)	707 (27.0)	72 (18.0)	395 (28.0)	240 (29.6)
GTN	4425 (0.9)	373 (4.5)	110 (4.2)	8 (2.0)	70 (5.0)	32 (3.9)
ISMN	2814 (0.6)	244 (2.9)	68 (2.6)	5 (1.3)	42 (3.0)	21 (2.6)
Loop diuretic	4836 (1.0)	415 (5.0)	107 (4.1)	10 (2.5)	51 (3.6)	46 (5.7)
Thiazide	21961 (4.4)	637 (7.7)	196 (7.5)	22 (5.5)	108 (7.7)	66 (8.1)
Warfarin	4934 (1.0)	238 (2.9)	67 (2.6)	6 (1.5)	33 (2.3)	28 (3.4)
**Diabetes**						
Insulin	4643 (0.9)	161 (1.9)	35 (1.3)	2 (0.5)	23 (1.6)	3 (0.4)
Metformin	13754 (2.8)	448 (5.4)	102 (3.9)	7 (1.8)	57 (4.0)	38 (4.7)
Sulphonylurea	4901 (1.0)	158 (1.9)	35 (1.3)	2 (0.5)	17 (1.2)	16 (2.0)
Thiazolidindione	2212 (0.4)	60 (0.7)	17 (0.6)	1 (0.3)	10 (0.7)	6 (0.7)
**Gastrointestinal**						
PPI	42012 (8.5)	1989 (23.9)	522 (19.9)	79 (19.8)	286 (20.3)	157 (19.3)
Antacid	2435 (0.5)	146 (1.8)	25 (1.0)	7 (1.8)	10 (0.7)	8 (1.0)
H_2 RA.	7772 (1.6)	325 (3.9)	89 (3.4)	15 (3.8)	53 (3.8)	21 (2.6)
Laxative	5787 (1.8)	317 (3.8)	81 (3.1)	11 (2.8)	40 (2.8)	30 (3.7)
**Pain**						
Paracetamol	82376 (16.7)	2752 (33.1)	790 (30.2)	111 (27.8)	446 (31.6)	233 (28.7)
NSAID	45909 (9.3)	1149 (13.8)	319 (12.2)	50 (12.5)	175 (12.4)	94 (11.6)
Weak opiate	18736 (3.8)	1209 (14.5)	336 (12.8)	48 (12.0)	191 (13.6)	97 (11.9)
Strong opiate	1071 (0.2)	106 (1.8)	32 (1.2)	5 (1.3)	16 (1.1)	11 (1.4)
**Mental health**						
SSRI+related	15394 (3.1)	747 (9.0)	175 (6.7)	31 (7.8)	100 (7.1)	44 (5.4)
Tricyclic	4229 (0.9)	206 (2.5)	49 (1.9)	11 (2.8)	25 (1.8)	13 (1.6)
Antipsychotic	2237 (0.5)	107 (1.3)	30 (1.1)	5 (1.3)	16 (1.1)	9 (1.1)
Benzodiazepine	2316 (0.5)	182 (2.2)	47 (1.8)	6 (1.5)	28 (2.0)	13 (1.6)
**Metabolic**						
Thyroxine	20980 (4.2)	560 (6.7)	150 (5.7)	27 (6.8)	92 (6.5)	31 (3.8)
Bisphosphonate	3655 (0.7)	189 (2.3)	66 (2.5)	15 (3.8)	32 (2.3)	19 (2.3)

For peer review only

STROBE 2007 (v4) Statement—Checklist of items that should be included in reports of *cross-sectional studies*

Section/Topic	Item #	Recommendation	Reported on page #
**Title and abstract**	1	(*a*) Indicate the study's design with a commonly used term in the title or the abstract	2
		(*b*) Provide in the abstract an informative and balanced summary of what was done and what was found	2,3
**Introduction**			
Background/rationale	2	Explain the scientific background and rationale for the investigation being reported	4
Objectives	3	State specific objectives, including any prespecified hypotheses	5
**Methods**			
Study design	4	Present key elements of study design early in the paper	5,6,9
Setting	5	Describe the setting, locations, and relevant dates, including periods of recruitment, exposure, follow-up, and data collection	6,7
Participants	6	(*a*) Give the eligibility criteria, and the sources and methods of selection of participants	7
Variables	7	Clearly define all outcomes, exposures, predictors, potential confounders, and effect modifiers. Give diagnostic criteria, if applicable	6-8
Data sources/ measurement	8*	For each variable of interest, give sources of data and details of methods of assessment (measurement). Describe comparability of assessment methods if there is more than one group	6-8
Bias	9	Describe any efforts to address potential sources of bias	*14,15*
Study size	10	Explain how the study size was arrived at	7
Quantitative variables	11	Explain how quantitative variables were handled in the analyses. If applicable, describe which groupings were chosen and why	6,7,8,9
Statistical methods	12	(*a*) Describe all statistical methods, including those used to control for confounding	8-10
		(*b*) Describe any methods used to examine subgroups and interactions	9,10
		(*c*) Explain how missing data were addressed	10
		(*d*) If applicable, describe analytical methods taking account of sampling strategy	n/a
		(*e*) Describe any sensitivity analyses	9,10
**Results**			

For peer review only

Participants	13*	(a) Report numbers of individuals at each stage of study—eg numbers potentially eligible, examined for eligibility, confirmed eligible, included in the study, completing follow-up, and analysed	11, figure 1
		(b) Give reasons for non-participation at each stage	11, Figure 1
		(c) Consider use of a flow diagram	Figure 1
Descriptive data	14*	(a) Give characteristics of study participants (eg demographic, clinical, social) and information on exposures and potential confounders	Table 1
		(b) Indicate number of participants with missing data for each variable of interest	Table 1
Outcome data	15*	Report numbers of outcome events or summary measures	11-13, figure 2, appendix 3
Main results	16	(*a*) Give unadjusted estimates and, if applicable, confounder-adjusted estimates and their precision (eg, 95% confidence interval). Make clear which confounders were adjusted for and why they were included	11-13, figure 2, appendix 3
		(*b*) Report category boundaries when continuous variables were categorized	12,13
		(*c*) If relevant, consider translating estimates of relative risk into absolute risk for a meaningful time period	n/a
Other analyses	17	Report other analyses done—eg analyses of subgroups and interactions, and sensitivity analyses	12,13, Appendix 3,
**Discussion**			
Key results	18	Summarise key results with reference to study objectives	14
Limitations	19	Discuss limitations of the study, taking into account sources of potential bias or imprecision. Discuss both direction and magnitude of any potential bias	14,15
Interpretation	20	Give a cautious overall interpretation of results considering objectives, limitations, multiplicity of analyses, results from similar studies, and other relevant evidence	15,16
Generalisability	21	Discuss the generalisability (external validity) of the study results	15,16,17
**Other information**			
Funding	22	Give the source of funding and the role of the funders for the present study and, if applicable, for the original study on which the present article is based	17,18

*Give information separately for cases and controls in case-control studies and, if applicable, for exposed and unexposed groups in cohort and cross-sectional studies.

Note: An Explanation and Elaboration article discusses each checklist item and gives methodological background and published examples of transparent reporting. The STROBE checklist is best used in conjunction with this article (freely available on the Web sites of PLoS Medicine at http://www.plosmedicine.org/, Annals of Internal Medicine at http://www.annals.org/, and Epidemiology at http://www.epidem.com/). Information on the STROBE Initiative is available at www.strobe-statement.org.

For peer review only

BMJ Open

Examining Patterns of Multimorbidity, Polypharmacy and Risk of Adverse Drug Reactions in Chronic Obstructive Pulmonary Disease: A Cross-Sectional UK Biobank Study

Journal:	*BMJ Open*
Manuscript ID	bmjopen-2017-018404.R2
Article Type:	Research
Date Submitted by the Author:	23-Nov-2017
Complete List of Authors:	Hanlon, Peter; University of Glasgow Institute of Health and Wellbeing<br>Nicholl, Barbara; University of Glasgow, General Practice and Primary Care<br>Jani, Bhautesh; University of Glasgow<br>McQueenie, Ross; University of Glasgow, GPPC, School of Medicine, Dentistry and Nursing, MVLS<br>Lee, Duncan; University of Glasgow<br>Gallacher, Katie I; University of Glasgow,<br>Mair, Frances; University of Glasgow, General Practice and Primary Care
**Primary Subject Heading**:	Respiratory medicine
Secondary Subject Heading:	General practice / Family practice
Keywords:	Multimorbidity, Polypharmacy, Chronic airways disease < THORACIC MEDICINE, Adverse events < THERAPEUTICS

SCHOLARONE™ Manuscripts

only

Examining Patterns of Multimorbidity, Polypharmacy and Risk of Adverse Drug Reactions in Chronic Obstructive Pulmonary Disease: A Cross-Sectional UK Biobank Study

Corresponding author

Professor Frances S Mair

General Practice and Primary Care, Institute of Health and Wellbeing, University of Glasgow, 1 Horelethill Road, Glasgow, G12 9LX, Scotland, United Kingdom

Tel: +44 141 330 8317

E-mail: frances.mair@glasgow.ac.uk

Authors:

Peter Hanlon[1], Barbara I. Nicholl[1], Bhautesh Dinesh Jani[1], Ross McQueenie[1], Duncan Lee[2], Katie I. Gallacher[1], Frances S Mair[1]

1. General Practice and Primary Care, Institute of Health and Wellbeing, University of Glasgow, 1 Horselethill Road, Glasgow, G12 9LX, Scotland, United Kingdom
2. School of Mathematics and Statistics, The mathematics and Statistics Building, University of Glasgow, University Place, Glasgow, G12 8SQ

Word count: 3446

For peer review only - http://bmjopen.bmj.com/site/about/guidelines.xhtml

Abstract

Objective: This study aims: (1) to describe the pattern and extent of multimorbidity and polypharmacy in UK Biobank participants with chronic obstructive pulmonary disease (COPD); and (2) to identify which comorbidities are associated with increased risk of adverse drug reactions (ADRs) resulting from polypharmacy.

Design: Cross-sectional.

Setting: Community cohort.

Participants: UK Biobank participants comparing self-reported COPD (n=8317) with no COPD (n=494,323).

Outcomes: Multimorbidity (≥ four conditions) and polypharmacy (≥ five medications) in participants with COPD versus those without. Risk of ADRs (taking ≥ three medications associated with falls, constipation, urinary retention, central nervous system (CNS) depression, bleeding or renal injury) in relation to the presence of COPD and individual comorbidities.

Results: Multimorbidity was more common in participants with COPD than those without (17% vs. 4%). Polypharmacy was highly prevalent (52% with COPD taking ≥ five medications vs 18% in those without COPD). Adjusting for age, sex and socioeconomic status, those with COPD were significantly more likely than those without to be prescribed ≥3 medications contributing to falls (Odds ratio (OR) 2.27, 95% confidence interval (CI) 2.13 to 2.42), constipation (OR 3.42, 95% CI 3.10 to 3.77), urinary retention (OR 3.38, 95% CI 2.94 to 3.87), CNS depression (OR: 3.75, 95% CI 3.31 to 4.25), bleeding (OR 4.61, 95% CI 3.35 to 6.19) and renal injury (OR 2.22, 95% CI 1.86 to 2.62). Concomitant cardiovascular disease was associated with the greatest risk of taking ≥3 medications associated with falls/renal injury. Concomitant mental health conditions were most strongly associated with medications linked with CNS depression/urinary retention/bleeding.

Conclusions: Multimorbidity is common in COPD and associated with high levels of polypharmacy. Co-prescription of drugs with various ADRs is common. Future research should examine the effects

For peer review only - http://bmjopen.bmj.com/site/about/guidelines.xhtml

on healthcare outcomes of co-prescribing multiple drugs with similar potential ADRs. Clinical guidelines should emphasise assessment of multimorbidity and ADR risk.

Abstract word count: 300

For peer review only

Strengths and Limitations

- This paper assesses multimorbidity, polypharmacy and risk of adverse drug reactions in UK Biobank participants with self-reported COPD compared with those without COPD.
- Baseline variables from the UK Biobank assessment centre were used to adjust for potential confounders.
- Cumulative risk of common adverse drug reactions was quantified by identifying UK Biobank participants taking three or more medications associated with similar adverse drug reactions.
- Analyses were repeated using a subgroup of participants with spirometry data confirming airflow obstruction.
- Medication and comorbidity data rely on participant self-report, and may thus be susceptible to bias or inaccuracy.

For peer review only - http://bmjopen.bmj.com/site/about/guidelines.xhtml

BACKGROUND

In people with Chronic Obstructive Pulmonary Disease (COPD), multimorbidity (the presence of two or more long-term conditions (LTCs)) is highly prevalent.(1-4) A recent meta-analysis of 29 datasets demonstrated that those with COPD are significantly more likely to be diagnosed with a range of cardiovascular comorbidities than those without COPD (we will use the term comorbidity when referring to specific conditions in addition to COPD, and multimorbidity to refer to the presence of two or more LTCs).(5) Other LTCs with known increased prevalence in COPD include obesity,(6) depression,(7-10) gastro-oesophageal reflux disease,(11-13) osteoporosis,(14-16) and lung cancer.(17, 18) Each of these conditions has been associated with poorer health related outcomes in COPD when compared to those with no comorbidity.(19-30) The overall burden of multimorbidity also impacts prognosis in COPD, for example higher number of comorbidities is associated with higher risk of mortality,(31) and higher burden of morbidity assessed using the Charlson index and the COPD-specific comorbidity test (COTE) is associated with higher risk of all-cause and respiratory specific mortality.(32, 33) The importance of considering the impact of multimorbidity in the management of long-term conditions is increasingly recognised, particularly in the context of an ageing society in which the prevalence of multimorbidity is growing.(34) However, an immature evidence base means that disease specific guidelines often lack specific recommendations with respect to multimorbidity.(35) The prevalence and prognostic significance of multimorbidity in COPD make it a potentially useful exemplar condition in which to consider the specific implications of different patterns of multimorbidity. Polypharmacy is one such implication.

Multimorbidity in the general population is associated with polypharmacy (often defined as concomitant use of ≥5 or ≥10 pharmacological agents).(36) Polypharmacy has been associated with increased risk of adverse drug reactions (ADRs)(37-39) and potentially preventable hospital admissions, particularly in the elderly.(40, 41) This is particularly pertinent in an ageing society, in

For peer review only - http://bmjopen.bmj.com/site/about/guidelines.xhtml

which the a rising prevalence of polypharmacy has been observed.(34, 37) It has been demonstrated that diagnosis of COPD is associated with increased risk of polypharmacy.(42, 43) This is, in large measure, due to the high burden of extra-pulmonary comorbidities.(44) However, little is known about the risk of ADRs in the context of multimorbidity in COPD.

Given the well-established burden of multimorbidity and polypharmacy in COPD, it is likely that those with COPD are at increased risk of ADRs resulting from polypharmacy. Previous analyses have not focused on the risk of specific ADRs, or assessed which LTCs increase this risk, instead reporting overall counts of prescribed medication. Data collected for the UK Biobank cohort offers an opportunity to assess how multimorbidity in COPD relates to polypharmacy and to assess the prevalence of co-prescription of medications with similar ADRs.

This paper aims:

- To describe the pattern and extent of multimorbidity and polypharmacy in UK Biobank participants with COPD.
- To identify which LTCs in people with COPD are associated with increased risk of ADRs resulting from polypharmacy.

For peer review only

METHODS

Data collection

The UK Biobank is a large, population cohort that recruited voluntary participants from throughout the United Kingdom. Between 2006 and 2010, UK Biobank recruited 502,640 participants aged 37 to 73. Participants underwent baseline assessments at one of 22 assessment centres throughout England, Scotland and Wales. Sociodemographic and lifestyle details were recorded using touchscreen questionnaires. Townsend scores were derived from participant postcodes to provide an area-based measure of socioeconomic deprivation. Self-reported LTCs, prescribed and over-the-counter medications, smoking status (current, previous or never) and frequency of alcohol intake (never / special occasions only, one-three times a month, at least once a week) were recorded from a touchscreen questionnaire and subsequent verbal interview with a study nurse. Physical activity was self-reported based on a questionnaire administered in the UK Biobank assessment centre.(45) We classified the responses into: none (no physical activity in the last four weeks), low (light 'DIY' activity only in the last four weeks), medium (heavy DIY and/or walking for pleasure and/or other exercises in the last four weeks), and high (strenuous sports in the last four weeks).

Study centre staff also collected physical measures including height and weight (to calculate body mass index (BMI)) and spirometry. Spirometry was performed using a Vitalograph Pneumotrac 6800. Individual reasons for contraindications to attempting spirometry were not recorded but, according to protocol, these included chest infection in the last month, history of collapsed lung, and heart attack or surgery in the past three months. Full details of the Biobank spirometry protocol are available at https://biobank.ctsu.ox.ac.uk/crystal/docs/Spirometry.pdf. In brief, participants were allowed up to three attempts to provide two reproducible spirometry measurements. Where the reproducibility of the first two was deemed acceptable (<5% variation in both FEV1 and FVC) a third measurement was not performed. All values were recorded along with any error messages generated. As per the American Thoracic Society/European Respiratory Society (ATS/ERS) end-of-

For peer review only - http://bmjopen.bmj.com/site/about/guidelines.xhtml

test criteria, we interpreted as valid any measurement with no error message or if 'user accepted' was specified.(46) No post-bronchodilator measurements were recorded, which deviates from the ATS/ERS guidelines and the Global Initiative for Chronic Obstructive Lung Disease (GOLD) guidelines for COPD.(47, 48)

Participants provided full informed consent to participate in UK Biobank and this study had full ethical approval from the NHS National Research Ethics Service for UK Biobank studies (Ref 16/NW/0274); this study is part of UK Biobank approved project number 14151.

Defining COPD

Participants reporting to have been diagnosed with chronic obstructive pulmonary disease, chronic bronchitis, or emphysema at the nurse-led interview were coded as having 'self-reported COPD'.

Due to the potential inaccuracies of using self-reported diagnoses, we identified a subset of those with self-reported COPD who met an adaptation of the Global Initiative for Obstructive Lung Disease (GOLD) spirometry criteria for COPD.(48) This subset, referred to as 'GOLD COPD', was used as a sensitivity analysis for self-reported COPD, and to stratify findings by severity of airflow obstruction. For participants with self-report COPD and valid spirometry measurements, we calculated the ratio of forced expiratory volume in 1 second (FEV1) to forced vital capacity (FVC) using the highest measurement for each participant meeting the American Thoracic Society/European Respiratory Society end-of-test criteria.(46) Those with a FEV1/FVC ratio <0.7 were classed as having an obstructive deficit and thus meeting the GOLD diagnostic criteria for COPD. We used the Hankinson equation,(49) based on recorded age, sex and height, to calculate predicted FEV1 values for each participant. Those with GOLD COPD were classified on the basis of their best available FEV1 measurement as having mild (>80% predicted FEV1), moderate (50-80% predicted FEV1), or severe (<50% predicted FEV1) airflow obstruction in line with the GOLD COPD guidelines.(48)

For peer review only - http://bmjopen.bmj.com/site/about/guidelines.xhtml

For peer review only

Defining long term conditions and medications

All LTCs were defined by self-report. The list of included LTCs was taken from a list of 42 morbidities originally established for a large multimorbidity epidemiological study in Scotland, through systematic review, the Quality and Outcomes Framework, NHS Scotland and an expert panel (34), and subsequently amended for UK Biobank (50). The inclusion of 'other painful conditions' comprised LTCs in which pain is a predominant feature (particularly as this is likely to influence medication use). It should be noted that such a list is not exhaustive, but intended to cover common conditions frequently requiring prescription of analgesics (e.g. osteoarthritis, back pain, headaches etc.). Morbidities were categorised for the purposes of this analysis into cardiovascular disease, gastrointestinal disease, mental health conditions, cancer, and painful conditions/inflammatory arthropathies (comprising the list of 'other painful conditions' mentioned above, plus connective tissue diseases). Full details of conditions comprising each category can be found in appendix 1.

Medication data were collected by self-report. Medications were coded by mechanism of action according to the British National Formulary (BNF) (e.g. Angiotensin-converting enzyme inhibitors, beta-blockers, calcium channel blockers, etc.).(51) For some situations where more than one medication with a similar mechanism of action may be commonly co-prescribed (e.g. aspirin and clopidogrel, both antiplatelets) these were coded separately. A complete list of the medications coded within each class can be found in appendix 2.

We defined those at risk of specific ADRs as anyone on 3 or more medications with similar potential ADRs, based on information provided in the *Scottish Government Model of Care Polypharmacy Working Group: Polypharmacy Guidance*.(52) This guideline cross-tabulates commonly prescribed medications with common ADRs to help identify those at cumulative risk of ADRs. This document groups common medications by similar potential ADRs. While this list is not all-inclusive, and the cut-

For peer review only - http://bmjopen.bmj.com/site/about/guidelines.xhtml

off value of three or more medications is arbitrary, this does allow an estimation of the cumulative risk of specific ADRs. We identified six potential ADRs (falls/fractures, constipation, urinary retention, CNS depression, bleeding and renal injury) for which the proportion of participants taking three or more associated medications could be assessed. It should be noted that several of these event (e.g. falls/fractures, CNS depression) are often multifactorial, and medication may be a contributing factor rather than a definitive cause. As the guideline acknowledges, however, these are clinical events of which the risk is increased by taking multiple associated medications.

For peer review only

Statistical analysis

All analyses were planned prior to inspection of the data.

Baseline variables

Comparisons were made between participants with self-reported COPD and the rest of the cohort (who did not report COPD). Age, sex, smoking status, deprivation (Townsend score), BMI, physical activity and frequency of alcohol intake were compared using χ^2 test for categorical variables, χ^2 test for trend for ordinal variables, and Mann-Whitney-U test for continuous variables. Total number of morbidities, prevalence of specific morbidities, number of self-reported prescribed medications, and proportion of participants taking each class of medication (Appendix 2), were also compared between those with self-reported COPD and the rest of the cohort. All comparisons were repeated comparing participants with GOLD COPD only with those without COPD, stratifying by severity of airflow obstruction.

Multimorbidity and polypharmacy

Logistic regression analyses were used to compare participants with self-reported COPD and those without COPD. Odds ratios (OR) and 95% confidence intervals (95% CI) were calculated for:

For peer review only - http://bmjopen.bmj.com/site/about/guidelines.xhtml

- the presence of cardiovascular disease, cancer, gastrointestinal disease, mental health conditions and painful conditions/inflammatory arthropathies
- the presence of four or more morbidities (excluding COPD)
- the use of five or more, and 10 or more, medications (two separate models)

Models were initially adjusted for age, sex and socioeconomic deprivation (model 1), then adjusted for the addition of smoking status, alcohol frequency, BMI and physical activity (model 2). These analyses were repeated comparing those with GOLD COPD only to those without COPD.

Risk of ADRs

For each potential ADR (falls/fractures, constipation, urinary retention, CNS depression, bleeding and renal injury) participants taking three or more medications associated with that ADR were identified. The following comparisons were then made:

- Unadjusted percentages at risk of each ADR were calculated for participants without COPD, with self-reported COPD, and with self-reported COPD plus each category of LTC (cardiovascular disease, cancer, gastrointestinal disease, mental health conditions and painful conditions/inflammatory arthropathies) to give an impression of the ADR risk in COPD, and identify LTCs in those with COPD that may increase this risk.
- ORs of being at risk of each ADR were calculated comparing those with self-reported COPD to those without COPD adjusting for age, sex and socioeconomic deprivation (model 1) and for age, sex, socioeconomic deprivation, smoking status, alcohol frequency, BMI and physical activity (model 2).
- ORs of being at risk of each ADR were calculated comparing those with and without self-reported COPD in each LTC category to (i.e. participants with cardiovascular disease alone compared to participants with cardiovascular disease plus COPD, etc.). This was intended to identify whether specific patterns of multimorbidity in COPD are associated with increased

For peer review only - http://bmjopen.bmj.com/site/about/guidelines.xhtml

ADR risk. Adjustment for a wide range of potential confounders was not appropriate in these models due to the smaller number of participants in each model.

Each analysis was repeated comparing GOLD COPD only to those without COPD. Less than 3% of participants (with or without COPD) had missing data for potential confounding variables (table 1). Those with missing data were excluded from adjusted analyses. Spirometry data were missing for 3591 participants with self-report COPD (43%), hence the use of the GOLD COPD subset as a sensitivity analysis.

All analyses were performed using R statistical software (version 3.3.1).

For peer review only

For peer review only - http://bmjopen.bmj.com/site/about/guidelines.xhtml

RESULTS

At the time of recruitment, 8317 out of 502,619 participants reported having COPD (1.7%) and are referred to here as the self-report COPD group. Of those who self-reported COPD, 4726 (57%) had valid spirometry measurements. Spirometry was contraindicated or not available in 2507 of those with self-reported COPD. Spirometry measurements did not meet the ATS/ESR end-of-test criteria in 1084 participants.(46) Of those with valid spirometry, 2620 (55%) met the GOLD criteria for airflow obstruction (399 (15%) mild, 1409 (54%) moderate, 812 (31%) severe, see Figure 1) and are referred to here as GOLD COPD.

Baseline variables

Table 1 describes and compares the characteristics of those with and without COPD in UK Biobank. Participants with COPD (both self-report and GOLD) were significantly older, more socioeconomically deprived, and less physically active. A higher proportion of those with COPD were male, obese and had a history of smoking.

For peer review only - http://bmjopen.bmj.com/site/about/guidelines.xhtml

Table 1. Baseline variables								
**Characteristic**	**No COPD n=494323**		**COPD (self-report) n=8317**			**GOLD GOPD n=2620**		
	Count	%	Count	%	p-value	Count	%	p-value
**Sex**								
Male	224906	45.5	4268	51.3		1426	54.4	
Female	269417	54.5	4049	48.7	<0.001	1194	45.6	<0.001
**Age**	Median: 58 IQR: 50-63		Median: 62 IQR: 57-66		<0.001	Median: 63 IQR: 59-66		<0.001
**Ethnicity**								
White	464770	94.5	8052	97.3		2620	100	
Other	26821	5.4	219	2.6	<0.001	0	0	<0.001
Missing	2732		46			0	0	
**Socioeconomic deprivation quintile**								
1 (least deprived)	99672	20.2	1015	12.2		309	11.8	
2	98977	20.0	1142	13.7		362	13.8	
3	99013	20.1	1399	16.8		440	16.8	
4	98660	20.0	1735	20.9		580	22.2	
5 (most deprived)	98385	19.7	3015	36.3	<0.001	926	35.4	<0.001
Missing	616		11			3		
**Smoking status**								
Current	50817	10.3	2172	26.3		833	32.2	
Previous	169015	34.4	4083	49.5		1398	54.0	
Never	271602	55.3	1999	24.2	<0.001	360	13.9	<0.001
Missing	2889		63			29		
**Alcohol frequency**								
Daily	100070	20.3	1720	20.7		618	23.6	
3-4 times/week	114058	23.1	1404	16.9		475	18.2	
1-2 times/week	127459	25.9	1863	22.5		561	21.5	
1-3 times/month	54979	11.2	894	10.8		289	11.1	
Occasional	56707	11.5	1322	15.9		387	14.8	<0.001
Never	39569	8.0	1092	13.2	<0.001	284	10.9	
Missing	1481		22			6		
**BMI**								
<18.5	2478	0.5	148	1.8		56	2.2	
18.5-24.9	155282	31.8	2185	26.8		829	31.9	
25.0-29.9	211102	43.2	3165	38.9		1049	40.4	
>30	119813	24.5	2647	32.5	<0.001	665	25.6	<0.001
Missing	5648		172			21		
**Physical activity**								
High	49827	10.6	250	3.1		70	2.7	
Medium	387766	79.6	5838	72.0		1902	73.3	
Low	18354	3.8	589	7.3		203	7.8	
None	31425	6.4	1433	17.7	<0.001	421	16.2	<0.001
Missing	6951		207			24		
**FEV1 (% predicted)**								
>80	272109	78.0	1853	39.2		399	15.2	
50-79	71727	20.6	2022	42.8		1409	53.8	
<50	4841	1.4	851	18.0	<0.001	812	31.0	<0.001
Missing	145646		3591			1061		

For peer review only

For peer review only - http://bmjopen.bmj.com/site/about/guidelines.xhtml

Multimorbidity and polypharmacy

Prevalence of each category of comorbidity was higher in those with COPD than without (table 2). After controlling for age, sex and socioeconomic status, those with self-reported COPD were significantly more likely than those without to have each category of LTC examined: cardiovascular disease (OR 1.45; 95% confidence interval (CI) 1.39 to 1.52) , cancer (1.29; 1.2 to 1.39), gastrointestinal disease (1.76; 1.67 to 1.86) , mental health conditions (2.02; 1.89 to 2.15) , and painful conditions (1.54; 1.46 to 1.62). Results for GOLD COPD also suggested higher likelihood of each LTC compared to those without COPD, although the ORs were lower and results for cancer not statistically significant (appendix 3). Results were similar after adjusting for additional confounders (smoking status, alcohol frequency, BMI and physical activity) with the exception of cardiovascular disease in GOLD COPD, which was no longer significantly associated (1.08; 0.99 to 1.18) (appendix 3).

For peer review only - http://bmjopen.bmj.com/site/about/guidelines.xhtml

Table 2: Long Term Conditions in those with and without COPD						
	Control	Self-report COPD	GOLD COPD			
	n=494323 count (%)	n=8317 count (%)	All n=2620 count (%)	Mild n=399 count (%)	Moderate n=1409 count (%)	Severe n= 812 count (%)
**Total comorbid-ities (excluding COPD)**						
≥4	19959 (4.0)	1389 (16.7)**	331 (12.6)**	46 (11.5)	191 (13.5)	94 (11.6)
**Total number of medications**						
≥1	356406 (72.1)	7670 (92.2)**	2452 (93.6)**	352 (88.2)	1321 (93.8)	779 (95.9)
≥5	87286 (17.7)	4312 (51.8)**	1349 (51.5)**	171 (42.9)	702 (49.8)	476 (58.6)
≥10	10678 (2.2)	1269 (15.3)**	329 (12.6)**	31 (7.8)	172 (12.2)	126 (15.5)
**Prevalence of comorbidities**						
**Cardiovascular**	**152891 (30.9)**	**3957 (47.6)****	**1156 (44.1)****	**142 (35.6)**	**611 (43.4)**	**403 (49.6)**
Hypertension	130119 (26.3)	3206 (38.5)**	916 (35.0)**	112 (28.1)	483 (34.3)	321 (39.5)
CHD	21560 (4.4)	1171 (14.1)**	315 (12.0)**	31 (7.6)	185 (13.1)	99 (12.2)
Diabetes	24737 (5.0)	766 (9.1)**	189 (7.2)**	16 (4.0)	109 (7.7)	64 (7.9)
Stroke/TIA	8459 (1.7)	395 (4.7)**	98 (3.7)**	11 (2.8)	51 (3.6)	36 (4.4)
AF	3552 (0.7)	99 (1.2)**	34 (1.3)**	3 (0.8)	16 (1.1)	15 (1.8)
Heart failure	768 (0.2)	35 (0.4)**	6 (0.2)	0	1 (0.1)	5 (0.6)
**Respiratory**						
Asthma	55245 (11.2)	3048 (36.6)**	984 (37.6)**	142 (35)	523 (37.1)	319 (39.3)
Pulmonary embolus	4354 (0.9)	264 (3.2)**	65 (2.5)**	12 (3.0)	32 (2.2)	21 (2.5)
Bronchiectasis	968 (0.2)	167 (2.0)**	39 (1.5)**	7 (1.8)	17 (1.2)	15 (1.8)
Pulmonary fib.	504 (0.1)	67 (0.8)**	18 (0.7)**	3 (0.8)	12 (0.9)	3 (0.4)
**Cancer**	**37686 (7.6)**	**937 (11.3)**	**272 (10.4)**	**47 (11.8)**	**146 (10.4)**	**79 (9.7)**
Lung	405 (0.1)	52 (0.6)**	15 (0.6)**	0	7 (0.5)	8 (1.0)
Breast	11311 (2.3)	210 (2.5)*	57 (2.2)	12 (3.0)	30 (2.1)	15 (1.8)
Prostate	3588 (0.7)	105 (1.3)**	30 (1.1)*	5 (1.3)	12 (0.9)	13 (1.6)
GI	2925 (0.6)	96 (1.2)**	34 (1.3)**	6 (1.5)	19 (1.3)	9 (1.1)
Haem	6170 (1.2)	124 (1.5)*	34 (1.3)	5 (1.3)	17 (1.2)	12 (1.5)
**Gastrointestinal**	**55635 (11.5)**	**1737 (20.9)****	**468 (17.9)****	**76 (19.0)**	**254 (18.0)**	**138 (17.0)**
Dyspepsia	37819 (7.7)	1257 (15.1)**	348 (13.3)**	53 (13.3)	189 (13.4)	106 (13.1)
Diverticular dis	5181 (1.0)	224 (2.7)**	54 (2.1)**	6 (1.5)	32 (2.3)	16 (2.0)
IBS	11203 (2.3)	291 (3.5)**	64 (2.4)**	17 (4.3)	35 (2.5)	12 (1.5)
CLD	935 (0.2)	36 (0.4)**	10 (0.4)*	2 (0.5)	10 (0.7)	3 (0.4)
**Mental Health**	**35822 (7.2)**	**1127 (13.6)****	**304 (11.6)****	**54 (13.5)**	**162 (11.5)**	**88 (10.8)**
Depression	27578 (5.6)	901 (10.8)**	233 (8.9)**	42 (10.5)	128 (9.1)	63 (7.8)
Anxiety	8781 (1.8)	245 (2.9)**	69 (2.6)**	13 (3.3)	36 (2.6)	20 (2.5)
Schizophrenia/ bipolar	1918 (0.4)	79 (0.9)**	27 (1.0)**	3 (0.7)	15 (1.1)	9 (1.1)
**Other**						
Other painful	81733 (16.5)	2259 (27.2)**	655 (25.0)**	115 (28.8)	367 (26.0)	173 (21.3)
Osteoporosis	7700 (1.6)	342 (4.1)**	128 (4.9)**	21 (5.3)	67 (4.8)	40 (4.9)
Connective tissue disease	10642 (2.2)	391 (4.7)**	112 (4.3)**	19 (4.8)	72 (5.1)	21 (2.6)

Compared with control (χ^2): * : p<0.05, ** : p<0.001

For peer review only - http://bmjopen.bmj.com/site/about/guidelines.xhtml

Morbidity counts (excluding COPD) and counts of prescribed medication are shown in table 2 comparing those with COPD, stratified by severity of airflow obstruction, with those without. Those with COPD had higher numbers of LTCs and more prescribed medications than those without. There was a trend towards more prescribed medications in those with greater severity of airway obstruction. After controlling for age, sex and socioeconomic status, those with self- report COPD were more likely to report ≥4 LTCs (3.49; 3.28 to 3.71), ≥5 medications (3.85; 3.68 to 4.03), and ≥10 medications (5.72; 5.36 to 6.10) than those without COPD. Results were similar for GOLD COPD and remained statistically significant after adjusting for smoking status, alcohol frequency, BMI and physical activity (appendix 3).

ADR Risk

Counts and percentages of participants taking specific medications are shown in appendix 4. Participants with COPD (self-report and GOLD) were more likely that those without COPD to be prescribed drugs across a range of disease areas, reflecting the range of LTCs present among those with COPD. The percentages of participants within each category (no COPD, COPD, and COPD with specific LTCs) taking three or more medications associated with a similar ADR is shown in Figure 2. For each category of ADR a higher proportion of participants with COPD reported taking three or more associated medications than those without COPD. This increased further with multimorbidity. Participants with COPD plus cardiovascular disease had the highest percentage taking three or more medications with a risk of falls or renal injury. Participants with COPD plus mental health conditions had the highest percentages taking three or more medications with a risk of constipation, CNS depression or bleeding.

After adjusting for age, sex and socioeconomic deprivation, those with self-report COPD remained more likely to be taking three or more medications in each category than those without COPD. These

For peer review only - http://bmjopen.bmj.com/site/about/guidelines.xhtml

findings remained statistically significant after adjusting for smoking status, alcohol frequency, BMI and physical activity (Table 3). Findings were similar for GOLD COPD however, after adjusting for additional potentially confounding variables, results for bleeding risk were not statistically significant in this sensitivity analysis (Table 3).

Table 3. Odds ratios (with 95% CI) for taking 3 of more medications associated with similar ADRs

ADR	Self-report COPD compared with no COPD N=502,640		GOLD COPD compared with no COPD N=496,943	
	Model 1 N=502,013	Model 2 N=487,718	Model 1 N=496,943	Model 2 N=482,378
	OR (95% CI)	OR (95% CI)	OR (95% CI)	OR (95% CI)
Falls	2.27 (2.13 – 2.42) ***	1.83 (1.71-1.96) ***	1.66 (1.47 – 1.87) ***	1.49 (1.30-1.69) ***
Constipation	2.71 (2.54 – 2.89) ***	2.66 (2.39-2.96) ***	2.18 (1.77 – 2.64) ***	1.82 (1.47-2.24) ***
Urinary retention	3.38 (2.94 – 3.87) ***	2.59 (2.22-3.0) ***	1.98 (1.44 – 2.64) ***	1.64 (1.18-2.21) **
CNS depression	3.75 (3.31 – 4.25) ***	2.81 (2.45-3.22) ***	2.29 (1.73 – 2.95) ***	1.87 (1.40-2.43) ***
Bleeding	4.60 (3.35 – 6.19) ***	3.39 (2.40-4.66) ***	2.63 (1.25 – 4.80) **	1.76 (0.75-3.48) §
Renal injury	2.22 (1.86 – 2.62) ***	1.84 (1.53-2.19) ***	1.94 (1.41 – 2.58) ***	1.84 (1.33-2.49) ***

§ : p>0.05 * : p<0.05, ** : p<0.01, *** : p<0.001
Model 1: Adjusted for age, sex and socioeconomic status
Model 2: Adjusted for age, sex, socioeconomic status, smoking, alcohol frequency, body mass index and physical activity

Finally, each category of ADR risk was assessed in a subgroup analysis for each category of LTC (cardiovascular, GI, cancer, mental health and painful conditions/inflammatory arthropathies) comparing those with and without COPD (e.g. participants with cardiovascular disease plus COPD compared with participants with cardiovascular disease alone, etc.). These models were adjusted for age, sex and socioeconomic status only. Within each category of LTC, those with self-reported COPD were more likely to be at risk of each ADR than those without COPD (appendix 3). Not all results were statistically significant when using GOLD COPD (Appendix 3).

For peer review only - http://bmjopen.bmj.com/site/about/guidelines.xhtml

DISCUSSION

Summary of main findings

Multimorbidity and polypharmacy in COPD were common among UK Biobank participants. The presence of multimorbidity was highly prevalent in those with COPD (85%). More than half (52%) reported polypharmacy (five or more medications), and 15% reported 10 or more medications. The prevalence of cardiovascular disease, as well as the degree of polypharmacy, was higher among those with more severe airflow obstruction.

For the first time, our data demonstrates that those with COPD were more likely than those without to be prescribed multiple medications (≥ three) with similar ADRs. Those with COPD plus cardiovascular disease were most likely to be taking multiple medications associated with increased risk of falls or renal injury, while those with COPD plus mental health conditions were most likely to be taking medications predisposing to constipation, CNS depression and bleeding.(52) Within each category of LTC, those with COPD were more likely to be taking multiple medications with similar ADRs than those without. These associations between patterns of multimorbidity and specific ADR risks have not been described or quantified previously.

Strengths and limitations

Strengths of this study include the large sample size with representation from different areas of the UK. The range of data collected at UK Biobank assessment centres meant it was possible to compare a range of sociodemographic characteristics as well as spirometry data, the latter being unusual for a large community based cohort. It is recognised, however, that UK Biobank participants show some evidence of 'healthy volunteer bias', differing from the UK average on a number of socioeconomic, lifestyle and health-related measures. Specifically they are less socioeconomically deprived, less likely to smoke, to be obese, and have fewer self-reported health conditions.(53) All LTC diagnoses

For peer review only - http://bmjopen.bmj.com/site/about/guidelines.xhtml

as well as medication data were self-reported, with no alternative means of verification. We attempted to minimise this limitation by identifying a subset of those with COPD meeting the GOLD diagnostic criteria and repeating the analyses with this subset. Importantly, spirometry values were also pre-bronchodilator, which is in contravention to guidelines for diagnosing COPD. Additionally, information was not available about the strength of indication for medications and individual susceptibility to risk, which is a limitation when considering the risk of ADRs.

The use of the Scottish Government Polypharmacy Guideline allowed analysis of potential ADR risk by specific common ADRs. The intended purpose of this guideline, however, was not to identify potential risk from a population sample, but rather to identify potential causes of symptoms or complications. The analysis in this study, therefore, serves only as an approximation of potential risk, not an absolute marker of inappropriate polypharmacy. The cross-sectional nature of this analysis also precludes an analysis of actual harm as a result of polypharmacy. Many of the potential ADRs, such as falls and fractures and renal injury, and frequently multifactorial events and may not be directly attributable to medication use. Despite these limitations, however, the co-prescription of multiple medications with similar ADRs strongly implies greater potential for harm. The association of such prescribing patterns with COPD, across a range of potential ADRs, is clear from our findings. This analysis is, to the author's knowledge, the first to attempt to quantify this risk for specific ADRs in this way.

For peer review only

Context and implications

The increased prevalence of individual LTCs such as coronary heart disease, hypertension, diabetes, dyspepsia, osteoporosis, cancer, depression and anxiety in those with COPD is similar to the findings from other population based studies of multimorbidity in COPD.(5, 11, 54-56) Our finding that cardiovascular disease prevalence increased with increasing severity of COPD is in keeping with the

For peer review only - http://bmjopen.bmj.com/site/about/guidelines.xhtml

body of literature on cardiovascular disease and COPD, in which high prevalence has been observed in (usually older) cohorts with severe airflow limitation.(5, 21) Greater polypharmacy with greater severity of COPD has also been observed previously in older COPD populations,(43, 57) although such analyses have been smaller (n=1859 and 398, respectively) and have not assessed the specific patterns of prescribing in COPD. To the best of our knowledge, no previous studies have assessed the risk of ADRs as a result of polypharmacy in COPD. A recent population-based analysis of prescribing data from 310,000 adults in Scotland showed that over 15 years from 1995 to 2010 the proportion of people with polypharmacy and with potentially serious drug-to-drug interactions increased dramatically.(37) The number of prescribed medications was also associated with increased risk of interactions. Our analysis differs in approach from this analysis, by seeking to identify patterns of prescribing increasing risk of specific adverse events, rather than counting total potential interactions. The strength of our approach lies in highlighting specific patterns of multimorbidity in which specific ADRs are more likely. Our findings can therefore be applied to clinical practice, highlighting the importance of recognising multimorbidity in COPD and being alert to specific ADRs when prescribing medication.

Our findings indicate that in those with COPD the potential for ADRs as a result of combinations of medications is high, and this appears to be the result of a high prevalence of extra-pulmonary LTCs. Clinical guidelines for COPD should place greater emphasis on the need for assessment of associated multimorbidity and the risk of associated ADRs. While our analysis shows potential areas where ADR risk exists in COPD (e.g. falls in those with concomitant cardiovascular disease, CNS depression, constipation with concomitant mental health conditions), future research is merited to assess what actual harm could be attributed to such prescribing patterns.

Conclusion

For peer review only - http://bmjopen.bmj.com/site/about/guidelines.xhtml

Among UK Biobank participants with COPD there was considerable multimorbidity and polypharmacy. Those with COPD were highly likely to be concurrently prescribed multiple medications with similar potential adverse effects. Medications contributing to this risk were largely indicated for the management of the associated morbidities rather than COPD. Future research should examine the effects on healthcare outcomes of co-prescribing of multiple drugs with similar potential of ADRs. Clinical guidelines for COPD should emphasise the need for assessment of multimorbidity and the risk of associated ADRs.

Figure Legends

Figure 1: Flow diagram of identification of participants with 'self-report COPD' and 'GOLD COPD'.

Figure 2: Bubble plot showing percentage of participants in each comorbidity category taking 3 or more concomitant medications associated with specific adverse drug reactions.

Ethics approval and consent to participate

Participants provided full informed consent to participate in UK Biobank and this study was covered by the generic ethical approval for UK Biobank studies from the NHS National Research Ethics Service (Ref 16/NW/0274).

Availability of data and materials

UK Biobank data is available via *www.ukbiobank.ac.uk*. Syntax for the generation of derived variables and for the analysis used for this study will be submitted to UK Biobank for record.

For peer review only - http://bmjopen.bmj.com/site/about/guidelines.xhtml

Competing interests

The authors declare that they have no competing interests

Funding

This study was supported by a CSO Catalyst Grant CGA/16/39. BDJ was funded by a NHS Research for Scotland career research fellowship.

Author contributions

All authors (PH, BN, BJ, RM, DL, KG and FM) were involved in the conceptualisation and design of the project and interpretation of results. PH carried out the analysis with support from BJ, RM and BN. DL provided statistical support. All authors had access to the data. PH wrote the first draft of the paper and all authors commented on subsequent drafts. All authors approved the final draft for publication. FM is guarantor.

Acknowledgements

This research has been conducted using the UK Biobank Resource, approved project number 14151.

References

For peer review only - http://bmjopen.bmj.com/site/about/guidelines.xhtml

1. Anecchino C, Rossi E, Fanizza C, et al. Prevalence of chronic obstructive pulmonary disease and pattern of comorbidities in a general population. International journal of chronic obstructive pulmonary disease. 2007;2(4):567-74.
2. Fumagalli G, Fabiani F, Forte S, et al. INDACO project: a pilot study on incidence of comorbidities in COPD patients referred to pneumology units. Multidisciplinary Respiratory Medicine. 2013;8.
3. Putcha N, Puhan MA, Hansel NN, et al. Impact of co-morbidities on self-rated health in self-reported COPD: An analysis of NHANES 2001-2008. Copd-Journal of Chronic Obstructive Pulmonary Disease. 2013;10(3):324-32.
4. Garcia-Olmos L, Alberquilla A, Ayala V, et al. Comorbidity in patients with chronic obstructive pulmonary disease in family practice: a cross sectional study. BMC family practice. 2013;14.
5. Chen W, Thomas J, Sadatsafavi M, et al. Risk of cardiovascular comorbidity in patients with chronic obstructive pulmonary disease: a systematic review and meta-analysis. Lancet Respir Med. 2015;3(8):631-9.
6. Rodriguez DA, Garcia-Aymerich J, Valera JL, et al. Determinants of exercise capacity in obese and non-obese COPD patients. Respiratory Medicine. 2014;108(5):745-51.
7. Al-shair K, Dockry R, Mallia-Milanes B, et al. Depression and its relationship with poor exercise capacity, BODE index and muscle wasting in COPD. Respiratory Medicine. 2009;103(10):1572-9.
8. Di Marco F, Verga M, Reggente M, et al. Anxiety and depression in COPD patients: The roles of gender and disease severity. Respiratory Medicine. 2006;100(10):1767-74.
9. Qian J, Simoni-Wastila L, Rattinger GB, et al. Associations of depression diagnosis and antidepressant treatment with mortality among young and disabled Medicare beneficiaries with COPD. General Hospital Psychiatry. 2013;35(6):612-8.
10. Atlantis E, Fahey P, Cochrane B, et al. Bidirectional Associations Between Clinically Relevant Depression or Anxiety and COPD A Systematic Review and Meta-analysis. Chest. 2013;144(3):766-77.
11. Bor S, Kitapcioglu G, Solak ZA, et al. Prevalence of gastroesophageal reflux disease in patients with asthma and chronic obstructive pulmonary disease. Journal of Gastroenterology and Hepatology. 2010;25(2):309-13.
12. Garcia Rodriguez LA, Ruigomez A, Martin-Merino E, et al. Relationship Between Gastroesophageal Reflux Disease and COPD in UK Primary Care. Chest. 2008;134(6):1223-30.
13. Kim J, Lee JH, Kim Y, et al. Association between chronic obstructive pulmonary disease and gastroesophageal reflux disease: a national cross-sectional cohort study. Bmc Pulmonary Medicine. 2013;13.
14. Ferguson GT, Calverley PMA, Anderson JA, et al. Prevalence and Progression of Osteoporosis in Patients With COPD Results From the Towards a Revolution in COPD Health Study. Chest. 2009;136(6):1456-65.
15. Jorgensen NR, Schwarz P, Holme I, et al. The prevalence of osteoporosis in patients with chronic obstructive pulmonary disease - A cross sectional study. Respiratory Medicine. 2007;101(1):177-85.
16. Ogura-Tomomatsu H, Asano K, Tomomatsu K, et al. Predictors of Osteoporosis and Vertebral Fractures in Patients Presenting with Moderate-to-Severe Chronic Obstructive Lung Disease. Copd-Journal of Chronic Obstructive Pulmonary Disease. 2012;9(4):332-7.
17. Young RP, Hopkins RJ, Christmas T, et al. COPD prevalence is increased in lung cancer, independent of age, sex and smoking history. European Respiratory Journal. 2009;34(2):380-6.
18. Wilson DO, Weissfeld JL, Balkan A, et al. Association of radiographic emphysema and airflow obstruction with lung cancer. American Journal of Respiratory and Critical Care Medicine. 2008;178(7):738-44.
19. Garcia-Rio F, Soriano JB, Miravitlles M, et al. Impact of Obesity on the Clinical Profile of a Population-Based Sample with Chronic Obstructive Pulmonary Disease. Plos One. 2014;9(8).

For peer review only - http://bmjopen.bmj.com/site/about/guidelines.xhtml

20. Cecere LM, Littman AJ, Slatore CG, et al. Obesity and COPD: Associated Symptoms, Health-related Quality of Life, and Medication Use. Copd-Journal of Chronic Obstructive Pulmonary Disease. 2011;8(4):275-84.
21. Mannino DM, Thorn D, Swensen A, et al. Prevalence and outcomes of diabetes, hypertension and cardiovascular disease in COPD. European Respiratory Journal. 2008;32(4):962-9.
22. Curkendall SM, DeLuise C, Jones JK, et al. Cardiovascular disease in patients with chronic obstructive pulmonary disease, Saskatchewan Canada cardiovascular disease in COPD patients. Annals of Epidemiology. 2006;16(1):63-70.
23. McGarvey LP, John M, Anderson JA, et al. Ascertainment of cause-specific mortality in COPD: operations of the TORCH Clinical Endpoint Committee. Thorax. 2007;62(5):411-5.
24. Zielinski J, MacNee W, Wedzicha J, et al. Causes of death in patients with COPD and chronic respiratory failure. Monaldi archives for chest disease = Archivio Monaldi per le malattie del torace. 1997;52(1):43-7.
25. Mannino DM, Doherty DE, Buist AS. Global Initiative on Obstructive Lung Disease (GOLD) classification of lung disease and mortality: findings from the Atherosclerosis Risk in Communities (ARIC) study. Respiratory Medicine. 2006;100(1):115-22.
26. Campo G, Guastaroba P, Marzocchi A, et al. Impact of COPD on Long-term Outcome After ST-Segment Elevation Myocardial Infarction Receiving Primary Percutaneous Coronary Intervention. Chest. 2013;144(3):750-7.
27. Konecny T, Somers K, Orban M, et al. Interactions Between COPD and Outcomes After Percutaneous Coronary Intervention. Chest. 2010;138(3):621-7.
28. Sidney S, Sorel M, Quesenberry CP, et al. COPD and incident cardiovascular disease hospitalizations and mortality: Kaiser permanente medical care program. Chest. 2005;128(4):2068-75.
29. Sakae TM, Menezes Pizzichini MM, et al. Exacerbations of COPD and symptoms of gastroesophageal reflux: a systematic review and meta-analysis. Jornal Brasileiro De Pneumologia. 2013;39(3):259-71.
30. Hattiholi J, Gaude GS. Prevalence and correlates of osteoporosis in chronic obstructive pulmonary disease patients in India. Lung India : official organ of Indian Chest Society. 2014;31(3):221-7.
31. Miller J, Edwards LD, Agusti A, et al. Comorbidity, systemic inflammation and outcomes in the ECLIPSE cohort. Respiratory Medicine. 2013;107(9):1376-84.
32. Divo M, Cote C, de Torres JP, et al. Comorbidities and Risk of Mortality in Patients with Chronic Obstructive Pulmonary Disease. American Journal of Respiratory and Critical Care Medicine. 2012;186(2):155-61.
33. Budweiser S, Harlacher M, Pfeifer M. Co-morbidities and hyperinflation are independent risk factors of all-cause mortality in very severe COPD. COPD. 2014;11(4):388-400.
34. Barnett K, Mercer SW, Norbury M, et al. Epidemiology of Multimorbidity and Implications for Health Care, Research, and Medical Education: a Cross-Sectional Study. Lancet. 2012;380(9836):37-43.
35. National Institute for Health and Care Excellence (NICE). Multimorbidity: clinical assessment and management (NICE Guideline 56). 2016. https://www.nice.org.uk/guidance/ng56 (accessed Nov 2017)
36. Corsonello A, Pedone C, Corica F. Polypharmacy in elderly patients at discharge from the acute care hospital. Therapeutics and Clinical Risk Management. 2007;2007(3):1.
37. Guthrie B, Makubate B, Hernandez-Santiago V, et al. The rising tide of polypharmacy and drug-drug interactions: population database analysis 1995-2010. BMC Medicine. 2015;13:74.
38. Bourgeois FT, Shannon MW, Valim C, et al. Adverse drug events in the outpatient setting: an 11-year national analysis. Pharmacoepidemiol Drug Saf. 2010;19(9):901-10.

For peer review only

For peer review only - http://bmjopen.bmj.com/site/about/guidelines.xhtml

39. Johnell K, Klarin I. The relationship between number of drugs and potential drug-drug interactions in the elderly: a study of over 600,000 elderly patients from the Swedish Prescribed Drug Register. Drug Saf. 2007;30(10):911-8.
40. Pirmohamed M, James S, Meakin S, et al. Adverse drug reactions as cause of admission to hospital: prospective analysis of 18 820 patients. BMJ. 2004;329(7456):15-9.
41. Howard RL, Avery AJ, Slavenburg S, et al. Which drugs cause preventable admissions to hospital? A systematic review. British Journal of Clinical Pharmacology. 2007;63(2):136-47.
42. Nobili A, Marengoni A, Tettamanti M, et al. Association between clusters of diseases and polypharmacy in hospitalized elderly patients: results from the REPOSI study. European Journal of Internal Medicine. 2011;22(6):597-602.
43. Franssen FM, Spruit MA, Wouters EF. Determinants of polypharmacy and compliance with GOLD guidelines in patients with chronic obstructive pulmonary disease. Int J Chron Obstruct Pulmon Dis. 2011;6:493-501.
44. Patel A. Extrapulmonary Polypharmacy and Cardiovascular Medications in COPD. Thorax. 2009;64(Suppl IV):A5-A74.
45. UK Biobank data field 6164 [Available from: http://biobank.ctsu.ox.ac.uk/crystal/field.cgi?id=6164] (accessed Nov 2017)
46. Miller MR, Hankinson J, Brusasco V, B et al. Standardisation of spirometry. European Respiratory Journal. 2005;26(2):319-38.
47. Qaseem A, Wilt TJ, Weinberger SE, et al. Diagnosis and management of stable chronic obstructive pulmonary disease: a clinical practice guideline update from the American College of Physicians, American College of Chest Physicians, American Thoracic Society, and European Respiratory Society. Annals of internal medicine. 2011;155(3):179-91.
48. Global Initiative for Chronic Obstructive Lung Disease (GOLD). From the Global Strategy for the Diagnosis, Management and Prevention of COPD, Global Initiative for Chronic Obstructive Lung Disease (GOLD) 2017 2017 [Available from: Available from: http://goldcopd.org. (accessed Nov 2017)
49. Hankinson JL, Odencrantz JR, Fedan KB. Spirometric reference values from a sample of the general U.S. population. American Journal of Respiratory & Critical Care Medicine. 1999;159(1):179-87.
50. Nicholl BI, Mackay D, Cullen B, et al. Chronic multisite pain in major depression and bipolar disorder: cross-sectional study of 149,611 participants in UK Biobank. BMC Psychiatry. 2014;14:350.
51. British National Formulary Version 70: BMJ Group and Royal Pharmaceutical Society of Great Britain; 2015.
52. Scottish Government Model of Care Polypharmacy Working Group. Polypharmacy Guidance (2nd edition). March 2015. Scottish Government
53. Fry A, Littlejohns TJ, Sudlow C, et al. The representativeness of the UK Biobank cohort on a range of sociodemographic, physical, lifestyle and health-related characteristics. . Journal of epidemiology and community health. 2016;70(Suppl 1):A26-A.
54. Putcha N, Han MK, Martinez CH, et al. Comorbidities of COPD have a major impact on clinical outcomes, particularly in African Americans. Chronic obstructive pulmonary diseases (Miami, Fla). 2014;1(1):105-14.
55. Bhattacharyya P, Paul R, Ghosh M, et al. Prevalence of osteoporosis and osteopenia in advanced chronic obstructive pulmonary disease patients. Lung India : official organ of Indian Chest Society. 2011;28(3):184-6.
56. de Torres JP, Bastarrika G, Wisnivesky JP, et al. Assessing the relationship between lung cancer risk and emphysema detected on low-dose CT of the chest. Chest. 2007;132(6):1932-8.
57. Diez-Manglano J, Barquero-Romero J, Mena PA, et al. Polypharmacy in patients hospitalised for acute exacerbation of COPD. European Respiratory Journal. 2014;44(3):791-4.

For peer review only - http://bmjopen.bmj.com/site/about/guidelines.xhtml

For peer review only

For peer review only - http://bmjopen.bmj.com/site/about/guidelines.xhtml

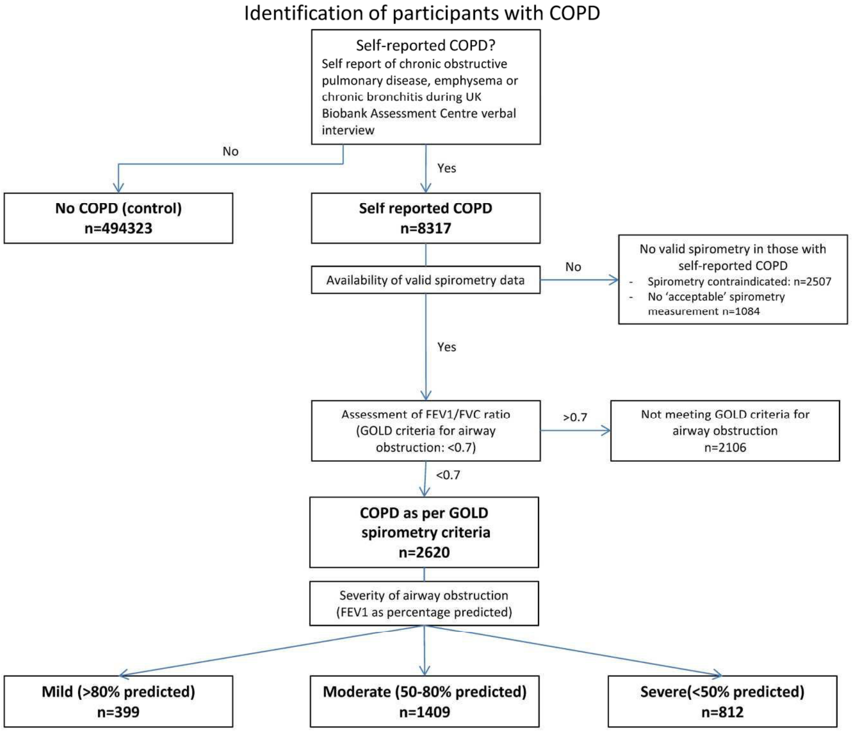

Flow diagram of identification of participants with 'self-report COPD' and 'GOLD COPD'.

190x142mm (300 x 300 DPI)

For peer review only

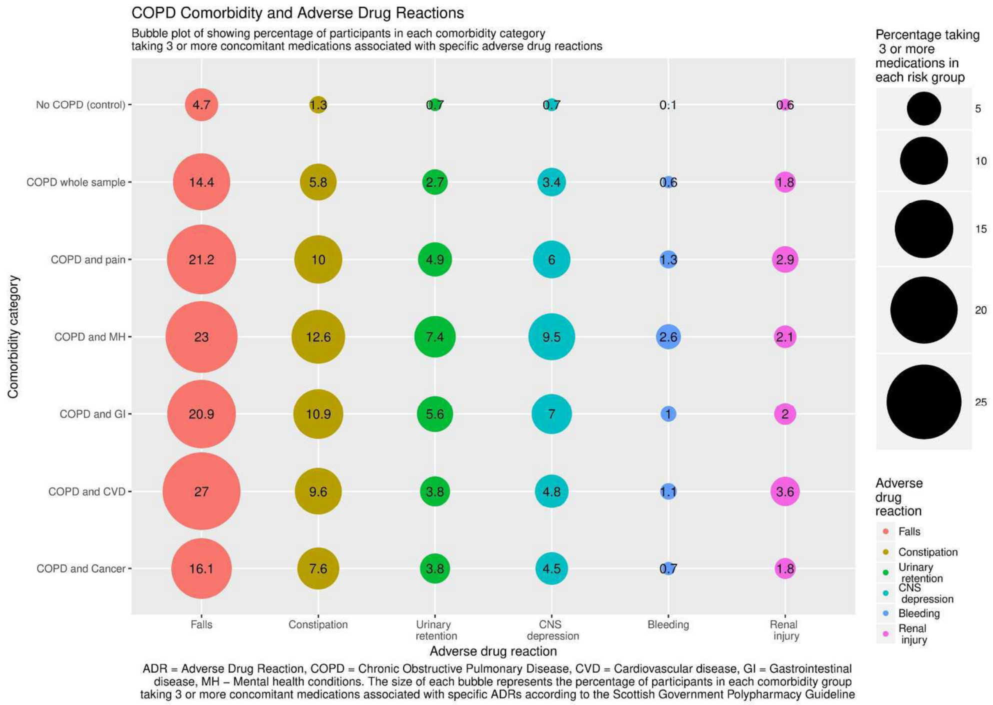

Bubble plot showing percentage of participants in each comorbidity category taking 3 or more concomitant medications associated with specific adverse drug reactions.

209x148mm (300 x 300 DPI)

**Comorbidity category (used in analysis)**	**Conditions included (as reported in table 2)**	**Self-reported conditions comprising this condition (UK Biobank variables used to identify self-reported conditions)**
Cardiovascular conditions	Hypertension	Hypertension<br>Essential hypertension
	Coronary heart disease	Heart attack/MI<br>Angina
	Diabetes	Diabetic nephropathy<br>Diabetic neuropathy/ulcers<br>Diabetes<br>Type 1 diabetes<br>Type 2 diabetes<br>Diabetic eye disease
	Stroke/TIA	Stroke<br>TIA<br>Subarachnoid haemorrhage<br>Brain haemorrhage<br>Ischaemic stroke
	Atrial fibrillation	Atrial fibrillation
	Heart failure	Cardiomyopathy<br>Hypertrophic cardiomyopathy<br>Heart failure/pulmonary oedema
	Peripheral vascular disease	Peripheral vascular disease<br>Leg claudication/intermittent claudication
Respiratory	COPD	COPD/Chronic obstructive pulmonary disease<br>Emphysema/Chronic bronchitis<br>Emphysema
	Asthma	Asthma
	PE/DVT	Deep vein thrombosis<br>Pulmonary emolism
	Bronchiectasis	Bronchiectasis
	Pulmonary fibrosis	Pulmonary fibrosis
Cancer	Cancer	"yes"/"no" to "have you ever had cancer?"
Gastrointestinal	Dyspepsia	Gastro-oesophageal reflux (GORD)<br>Oesophagitis/Barrett's oesophagus<br>Gastric stomach ulcers<br>Gastric erosions/gastritis<br>Duodenal ulcer<br>Dyspepsia/indigestion<br>Hiatus hernia<br>Helicobacter pylori
	Diverticular disease	Diverticular disease/diverticulitis

For peer review only

	Irritable bowel syndrome	Irritable bowel syndrome
	Chronic liver disease	Oesophageal varices<br>Non infective hepatitis<br>Liver failure/cirrhosis<br>Primary biliary cirrhosis
	Inflammatory bowel disease	Inflammatory bowel disease<br>Crohn's disease<br>Ulcerative colitis
	Constipation	Constipation
	Viral hepatitis	Hepatitis B<br>Hepatitis C<br>Hepatitis D
Mental Health	Depression	Depression<br>Postnatal depression
	Anxiety	Anxiety/panic attacks<br>Nervous breakdown<br>Post-traumatic stress disorder<br>Obsessive compulsive disorder<br>Stress<br>Insomnia<br>Psychological/psychiatric problem
	Schizophrenia	Scizophrenia
	Bipolar	Mania<br>Bipolar disorder<br>Manic depression
Painful conditions	Connective tissue diseases	Myositis/myopathy<br>Systemic lupus erythematosus/SLE<br>Connective tissue disorder<br>Sjogren's syndrome.sicca syndrome<br>Dermatopolymyositis<br>Scloeroderma/systemic sclerosis<br>Rheumatoid arthritis<br>Psoriatic arthropathy<br>Dermatomyositis<br>Polymyositis<br>Polymyalgia rheumatica
	Other painful conditions	Back pain<br>Joint pain<br>Headaches (not migraine)<br>Sciatica<br>Plantar fasciitis<br>Carpal tunnel syndrome<br>Fibromyalgia<br>Arthritis<br>Shingles<br>Disc problem<br>Prolapsed disc/slipped disc

For peer review only

		Spine arthritis/spondylitis<br>Ankylosing spondylitis<br>Back problem<br>Osteoarthritis<br>Gout<br>Cervical spondylosis<br>Trigeminal neuralgia<br>Disc degeneration<br>Trapped nerve/compressed nerve
Other	Osteoporosis	Osteoporosis

For peer review only

Drugs with cumulative risk of Adverse Drug Reactions*	
Adverse Drug Reaction	Contributing drug classes (participants taking 3 or more considered 'at risk' for the purposes of analysis)
Falls	H2-receptor blockers<br>Loperamide<br>Prochlorperazine<br>Metoclopramide<br>ACE-inhibitor/Angiotensin receptor blocker<br>Thiazide diuretic<br>Loop diuretic<br>Amiloride/triamterene<br>Spironolactone<br>Beta-blocker<br>Calcium-channel blocker<br>Nitrates or nicorandil<br>Digoxin<br>Oral steroids<br>Opiates<br>Benzodiazepines<br>Sedative antihistamines<br>H1-receptor blockers<br>Antipsychotics<br>Tricyclic antidepressants<br>Sulfonylureas/gliptins/glinides<br>Pioglitazone<br>Urinary antispasmodics<br>Dosulepin<br>Alpha-blockers
Constipation	H2-receptor blockers<br>Laxatives<br>Loperamide<br>Prochlorperazine<br>Thiazide diuretics<br>Loop diuretics<br>Calcium-channel blockers<br>Opiates<br>Sedative antihistamines<br>H1-receptor blockers<br>Antipsychotics<br>Tricyclic antidepressants<br>Urinary antispasmodics<br>Dosulepin
Urinary retention	H2-receptor blockers<br>Loperamide<br>Prochlorperazine<br>Opiates<br>Sedative antihistamines<br>H1-receptor blockers<br>Antipsychotics<br>Tricyclic antidepressants

For peer review only

	Urinary antispasmodics<br>Dosulepin
CNS depression	H2-receptor blockers<br>Loperamide<br>Prochlorperazine<br>Oral steroids<br>Opiates<br>Benzodiazepines<br>Sedative antihistamines<br>H1-receptor blockers<br>Antipsychotics<br>Tricyclic antidepressants<br>Urinary antispasmodics<br>Dosulepin
Bleeding	Aspirin<br>Clopidogrel<br>Other antiplatelets<br>Oral steroids<br>SSRIs and related drugs<br>Non-steroidal anti-inflammatory drugs<br>Warfarin
Renal injury	ACE-inhibitor/angiotensin receptor blockers<br>Thiazide diuretic<br>Loop diuretic<br>Amiloride/triamterene<br>Spironolactone<br>Antibiotics/antifungals<br>Non-steroidal anti-inflammatory drugs
Adapted from *Scottish Government Model of Care Polypharmacy Working Group. Polypharmacy Guidance (2nd edition) March 2015. Scottish Government.*	

For peer review only

Table S1. Odds ratios (with 95% CI) for the presence of categories of comorbidity

Comorbidity category	Self-report COPD compared with no COPD N=502,640		GOLD COPD compared with no COPD N=496,943	
	Model 1 N=502,013	Model 2 N=487,718	Model 1 N=496,324	Model 2 N=482,378
	OR (95% CI)	OR (95% CI)	OR (95% CI)	OR (95% CI)
Cardiovascular disease	1.45 (1.39-1.52) ***	1.28 (1.22-1.34) ***	1.11 (1.02-1.20) *	1.08 (0.99-1.18) §
Cancer	1.29 (1.20-1.39) ***	1.22 (1.13-1.31) ***	1.12 (0.99-1.27) §	1.06 (0.92-1.19) §
Gastrointestinal disease	1.76 (1.67-1.86) ***	1.56 (1.48-1.65) ***	1.4 (1.26-1.54) ***	1.24 (1.12-1.38) ***
Mental health	2.02 (1.89-2.15) ***	1.62 (1.51-1.73) ***	1.75 (1.54-1.97) ***	1.40 (1.22-1.58) ***
Painful conditions	1.62 (1.55-1.70) ***	1.41 (1.34-1.48) ***	1.40 (1.28-1.52) ***	1.24 (1.13-1.35) **

§ : p>0.05 * : p<0.05, ** : p<0.01, *** : p<0.001
Model 1: Adjusted for age, sex and socioeconomic status
Model 2: Adjusted for age, sex, socioeconomic status, smoking, alcohol frequency, body mass index and physical activity

Table S2. Odds ratios (with 95% CI) for the presence of multimorbidity or polypharmacy

Outcome	Self-report COPD compared with no COPD N=502,640		GOLD COPD compared with no COPD N=496,943	
	Model 1 N=502,013	Model 2 N=487,718	Model 1 N=496,324	Model 2 N=482,378
	OR (95% CI)	OR (95% CI)	OR (95% CI)	OR (95% CI)
Multimorbidity (≥4 conditions)	3.49 (3.28-3.70) ***	2.79 (2.61-2.98) ***	2.34 (2.10-2.63) ***	1.99 (1.75-2.25) ***
Polypharmacy (≥5 medications)	3.85 (3.68-4.03) ***	3.30 (3.15-3.46) ***	3.47 (3.20-3.75) ***	3.20 (2.95-3.48) ***
Polypharmacy (≥10 medications	5.72 (5.36-6.10) ***	4.42 (4.11-4.75) ***	4.20 (3.72-4.73) ***	3.56 (3.12-4.05) ***

§ : p>0.05 * : p<0.05, ** : p<0.01, *** : p<0.001
Model 1: Adjusted for age, sex and socioeconomic status
Model 2: Adjusted for age, sex, socioeconomic status, smoking, alcohol frequency, body mass index and physical activity

Table S3. Odds ratios (with 95% CI) for taking 3 of more medications associated with similar ADRs

ADR	Self-report COPD compared with no COPD N=502,640		GOLD COPD compared with no COPD N=496,943	
	Model 1 N=502,013	Model 2 N=487,718	Model 1 N=496,943	Model 2 N=482,378
	OR (95% CI)	OR (95% CI)	OR (95% CI)	OR (95% CI)
Falls	2.27 (2.13 – 2.42) ***	1.83 (1.71-1.96) ***	1.66 (1.47 – 1.87) ***	1.49 (1.30-1.69) ***
Constipation	2.71 (2.54 – 2.89) ***	2.66 (2.39-2.96) ***	2.18 (1.77 – 2.64) ***	1.82 (1.47-2.24) ***
Urinary retention	3.38 (2.94 – 3.87) ***	2.59 (2.22-3.0) ***	1.98 (1.44 – 2.64) ***	1.64 (1.18-2.21) **
CNS Depression	3.75 (3.31 – 4.25) ***	2.81 (2.45-3.22) ***	2.29 (1.73 – 2.95) ***	1.87 (1.40-2.43) ***
Bleeding	4.60 (3.35 – 6.19) ***	3.39 (2.40-4.66) ***	2.63 (1.25 – 4.80) **	1.76 (0.75-3.48) §
Renal injury	2.22 (1.86 – 2.62) ***	1.84 (1.53-2.19) ***	1.94 (1.41 – 2.58) ***	1.84 (1.33-2.49) ***

§ : p>0.05 * : p<0.05, ** : p<0.01, *** : p<0.001
Model 1: Adjusted for age, sex and socioeconomic status
Model 2: Adjusted for age, sex, socioeconomic status, smoking, alcohol frequency, body mass index and physical activity

For peer review only

Subgroup analyses – comparing COPD with no COPD among participants with specific categories of comorbidity

Table S4. Odds ratios (with 95% CI) for taking 3 of more medications associated with similar ADRs in participants with cardiovascular disease (CVD)

ADR	Self-report COPD plus CVD compared with CVD alone (no COPD) N=156,848	GOLD COPD plus CVD compared with CVD alone (no COPD) N=154,047
	Model 1 N=156,667	Model 1 N=153,852
	OR (95% CI)	OR (95% CI)
Falls	1.92 (1.79-2.07) ***	1.59 (1.39-1.82) ***
Constipation	2.89 (2.58-3.23) ***	2.06 (1.63-2.57) ***
Urinary retention	2.78 (2.33-3.28) ***	1.92 (1.30-2.72) ***
CNS Depression	3.17 (2.71-3.69) ***	2.17 (1.54-2.97) ***
Bleeding	4.00 (2.85-5.48) ***	2.26 (0.96-4.44) *
Renal injury	1.90 (1.59-2.25) ***	1.82 (1.31-2.45) ***

§ : p>0.05 * : p<0.05, ** : p<0.01, *** : p<0.001
Model 1: Adjusted for age, sex and socioeconomic status

Table S5. Odds ratios (with 95% CI) for taking 3 of more medications associated with similar ADRs in participants with cancer

ADR	Self-report COPD plus cancer compared with cancer alone (no COPD) N=38,623	GOLD COPD plus cancer compared with cancer alone (no COPD) N=37,958
	Model 1 N=38,575	Model 1 N= 37,912
	OR (95% CI)	OR (95% CI)
Falls	2.35 (1.95-2.81) ***	1.49 (1.00-2.13) *
Constipation	3.55 (2.73-4.56) ***	2.21 (1.22-3.68) **
Urinary retention	3.65 (2.52-5.13) ***	1.99 (0.78-4.14) §
CNS Depression	3.74 (2.66-5.14) ***	2.04 (0.86-4.04) §
Bleeding	4.69 (1.91-9.86) ***	2.20 (0.12-10.23) §
Renal injury	2.0 (1.17-3.20) **	2.26 (0.89-4.71) §

§ : p>0.05 * : p<0.05, ** : p<0.01, *** : p<0.001
Model 1: Adjusted for age, sex and socioeconomic status

Table S6. Odds ratios (with 95% CI) for taking 3 of more medications associated with similar ADRs in participants with gastrointestinal disease (GI)

ADR	Self-report COPD plus GI compared with GI alone (no COPD) N=58372	GOLD COPD plus GI compared with GI alone (no COPD) N=57103
	Model 1 N=58,299	Model 1 N=57,031
	OR (95% CI)	OR (95% CI)
Falls	2.18 (1.92-2.46) ***	1.46 (1.13-1.87) **
Constipation	2.70 (2.29-3.16) ***	1.58 (1.08-2.24) *
Urinary retention	2.64 (2.12-3.26) ***	1.46 (0.83-2.37) §
CNS Depression	3.02 (2.47-3.66) ***	1.50 (0.88-2.37) §
Bleeding	3.88 (2.27-6.25) ***	3.18 (0.97-7.63) §
Renal injury	1.99 (1.37-2.80) ***	1.22 (0.48-2.51) §

§ : p>0.05 * : p<0.05, ** : p<0.01, *** : p<0.001
Model 1: Adjusted for age, sex and socioeconomic status

For peer review only

**Table S7. Odds ratios (with 95% CI) for taking 3 of more medications associated with similar ADRs in participants with mental health conditions (MH)**		
ADR	Self-report COPD plus MH compared with MH alone (no COPD) N=36,949	GOLD COPD plus MH compared with MH alone (no COPD) N=36126
	Model 1 N=36,885	Model 1 N=36,065
	OR (95% CI)	OR (95% CI)
Falls	2.21 (1.90-2.56) ***	1.35 (0.99-1.82) §
Constipation	2.33 (1.93-2.81) ***	1.62 (1.08-2.34) *
Urinary retention	2.17 (1.71-2.74) ***	1.42 (0.82-2.29) §
CNS Depression	2.53 (2.04-3.12) ***	1.66 (1.03-2.54) *
Bleeding	2.86 (1.77-4.17) ***	1.94 (0.76-4.05) §
Renal injury	1.86 (1.19-2.79) **	1.27 (0.45-2.80) §
§ : p>0.05 * : p<0.05, ** : p<0.01, *** : p<0.001<br>Model 1: Adjusted for age, sex and socioeconomic status		

**Table S8. Odds ratios (with 95% CI) for taking 3 of more medications associated with similar ADRs in participants with painful conditions**		
ADR	Self-report COPD plus painful conditions compared with painful conditions alone (no COPD) N=83,992	GOLD COPD plus painful conditions compared with painful conditions alone (no COPD) N=82,388
	Model 1 N=83,895	Model 1 N=82,294
	OR (95% CI)	OR (95% CI)
Falls	1.99 (1.79-2.19) ***	1.45 (1.19-1.75) ***
Constipation	2.54 (2.21-2.91) ***	1.50 (1.10-2.00) **
Urinary retention	2.46 (2.03-2.96) ***	1.11 (0.64-1.75) §
CNS Depression	2.71 (2.28-3.21) ***	1.40 (0.90-2.06) §
Bleeding	3.50 (2.37-5.01) ***	2.20 (0.86-4.54) §
Renal injury	1.66 (1.30-2.09) ***	1.49 (0.93-2.25) §
§ : p>0.05 * : p<0.05, ** : p<0.01, *** : p<0.001<br>Model 1: Adjusted for age, sex and socioeconomic status		

For peer review only

Appendix 4: Specific medications in UK Biobank participants with and without COPD

Medications	Control n=494323 count (%)	Self-report COPD n=8317 count (%)	GOLD COPD			
			All n=2620 count (%)	Mild n=399 count (%)	Moderate n=1409 count (%)	Severe n= 812 count (%)
**Total number of medications**						
≥1	356406 (72.1)	7670 (92.2)	2452 (93.6)	352 (88.2)	1321 (93.8)	779 (95.9)
≥5	87286 (17.7)	4312 (51.8)	1349 (51.5)	171 (42.9)	702 (49.8)	476 (58.6)
≥10	10678 (2.2)	1269 (15.3)	329 (12.6)	31 (7.8)	172 (12.2)	126 (15.5)
**Respiratory**						
Short acting B_2 agon.	22615 (4.6)	3328 (40.0)	1245 (47.5)	123 (30.8)	614 (43.6)	508 (62.6)
LABA	9819 (2.0)	2357 (28.3)	905 (34.5)	93 (23.3)	411 (29.2)	401 (49.3)
LAMA	597 (0.1)	1345 (16.2)	581 (22.2)	33 (8.3)	265 (18.8)	283 (34.9)
ICS	15309 (3.1)	2638 (31.7)	962 (36.7)	98 (24.6)	471 (33.4)	393 (48.4)
LABA+ICS	7259 (1.5)	1842 (22.1)	699 (26.7)	67 (16.8)	313 (22.2)	319 (39.3)
Prednisolone	3127 (0.6)	280 (3.4)	82 (3.1)	12 (3.0)	27 (1.9)	43 (5.3)
Mucolytic	174 (0.04)	187 (2.2)	49 (1.9)	1 (0.3)	10 (0.7)	38 (4.7)
**Cardiovascular**						
Antiplatelet	21817 (4.4)	894 (10.7)	268 (10.2)	31 (7.8)	158 (11.2)	79 (9.7)
ACE-inhibitor	44991 (9.1)	1276 (15.3)	367 (14.0)	33 (8.3)	198 (14.1)	136 (16.7)
ARB	17911 (3.6)	565 (6.8)	159 (6.1)	17 (4.3)	83 (5.9)	59 (7.2)
Calcium CB	14317 (2.9)	627 (7.5)	196 (7.5)	23 (5.8)	106 (7.5)	67 (8.3)
Statin	73439 (14.9)	2278 (27.4)	707 (27.0)	72 (18.0)	395 (28.0)	240 (29.6)
GTN	4425 (0.9)	373 (4.5)	110 (4.2)	8 (2.0)	70 (5.0)	32 (3.9)
ISMN	2814 (0.6)	244 (2.9)	68 (2.6)	5 (1.3)	42 (3.0)	21 (2.6)
Loop diuretic	4836 (1.0)	415 (5.0)	107 (4.1)	10 (2.5)	51 (3.6)	46 (5.7)
Thiazide	21961 (4.4)	637 (7.7)	196 (7.5)	22 (5.5)	108 (7.7)	66 (8.1)
Warfarin	4934 (1.0)	238 (2.9)	67 (2.6)	6 (1.5)	33 (2.3)	28 (3.4)
**Diabetes**						
Insulin	4643 (0.9)	161 (1.9)	35 (1.3)	2 (0.5)	23 (1.6)	3 (0.4)
Metformin	13754 (2.8)	448 (5.4)	102 (3.9)	7 (1.8)	57 (4.0)	38 (4.7)
Sulphonylurea	4901 (1.0)	158 (1.9)	35 (1.3)	2 (0.5)	17 (1.2)	16 (2.0)
Thiazolidindione	2212 (0.4)	60 (0.7)	17 (0.6)	1 (0.3)	10 (0.7)	6 (0.7)
**Gastrointestinal**						
PPI	42012 (8.5)	1989 (23.9)	522 (19.9)	79 (19.8)	286 (20.3)	157 (19.3)
Antacid	2435 (0.5)	146 (1.8)	25 (1.0)	7 (1.8)	10 (0.7)	8 (1.0)
H_2 RA.	7772 (1.6)	325 (3.9)	89 (3.4)	15 (3.8)	53 (3.8)	21 (2.6)
Laxative	5787 (1.8)	317 (3.8)	81 (3.1)	11 (2.8)	40 (2.8)	30 (3.7)
**Pain**						
Paracetamol	82376 (16.7)	2752 (33.1)	790 (30.2)	111 (27.8)	446 (31.6)	233 (28.7)
NSAID	45909 (9.3)	1149 (13.8)	319 (12.2)	50 (12.5)	175 (12.4)	94 (11.6)
Weak opiate	18736 (3.8)	1209 (14.5)	336 (12.8)	48 (12.0)	191 (13.6)	97 (11.9)
Strong opiate	1071 (0.2)	106 (1.8)	32 (1.2)	5 (1.3)	16 (1.1)	11 (1.4)
**Mental health**						
SSRI+related	15394 (3.1)	747 (9.0)	175 (6.7)	31 (7.8)	100 (7.1)	44 (5.4)
Tricyclic	4229 (0.9)	206 (2.5)	49 (1.9)	11 (2.8)	25 (1.8)	13 (1.6)
Antipsychotic	2237 (0.5)	107 (1.3)	30 (1.1)	5 (1.3)	16 (1.1)	9 (1.1)
Benzodiazepine	2316 (0.5)	182 (2.2)	47 (1.8)	6 (1.5)	28 (2.0)	13 (1.6)
**Metabolic**						
Thyroxine	20980 (4.2)	560 (6.7)	150 (5.7)	27 (6.8)	92 (6.5)	31 (3.8)
Bisphosphonate	3655 (0.7)	189 (2.3)	66 (2.5)	15 (3.8)	32 (2.3)	19 (2.3)

For peer review only

STROBE 2007 (v4) Statement—Checklist of items that should be included in reports of *cross-sectional studies*

Section/Topic	Item #	Recommendation	Reported on page #
**Title and abstract**	1	(*a*) Indicate the study's design with a commonly used term in the title or the abstract	2
		(*b*) Provide in the abstract an informative and balanced summary of what was done and what was found	2,3
**Introduction**			
Background/rationale	2	Explain the scientific background and rationale for the investigation being reported	4
Objectives	3	State specific objectives, including any prespecified hypotheses	5
**Methods**			
Study design	4	Present key elements of study design early in the paper	5,6,9
Setting	5	Describe the setting, locations, and relevant dates, including periods of recruitment, exposure, follow-up, and data collection	6,7
Participants	6	(*a*) Give the eligibility criteria, and the sources and methods of selection of participants	7
Variables	7	Clearly define all outcomes, exposures, predictors, potential confounders, and effect modifiers. Give diagnostic criteria, if applicable	6-8
Data sources/ measurement	8*	For each variable of interest, give sources of data and details of methods of assessment (measurement). Describe comparability of assessment methods if there is more than one group	6-8
Bias	9	Describe any efforts to address potential sources of bias	*14,15*
Study size	10	Explain how the study size was arrived at	7
Quantitative variables	11	Explain how quantitative variables were handled in the analyses. If applicable, describe which groupings were chosen and why	6,7,8,9
Statistical methods	12	(*a*) Describe all statistical methods, including those used to control for confounding	8-10
		(*b*) Describe any methods used to examine subgroups and interactions	9,10
		(*c*) Explain how missing data were addressed	10
		(*d*) If applicable, describe analytical methods taking account of sampling strategy	n/a
		(*e*) Describe any sensitivity analyses	9,10
**Results**			

For peer review only

Participants	13*	(a) Report numbers of individuals at each stage of study—eg numbers potentially eligible, examined for eligibility, confirmed eligible, included in the study, completing follow-up, and analysed	11, figure 1
		(b) Give reasons for non-participation at each stage	11, Figure 1
		(c) Consider use of a flow diagram	Figure 1
Descriptive data	14*	(a) Give characteristics of study participants (eg demographic, clinical, social) and information on exposures and potential confounders	Table 1
		(b) Indicate number of participants with missing data for each variable of interest	Table 1
Outcome data	15*	Report numbers of outcome events or summary measures	11-13, figure 2, appendix 3
Main results	16	(*a*) Give unadjusted estimates and, if applicable, confounder-adjusted estimates and their precision (eg, 95% confidence interval). Make clear which confounders were adjusted for and why they were included	11-13, figure 2, appendix 3
		(*b*) Report category boundaries when continuous variables were categorized	12,13
		(*c*) If relevant, consider translating estimates of relative risk into absolute risk for a meaningful time period	n/a
Other analyses	17	Report other analyses done—eg analyses of subgroups and interactions, and sensitivity analyses	12,13, Appendix 3,
**Discussion**			
Key results	18	Summarise key results with reference to study objectives	14
Limitations	19	Discuss limitations of the study, taking into account sources of potential bias or imprecision. Discuss both direction and magnitude of any potential bias	14,15
Interpretation	20	Give a cautious overall interpretation of results considering objectives, limitations, multiplicity of analyses, results from similar studies, and other relevant evidence	15,16
Generalisability	21	Discuss the generalisability (external validity) of the study results	15,16,17
**Other information**			
Funding	22	Give the source of funding and the role of the funders for the present study and, if applicable, for the original study on which the present article is based	17,18

*Give information separately for cases and controls in case-control studies and, if applicable, for exposed and unexposed groups in cohort and cross-sectional studies.

Note: An Explanation and Elaboration article discusses each checklist item and gives methodological background and published examples of transparent reporting. The STROBE checklist is best used in conjunction with this article (freely available on the Web sites of PLoS Medicine at http://www.plosmedicine.org/, Annals of Internal Medicine at http://www.annals.org/, and Epidemiology at http://www.epidem.com/). Information on the STROBE Initiative is available at www.strobe-statement.org.

For peer review only
